# RNA helicase domains of viral origin in proteins of insect retrotransposons: possible source for evolutionary advantages

**DOI:** 10.7717/peerj.3673

**Published:** 2017-08-16

**Authors:** Sergey Y. Morozov, Ekaterina A. Lazareva, Andrey G. Solovyev

**Affiliations:** 1 Belozersky Institute of Physico-Chemical Biology, Moscow State University, Moscow, Russia; 2 Department of Virology, Biological Faculty, Moscow State University, Moscow, Russia; 3 Institute of Molecular Medicine, Sechenov First Moscow State Medical University, Moscow, Russia

**Keywords:** Retrotransposon, Horizontal gene transfer, Herbivory, Gene silencing, Long interspersed nuclear element, Insect genome, Viral RNA helicase

## Abstract

Recently, a novel phenomenon of horizontal gene transfer of helicase-encoding sequence from positive-stranded RNA viruses to LINE transposons in insect genomes was described. TRAS family transposons encoding an ORF2 protein, which comprised all typical functional domains and an additional helicase domain, were found to be preserved in many families during the evolution of the order Lepidoptera. In the present paper, in species of orders Hemiptera and Orthoptera, we found helicase domain-encoding sequences integrated into ORF1 of retrotransposons of the Jockey family. RNA helicases encoded by transposons of TRAS and Jockey families represented separate brunches in a phylogenetic tree of helicase domains and thus could be considered as independently originated in the evolution of insect transposons. Transcriptome database analyses revealed that both TRAS and Jockey transposons encoding the helicase domain represented transcribed genome sequences. Moreover, the transposon-encoded helicases were found to contain the full set of conserved motifs essential for their enzymatic activities. Taking into account the previously reported ability of RNA helicase encoded by TRAS ORF2 to suppress post-transcriptional RNA silencing, we propose possible scenarios of evolutionary fixation of actively expressed functional helicases of viral origin in insect retrotransposons as genetic elements advantageous for both transposons and their insect hosts.

## Introduction

It is commonly accepted that eukaryotic genomes contain sequences derived from viruses with RNA genomes. RNA-to-DNA conversion of such sequences, a necessary step preceding the integration into the cell genomic DNA, could have only been accomplished by retrovirus or retrotransposon reverse transcriptase provided *in trans*. Due to ubiquitous occurrence of retrotransposons in eukaryotic genomes and their activity in germline cells, examples of RNA virus sequences integrated into the host genome and inherited as host alleles are generally attributed to the functional activity of retrotransposons (Holmes, 2011; Cui & Holmes, 2012; Fort et al., 2012).

Recently, we described a new group of insect retrotransposons, related to the R1 clade of Long Interspersed Nuclear Elements (LINEs). The open reading frame 2 (ORF2) protein encoded by R1 LINEs of this group contains an additional C-terminal domain similar to NTPase/helicase domains (superfamily 1 helicase - SF1H), which are found in the replicative proteins encoded by many positive-stranded RNA viruses (Lazareva et al., 2015). According to previously published data, the SF1H-encoding LINEs were found only in the order Lepidoptera. Interestingly, the genome of *Plutella xylostella* (Plutellidae) contains the highest number (several dozens) of SF1H-encoding LINEs, showing that in specific lepidopteran lineages they underwent a transpositional burst, while in some other genera these elements were subjected to complete or partial deletions (Lazareva et al., 2015).

To counteract the RNA silencing, most viruses have evolved viral suppressors of RNA silencing (VSRs), proteins that block one or more steps in the RNA silencing pathway. VSRs were first identified in plant viruses and later found in viruses infecting other higher eukaryotes (Axtell, 2013; Csorba, Kontra & Burgyán, 2015). The LINEs described in our recent paper (Lazareva et al., 2015) carry a sequence encoding SF1H domain significantly related to the VSR domain of replicases of plant positive-stranded RNA viruses belonging to the genus *Tobamovirus* of the family Virgaviridae. Our experimental data demonstrated that the predicted *P. xylostella* LINE VSR domain exhibits a weak, compared to the potent plant virus VSR p19, but detectable ability to suppress RNA silencing in the *Nicotiana benthamiana* leaves (Lazareva et al., 2015). In this context, it is important that plant and insect VSRs can substitute for each other in different eukaryotic model systems (Jing et al., 2011; Maliogka et al., 2012; Zhu et al., 2012). Moreover, the plant VSRs were shown to suppress retrotransposon silencing in heads and ovaries of insects by endogenous siRNAs (Berry et al., 2009). We supposed that both siRNA- and piRNA-mediated pathways (Ito, 2012; Peng & Lin, 2013) can be suppressed by the LINE-encoded tobamovirus-like VSR. The tobamovirus VSRs are known to function to sequester RNA duplexes and interfering with their incorporation into effector AGO complexes (Csorba et al., 2007; Wang et al., 2012). Similar silencing suppression mechanism may be anticipated for the LINE-encoded SF1H domains.

We hypothesized that the acquired SF1H-related VSR could give LINEs the ability to suppress RNA silencing and thus counteract the RNA silencing-based insect defense against retrotransposons (Lazareva et al., 2015).

In this paper, we further analyzed SF1H domains in insect genomes. Particularly, we demonstrated that TRAS ORF2-encoded SF1H are more closely related to helicase domains of several recently sequenced insect viruses than to plant viruses as it was proposed in our previous paper (Lazareva et al., 2015). Recent sequencing of around 1,500 new invertebrate RNA viruses (Shi et al., 2016) resulted in significant increase of new insect positive-stranded RNA viruses obviously related to previously better studied plant virus taxons including virga-like, beny-like, flexi-like and macula-like viruses. Nevertheless, it was found that “Despite the presence of conserved RdRp sequences, the evolutionary histories of the structural and non-structural parts of the virus genomes characterized here often differed substantially” (Shi et al., 2016).

In addition, using the helicase sequences as baits for database searches, we found that insect retrotransposons of Jockey family can encode the SF1H domain in their ORF1. We further revealed conservation of the full set of helicase conserved motifs in SF1H domains encoded by insect retrotransposons and demonstrated, by analysis of transcriptome databases, that these sequences are actively expressed in insects. In view of these findings, we propose a number of evolutionary scenarios for the acquisition and natural selection-supported preservation of SF1H domains in insect retrotransposons.

## Materials and Methods

Sequences for comparative analysis were retrieved from NCBI (http://www.ncbi.nlm.nih.gov/). The nucleic acid sequences and deduced amino acid sequences were analyzed and assembled using the NCBI. BLAST searches were carried out using the NCBI server with all available databases. An ORF search in retrotransposons was performed with the ORF Finder of the NCBI. Conserved domains in the amino acid sequences were identified using the CD-Search of the NCBI. COBALT, the constraint-based alignment tool for multiple protein sequences (http://www.ncbi.nlm.nih.gov/tools/cobalt/) was used for multiple sequence alignments and phylogenetic analyses; neighbor-joining trees were obtained with the use of default parameters.

We also used a popular motif-finding tool WebLogo 3 (version 3.5.0.) (http://weblogo.threeplusone.com/) to find the characteristic motifs of retrotransposon SF1H proteins. The secondary structures of the proteins were modeled with the PCOIL (http://toolkit.tuebingen.mpg.de/ pcoils) program.

## Results

### Retrotransposon-encoded RNA helicase domains are related to replicative SF1H helicases of both invertebrate and plant viruses

The unexpected occurrence of viral-like helicase and insect retrotransposon protein domains combined in a single polypeptide raised questions on the evolutionary origin of such proteins. First, was there an event of horizontal gene transfer (HGT) of the SF1H domain-coding sequence directly from plant viruses to insect retroelements, or such HGT occurred from unknown insect viruses coding for SF1H domains similar to those in tobamovirus protein? Second, could the SF1H HGT to insect chromosomes results in its integration in locations other that the ORF2 of TRAS retrotransposons and, if it occurred, what is the relation of such differently located SF1H sequences to the lepidopteran TRAS ORF2 SF1H? Enormous increase of invertebrate virus-like sequences (including insect viruses) in public databases during the last year (Shi et al., 2016; Webster et al., 2016; Nunes et al., 2017) enabled us to address these questions by performing, using NCBI databases, new comparative sequence analyses of viral RNA helicases and those encoded by lepidopteran TRAS ORF2.

**Figure 1:**
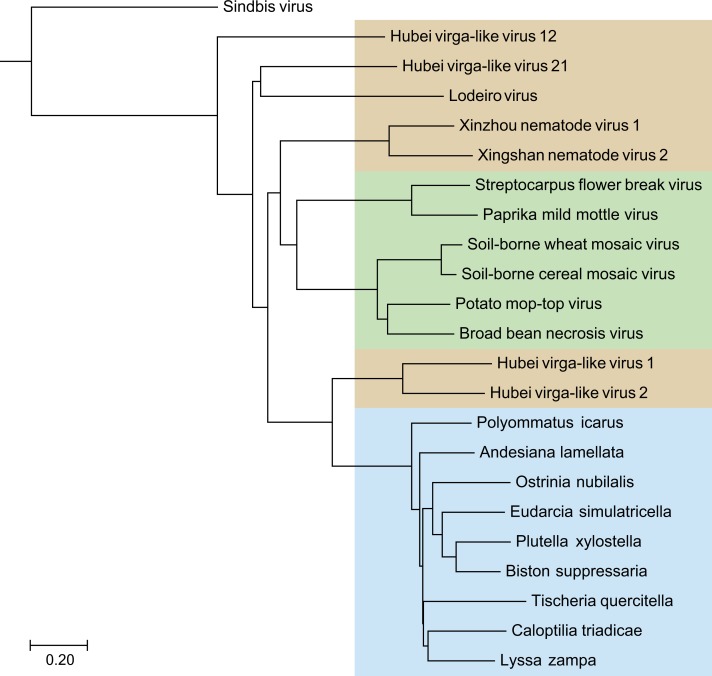
The phylogenetic tree based on sequence alignment of the analyzed SF1H proteins of Lepidoptera transposons and some insect and plant viruses. Neighbor-joining tree was obtained at http://www.ncbi.nlm.nih.gov/tools/cobalt/ with the use of default parameters. Sindbis virus SF1H was used as outgroup. Plant viruses are shown by green shading. Invertebrate viruses are by brown shading. Selected lepidopteran species with transposons coding for SF1H are shown by blue shading. The scale bar denotes the estimated number of amino acid substitutions per site.

Blast analyses, with the deduced SF1H amino acid sequences from nine TRAS elements of the selected Lepidoptera species as queries, against the NCBI database revealed that these sequences showed highest identities (37–44%) with helicase domains of replicative ORF1 proteins of Hubei virga-like viruses 1 and 2 isolated from mosquitoes in China (Shi et al., 2016) (Table 1). Some other invertebrate viruses (Xinzhou nematode virus 1, Lodeiro virus from spiders and Xingshan nematode virus 2) also showed significant similarities of their replicative polypeptides to TRAS SF1H domains, whereas tobamoviruses and some other plant Virgaviruses had somewhat lower similarity scores (identity 34–35%) (Table 1). In general, a neighbor-joining tree obtained with the NCBI COBALT service clearly indicated that all TRAS SF1H domains clustered as single brunch with high bootstrap values (Fig. 1), and that helicase domains of ORF1 proteins of Hubei virga like viruses 1 and 2 are most similar to lepidopteran TRAS SF1H domains, whereas plant Virgaviruses form a separate brunch of the helicase protein tree.

**Table 1:** Amino acid sequence comparisons of some SF1H proteins encoded by LINEs in Lepidoptera and RNA virus replicative helicases.

Query Lepidoptera species	Subject viral replication protein	*E*-value	Maximal amino acid identity (%)	Accession numbers (NCBI)
*Andesiana lamellata* (Andesianidae)	Hubei virga-like virus 1	**1e−47**	**42**	**YP_009337423**
_***_	Hubei virga-like virus 2	**6e−47**	**42**	**YP_009337412**
_***_	Xinzhou nematode virus 1	**1e−30**	**36**	**YP_009345041**
_***_	Xingshan nematode virus 2	**1e−30**	**35**	**YP_009345038**
*Tischeria quercitella* (Tischeriidae)	Hubei virga-like virus 1	**2e−40**	**38**	**YP_009337423**
_***_	Hubei virga-like virus 2	**9e−40**	**38**	**YP_009337412**
_***_	Lodeiro virus	**1e−27**	**33**	**YP_009315901**
_***_	Hubei virga-like virus 12	**2e−27**	**32**	**YP_009337818**
*Eudarcia simulatricella* (Tineidae)	Hubei virga-like virus 1	**1e−43**	**40**	**YP_009337423**
_***_	Hubei virga-like virus 2	**1e−41**	**38**	**YP_009337412**
_***_	Lodeiro virus	**3e−31**	**33**	**YP_009315901**
_***_	Potato mop-top virus	**4e−31**	**34**	**ALM54963**
*Caloptilia triadicae* (Gracillariidae)	Hubei virga-like virus 1	**8e−38**	**39**	**YP_009337423**
_***_	Hubei virga-like virus 2	**3e−36**	**37**	**YP_009337412**
_***_	Lodeiro virus	**9e−28**	**33**	**YP_009315901**
_***_	Soil-borne wheat mosaic virus	**3e−26**	**34**	**BAA94796**
*Plutella xylostella* (Plutellidae)	Hubei virga-like virus 1	**1e−47**	**44**	**YP_009337423**
_***_	Hubei virga-like virus 2	**1e−44**	**39**	**YP_009337412**
_***_	Xinzhou nematode virus 1	**3e−32**	**39**	**YP_009345041**
_***_	Broad bean necrosis virus	**2e−29**	**35**	**NP_740761**
*Ostrinia nubilalis* (Crambidae)	Hubei virga-like virus 1	**7e−45**	**41**	**YP_009337423**
_***_	Hubei virga-like virus 2	**2e−41**	**41**	**YP_009337412**
_***_	Xinzhou nematode virus 1	**4e−31**	**37**	**YP_009345041**
_***_	Soil-borne cereal mosaic virus	**1e−30**	**35**	**AAF18326**
*Polyommatus icarus* (Lycaenidae)	Hubei virga-like virus 1	**5e−48**	**40**	**YP_009337423**
_***_	Hubei virga-like virus 2	**1e−45**	**41**	**YP_009337412**
_***_	Lodeiro virus	**3e−37**	**37**	**YP_009315901**
_***_	Hubei virga-like virus 21	**5e−34**	**37**	**YP_009337659**
*Lyssa zampa* (Uraniidae)	Hubei virga-like virus 1	**1e−43**	**38**	**YP_009337423**
_***_	Hubei virga-like virus 2	**1e−41**	**38**	**YP_009337412**
_***_	Streptocarpus flower break virus	**2e−27**	**34**	**YP_762618**
_***_	Lodeiro virus	**3e−30**	**32**	**YP_009315901**
*Biston suppressaria* (Geometridae)	Hubei virga-like virus 1	**3e−47**	**42**	**YP_009337423**
_***_	Hubei virga-like virus 2	**2e−44**	**37**	**YP_009337412**
_***_	Xinzhou nematode virus 1	**1e−30**	**35**	**YP_009345041**
_***_	Paprika mild mottle virus	**2e−28**	**34**	**ANV28177**

**Notes.**

Plant viruses are in green. Insect families are in indicated in parenthesis.

**Figure 2:**
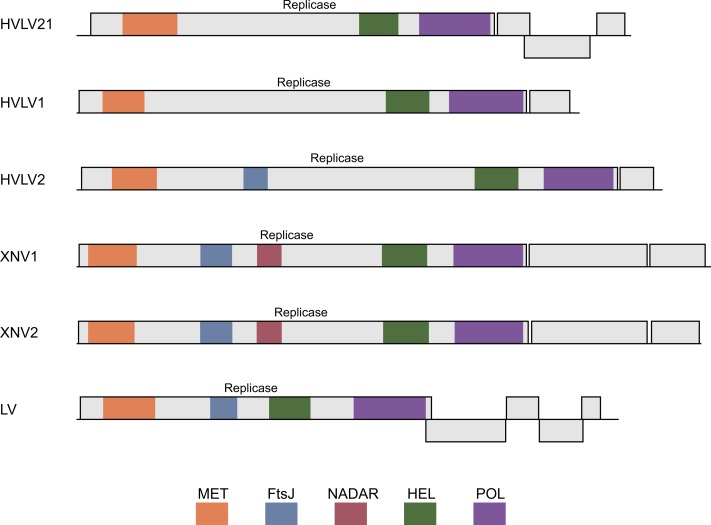
Schematic ORF organization depicting genomic RNAs of analyzed invertebrate viruses. Replicase protein domains are indicated in different colors and abbreviated according to the text. HVL21, Hubei virga-like virus 21; HVL1, Hubei virga-like virus 1; HVL2, Hubei virga-like virus 2; XNV1, Xinzhou nematode virus 1; XNV2, Xingshan nematode virus 2; LV, Lodeiro virus.

**Figure 3:**
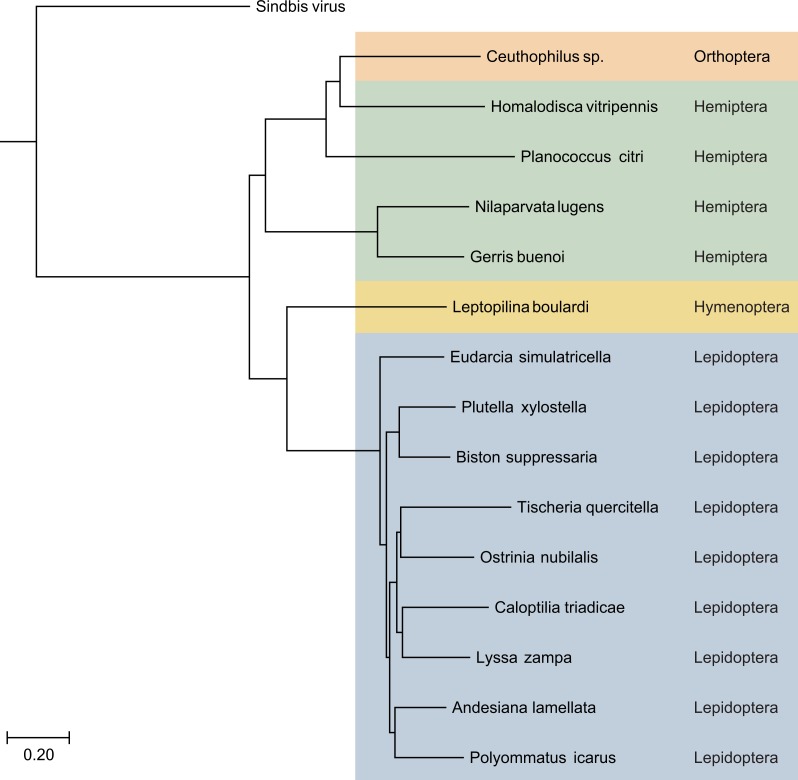
The phylogenetic tree based on sequence alignment of the analyzed SF1H proteins encoded by Lepidoptera TRAS-like LINEs and Jockey-like LINEs of three other insect orders. Taxonomic positions of the insect species are indicated on the right and highlighted by different colors. Neighbor-joining tree was obtained at http://www.ncbi.nlm.nih.gov/tools/cobalt/ with the use of default parameters. Sindbis virus SF1H was used as outgroup. The scale bar denotes the estimated number of amino acid substitutions per site.

The genomes of invertebrate viruses encoding helicase domains most closely related to TRAS ORF2 SF1H (Table 1) encode two to five proteins (Fig. 2). In all cases, the ORF1 polyprotein represents a viral polymerase protein and shows obvious similarity with the replicative proteins of negeviruses, Boutonnet virus and Adelphocoris suturalis-associated virus 1 (Shi et al., 2016; Li et al., 2017; Nunes et al., 2017). In different viruses, a several conserved domains are observed in ORF1 proteins. These domains are related to the highly conserved sequences of viral methyltransferase (PFAM: PF01660), ribosomal RNA methyltransferase FtsJ domain, (PFAM: PF01728), viral SF1 helicase (PFAM: PF01443), and the domain of RNA-dependent RNA polymerase (RdRp) (PFAM: PF00978) (Fig. 2). It was shown, that among the invertebrate virga-like viruses, RdRp domains of Hubei virga-like viruses 1 and 2 form a distinct cluster which contains proteins most similar to plant Sindbis-like virus replicative proteins (Supplementary data 3 in Shi et al., 2016). On the other hand, the ORF1 polyprotein of other invertebrate virga-like viruses shows obvious similarity with negeviruses, Boutonnet virus and Adelphocoris suturalis-associated virus 1 (Shi et al., 2016; Li et al., 2017; Nunes et al., 2017). Thus our previous conclusion on the closest relationship between TRAS ORF2 SF1H and replicative helicases of plant tobamoviruses (Lazareva et al., 2015) is explained by incompleteness of sequence data available at that time.

### Virus-like RNA helicase domains are found in ORF1 of non-LTR retrotransposons

**Figure 4:**
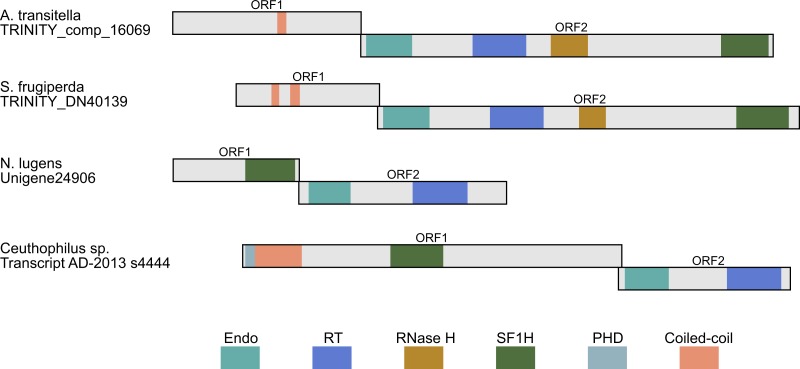
Schematic ORF organization depicting proteins encoded by analyzed TRAS-like elements of *A. transitella* and *S. frugiperda* and Jockey-like LINE elements of *N. lugens* and *Ceuthophilius* sp. Conserved domains of ORF1 and ORF2 proteins are indicated in different colors and abbreviated according to the text.

Using replicative SF1H domains of recently sequenced invertebrate viruses (Shi et al., 2016) as baits, we performed more careful mining nucleotide sequence databases in an attempt to reveal sequences coding for polypeptides related to viral SF1H protein in insect orders outside Lepidoptera. We used concomitant TBLASTN searches using viral SF1H, reverse transcriptase (RT) and endonuclease domains as baits. Using this approach, new retrotransposons with the full-length viral SF1H-coding sequences were found in several insect transcriptomic and genomic assemblies (Fig. 3). In whole-genome shotgun contigs of rice pest brown planthopper *Nilaparvata lugens* (Hemiptera: Delphacidae), the draft genome of which has been recently published (Xue et al., 2014), we revealed dozens of sequences, where SF1H-containing ORFs are located very close to or overlap with ORFs encoding proteins showing a typical organization of LINE-encoded ORF2 polyprotein and containing the RT and endonuclease domains of retrotransposons belonging to Jockey superfamily (Table 2). Particularly, contig AOSB01072940 (Unigene24906) contains an ORF coding for a protein with a single domain related to SF1H and overlapping ORF2 by 2 nucleotides (Fig. 4). An almost identical organization was found for a Jockey-like LINE element in contig AOSB01047371.

**Table 2:** Amino acid sequence comparisons of some insect LINE ORF2 proteins and those encoded by SF1H-coding LINEs in Hemiptera and Orthoptera species.

Query Hemiptera and Orthoptera species	Subject insect LINE ORF2 protein	*E*-value	Maximal amino acid identity **(%)**	Accession numbers (NCBI)
*Ceuthophilus* sp. GAUX01000930 (Orthoptera)	Mobile element jockey-like [Papilio xuthus]	**1e−60**	**29**	**XP_013171417**
_***_	Mobile element jockey-like [Papilio machaon]	**4e−56**	**29**	**XP_014357830**
_***_	Mobile element jockey- like [Amyelois transitella]	**3e−51**	**28**	**XP_013193561**
_***_	Mobile element jockey-like [Vollenhovia emeryi]	**5e−46**	**28**	**XP_011859003**
*Homalodisca vitripennis* JJNS01051000 (Hemiptera)	Mobile element jockey-like [Diachasma alloeum]	**1e−06**	**27**	**XP_015119810**
_***_	Mobile element jockey-like [Papilio xuthus]	**6e−06**	**24**	**XP_013171417**
_***_	Uncharacterized protein LOC103522538 [Diaphorina citri]	**1e−05**	**24**	**XP_008485861**
_***_	Mobile element jockey-like [Papilio machaon]	**2e−05**	**25**	**XP_014357830**
*Nilaparvata lugens* AOSB01052258 (Hemiptera)	Mobile element jockey-like isoform X1 [Amyelois transitella]	**1e−148**	**35**	**XP_013193561**
_***_	Mobile element jockey-like [Papilio xuthus]	**1e−49**	**26**	**XP_013171417**
_***_	Uncharacterized protein LOC106650627 [Trichogramma pretiosum]	**6e−48**	**25**	**XP_014224251**
_***_	Transposon X-element [Tribolium castaneum]	**8e−47**	**26**	**XP_973868**
*Gerris buenoi* JHBY01062481 (Hemiptera)	Mobile element jockey-like [Amyelois transitella]	**4e−29**	**32**	**XP_013193561**
_***_	Mobile element jockey-like [Neodiprion lecontei]	**2e−17**	**29**	**XP_015522510**
_***_	Uncharacterized protein LOC105842132 [Bombyx mori]	**1e−11**	**26**	**XP_012548769**
_***_	Mobile element jockey-like [Diachasma alloeum]	**7e−10**	**25**	**XP_015124772**

In general, the LINE retrotransposon ORF1 is more variable than ORF2. Although ORF1 was often considered as a possible equivalent of the retroviral *gag* gene, the functions of the ORF1 are less understood, and their sequences in different LINEs are highly variable (Malik, Burke & Eickbush, 1999; Goodier & Kazazian jr, 2008). Analysis of ORF1-encoded proteins from a dozen of LINE clades revealed several ORF1 classes based on the types of conserved domains and their positions. Particularly, these domains may be represented by RNA recognition motif (RRM), CCHC-type knuckle, a coiled-coil domain, PHD sequence (plant homeodomain) and esterase domain (Malik, Burke & Eickbush, 1999; Khazina & Weichenrieder, 2009; Metcalfe & Casane, 2014; Gaurav et al., 2017). Our data show that the ORF1-encoded proteins may contain also the SF1H domain (Fig. 4).

Another identified insect encoding LINE with the SF1H domain in the ORF1 was glassy-winged sharpshooter *Homalodisca vitripennis* (Hemiptera, Cicadellidae), a xylem-feeding leafhopper. The transcriptome of adult *H. vitripennis* was explored using high-throughput sequencing and *de novo* assembly (Nandety et al., 2013). Among transcript assemblies of *H. vitripennis,* we revealed sequences (particularly, scaffold JJNS01178034) organized similarly to contigs of *Nilaparvata lugens*. These sequences also represent Jockey-like LINEs (Fig. 4).

Using the same approach, we identified ORF1 encoding SF1H in additional species from orders Hemiptera and Orthoptera (Fig. 3 and Table 2). Particularly, genomes of insects from genus *Ceutophilius* (camel crickets), representing one of the most basal insect orders, namely, Orthoptera (Misof et al., 2014), also contain a Jockey-like LINE element encoding an ORF1 with SF1H domain (Fig. 4, Tables 2 and 3).

**Table 3:** Search for transcribed sequences of SF1H encoded by retrotransposon-related ORFs in Lepidoptera and other insects.

Order/Suborder	Family	Subfamily	Species	Sequence source
Lepidoptera	Agathiphagidae	–	*Agathiphaga queenslandensis*	SRX1594824
Aglossata				
Lepidoptera	Heterobathmiidae	–	*Heterobathmia pseuderiocrania*	SRX1594810
Heterobathmiina				
Lepidoptera	Acanthopteroctetidae	–	*Acanthopteroctetes unifascia*	SRX1594806
Glossata (Dacnonypha^a^)				
Glossata (Dacnonypha^a^)	Lophocoronidae	–	*Lophocorona astiptica*	SRX1594812
Lepidoptera	Neopseustidae	–	*Neopseustis meyricki*	SRX1594818
Glossata (Myoglossata^a^)				
Lepidoptera	Hepialidae	–	*Hepialus xiaojinensis*	SRX2583878
Glossata (Neolepidoptera, Exoporia^b^)				
-/-	-/-	–	*Thitarodes jiachaensis*	SRX862112
Glossata (Neolepidoptera, Heteroneura^b^)	Andesianidae	–	*Andesiana lamellata*	GEOA01069083
-/-	Tischeriidae	–	*Tischeria quercitella*	GEOU01072667
				GENO01015855
-/-	Tineidae	Dryadaulinae	*Dryadaula visaliella*	GENH01137414
-/-	-/-	Meessiinae	*Eudarcia simulatricella*	GEOF01053845
-/-	-/-	Tineinae	*Tineola bisselliella*	GEOR01006141
-/-	Gracillariidae	Gracillariinae	*Caloptilia triadicae*	SRX869394
-/-	-/-	Lithocolletinae	*Cameraria ohridella*	SRX488063
-/-	Yponomeutidae	Yponomeutinae	*Yponomeuta evonymellus*	GASG02025483
-/-	Plutellidae	–	*Plutella xylostella*	HX687959
				HX687832
				HX685996
-/-	Elachistidae	Stenomatinae	*Antaeotricha schlaegeri*	SRX371326
-/-	Zygaenidae	Zygaeninae	*Zygaena fausta*	SRX371360
-/-	Limacodidae	–	*Euclea delphinii*	SRX371325
-/-	Castniidae	Synemoninae	*Synemon plana*	SRX362667
-/-	Urodidae	–	*Urodus decens*	SRX371357
-/-	Pyralidae	Phycitinae	*Plodia interpunctella*	ERX392603
-/-	-/-	-/-	*Amyelois transitella*	GDGN01078241
-/-	Crambidae	Pyraustinae	*Ostrinia nubilalis*	GAVD01018675
-/-	-/-	-/-	*Loxostege sticticalis*	GFCJ01034503
-/-	Noctuidae	Amphipyrinae	*Spodoptera frugiperda*	GESP01134032
-/-	-/-	Heliothinae	*Helicoverpa armigera*	GBXD01029497
-/-	-/-	Plusiinae	*Trichoplusia ni*	GBKU01050963
				GBKU01044506
-/-	Lymantriidae	–	*Lymantria dispar*	SRX1520900
-/-	Sphingidae	Sphinginae	*Manduca sexta*	GETI01156885
-/-	Saturniidae	Saturniinae	*Antheraea pernyi*	GBZF01003318
-/-	-/-	-/-	*Samia ricini*	GBZD01018504
-/-	Lycaenidae	Polyommatinae	*Polyommatus icarus*	GAST02024448
-/-	-/-	-/-	*Hemiargus ceraunus*	SRX553292
-/-	-/-	Theclinae	*Protantigius superans*	SRX1257171
-/-	-/-	Aphnaeinae	*Spindasis takanonis*	SRX1257172
-/-	Papilionidae	Papilioninae	*Papilio zelicaon*	JP709801
-/-	Nymphalidae	Heliconiinae	*Heliconius ismenius*	FAPP01000292
-/-	-/-	Nymphalinae	*Melitaea cinxia*	APLT01012297
-/-	Hesperiidae	Hesperiinae	*Lerema accius*	SRX1085019
-/-	-/-	-/-	*Thymelicus sylvestris*	SRX565325
-/-	-/-	-/-	*Hylephila phyleus*	SRX553313
-/-	-/-	Megathyminae	*Megathymus yuccae*	SRX553644
-/-	Drepanidae	Thyatirinae	*Pseudothyatira cymatophoroides*	SRX371349
-/-	Sematuridae	–	*Nothus lunus*	SRX553336
-/-	Uraniidae	Uraniinae	*Lyssa zampa*	SRX553795
-/-	-/-	Epipleminae	*Calledapteryx dryopterata*	SRX371329
-/-	Geometridae	Ennominae	*Biston suppressaria*	GCJP01006855
-/-	-/-	Larentiinae	*Operophtera brumata*	KOB69843
Hemiptera	Delphacidae	Delphacinae	*Nilaparvata lugens*	SRX698355
-/-	Cicadellidae	Cicadellinae	*Homalodisca vitripennis*	SRX910971
-/-	-/-	-/-	*Graphocephala coccinea*	SRX2141460
-/-	Gerridae	Gerrinae	*Gerris buenoi*	JHBY01062481
				SRX896710
-/-	Pseudococcidae	–	*Planococcus citri*	SRX275951
Orthoptera	Rhaphidophoridae	Ceuthophilinae	*Ceuthophilus* sp.	GAUX02053896
				GAUX02050946
-/-	-/-	-/-	*Ammobaenetes arenicolus*	SRX1203846
-/-	Acrididae	Oedipodinae	*Locusta migratoria*	SRX850791
				GBDZ01086272
Hymenoptera	Figitidae	Leptopilinae	*Leptopilina boulardi*^c^	GAJA01009526
				SRX184305
Diptera	Culicidae	Culicinae	*Aedes aegypti*	NW_001811003
				SRX1897891

**Notes.**

a infraorder.

b infraorder/superfamily.

c SF1H-encoded ORF is interrupted by termination codons

-/- indicates the same taxon as above

Recently, it was shown that a flavivirus genome region coding for SF2H RNA helicase and adjacent genes could be integrated into chromosomes of representatives of genera *Aedes* and *Anopheles* (order Diptera), where virus sequences were often positioned in the vicinity of LTR transposons (Chen et al., 2015; Lequime & Lambrechts, 2017; Suzuki et al., 2017). Our search for fusions between virus-like SF1H and proteins of LTR transposons also revealed transcribed ORF in the genome of *Aedes aegypti* which codes for SF1H domain followed by full-length RNase H domain of Ty1/Copia LTR transposons (Table 3). In order Hymenoptera, a similar fused protein of Ty1/Copia transposons was found in *Leptopilina boulardi* (family Figitidae). In this insect, SF1H ORF is fused in frame as an upstream element to the ORF coding for integrase core domain (Table 3).

### Search for transcribed sequences of SF1H encoded by LINEs in insects

Previously we reported that TRAS ORF2 sequences coding for an additional SF1H domain are actively expressed at different stages of ontogenesis in different tissues of lepidopteran *Plutella xylostella* (Lazareva et al., 2015). Here, we further explored NCBI insect transcriptome databases to assess the expression of TRAS ORF2 with encoded SF1H domains in large number of lepidopteran species and viral-like SF1H domains expressed by other insect orders. Table 3 shows that SF1H sequences are expressed in most lepidopteran species tested including whole organisms (at different development stages) and cell lines of many species excluding *Bombyx mori* (*Bombycidae*). These species include insects from most basal lepidopteran superfamilies Aglossata and Heterobathmiina, as well as from basal Glossata (infraorders Dacnonypha and Myoglossata). In order Hemiptera, transcripts coding for ORF1 with viral SF1H domain were revealed among the representatives of families Delphacidae, Cicadellidae and Gerridae (Table 3). Among Orthoptera, these transcripts were found in the families Rhaphidophoridae and Acrididae (Table 3).

**Figure 5:**
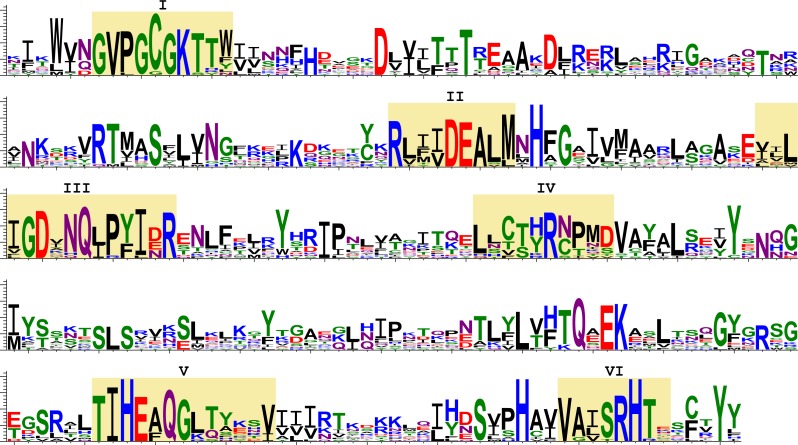
Sequence logos of the SF1H conserved domains encoded by insect LINE transposons. These sequence logos, which visualize the distribution of amino acids at each position of conserved motifs, are based on the aligned transposon-encoded SF1H sequences. Yellow shading indicates conserved motifs of SF1H proteins (I–VI). Amino acids are colored according to chemical properties; negatively charged (red), positively charged (blue). Amino acids are represented in a single-letter code.

Next, we analysed the deduced amino acid sequences of transcribed SF1H. We aligned the amino acid sequences of the SF1H domains found in insects and encoded by TRAS- and Jockey-like LINE elements (Fig. 5). Six highly conserved motifs (I–VI) were reported for the SF1H domains (Gorbalenya & Koonin, 1989; Lehmann et al., 2015). Insect viral-like helicases retain not only the most conserved motifs I and II also known as Walker A and B boxes, but also motifs which are located in the C-terminal helicase region (Fig. 5). The long-time conservation of the complete set of SF1H conserved motifs in two different types of insect non-LTR retrotransposons could be considered as a strong indication of the evolutionary preservation of SF1H functional properties.

## Discussion

### Acquisition of SF1H coding sequences providing selective advantages to retrotransposons as selfish genetic elements

The natural selection-supported presence of SF1H in both ORF1 and ORF2 of retrotransposons of two different types strongly suggests that SF1H expression by retrotransposons can increase the evolutionary fitness of these selfish genetic elements. Originally, we proposed that the VSR function provided by the SF1H domain could be of evolutionary advantage for TRAS LINEs, since their transposition may be suppressed by the RNA silencing system (Lazareva et al., 2015). LINEs and other retrotransposons are controlled by the RNA interference mechanisms at both the transcriptional and post-transcriptional levels. Transposon-specific siRNAs mainly direct local DNA methylation resulting in repressed retrotransposon transcription and, additionally, contribute to the degradation and/or translational repression of their transcripts. Another class of small RNAs, piRNAs derived from genomic PIWI loci containing multiple dysfunctional transposon sequences, act predominantly post-transcriptionally, but can take part in transcriptional regulation as well (Peng & Lin, 2013; Ito, 2012). In agreement with this general view on the role of PIWI loci in transposon control, the accumulation level of TRAS1 transcript is found to be significantly increased when piRNA pathway is compromised in insect cells (Tatsuke et al., 2010). We suppose that both siRNA- and piRNA-mediated pathways can be suppressed by the LINE-encoded SF1H VSR.

On the other hand, one cannot exclude that an advantage in evolutionary fitness might be provided to retrotransposons by the SF1H helicase function. In fact, viral SF1H proteins were able to unwind not only double-stranded RNA but also RNA-DNA duplexes and dsDNA substrates containing a single-stranded region at one or both of the 5′ ends (Lehmann et al., 2015). This means that SF1H-coding sequences acquired by retrotransposons might be adapted for co-operative work with reverse-transcribing enzymes to improve replication and transposition efficiency of selfish genetic elements.

One can also speculate that SF1H domain in insect TRAS-like LINEs may participate in post-transcriptional quality control of transposon RNA transcript. Interestingly, ORF2 of these LINEs encode zinc binding domain (homology to pfam13966: zf-RVT) upstream of SF1H domain (Lazareva et al., 2015). The location of this domain in the ORF2 protein sequence and its orientation relative to the SF1H domain resemble those of zinc binding domain in replicative RNA helicases of nidoviruses and the helicase Upf1-like subfamily (Lehmann et al., 2015). Zinc finger (approx. 30 residues) constitute the functional part of the zinc binding domain. This general organization is only found for SF1H in Upf1 of all eukaryotes and nidoviruses. For Upf1, its conservation was attributed to the universal role in post-transcriptional quality control of eukaryotic RNAs, including nonsense-mediated mRNA decay (Lehmann et al., 2015). If the insect SF1H helicases of TRAS elements possess some of the properties of Upf1, this could be connected to the unusual organization and expression of dicistronic LINE RNA transcript, where translation of the second ORF is performed by reinitiation mechanism (Alisch et al., 2006; Kojima, Matsumoto & Fujiwara, 2005). For instance, providing post-transcriptional quality control of genomic RNA, i.e., detection of long untranslated regions and nonsense-mediated mRNA decay resulting in elimination of defective molecules, the TRAS ORF2 helicase could alleviate the consequences of the low fidelity of transposon RNA synthesis and reverse transcription of full-length pre-genomic RNA.

Another possible advantage of the SF1H helicase function for retrotransposons could be inferred from recent findings showing that non-LTR retrotransposons as well as LTR retrotransposons of insects can produce both sense and anti-sense transcripts that results in formation of double-stranded RNA precursors which can be processed by Dicers into siRNAs capable of silencing the retrotransposon transcripts (Li et al., 2014; Russo, Harrington & Steiniger, 2016). Viral SF1H sequences acquired and adapted by mobile genetic elements may prevent the negative impact of this mechanism by unwinding double-stranded RNAs and therefore suppressing the generation of transposon-specific siRNAs. Importantly, the nidovirus helicase structure has two possible RNA-binding clefts, which are formed by domains 1A and 1B of SF1H and the zinc binding domain and could be especially suited for unwinding complex RNA secondary structures (Lehmann et al., 2015). The formally similar organization in the TRAS ORF2 protein suggests analogous enhancement for the mechanism of dsRNA unwinding.

### Preservation of genome-integrated virus-like SF1H coding sequences as a tool for anti-viral defense

It is well documented that negative sense single-stranded RNA virus genomes can be integrated as the full copies or gene fragments into the genomes of insect hosts including, particularly, drosophila, mosquitos and ticks (Holmes, 2011; Ballinger, Bruenn & Taylor, 2012; Fort et al., 2012). Moreover, these integrated virus sequences are actively expressed (Geisler & Jarvis, 2016). Other invertebrates also actively acquired minus-RNA viral genome sequences which are often integrated as a result of transposon-related reverse transcription and can be found in the chromosome regions enriched in retrotransposons (Ballinger, Bruenn & Taylor, 2012; Thézé et al., 2014; Metegnier et al., 2015).

Very recently it was found that minus-RNA viral genome sequences can be massively integrated into PIWI clusters producing transcripts that function in the piRNA pathway. Moreover, full-length retrotransposons are often found to flank integrated virus-related loci (Palatini et al., 2017). PIWI proteins bound to siRNAs derived from endogenous virus-related transcripts may target the genomes of close exogenous viruses upon their infection, possibly conferring selective advantage to the insects possessing acquired integrated virus sequences. In this scenario, a horizontal gene transfer event linked to the activity of retrotransposons may trigger for the functional specialization of PIWI clusters against both retrotransposon sequences and specific virus sequences (Palatini et al., 2017).

An alternative scenario implies the possibility that the protein expression from integrated virus-like sequences is able to affect the replication of exogenous viruses (Honda & Tomonaga, 2016). It can be proposed that the retrotransposon-encoded SF1H can inhibit virus replication, since the excessive RNA helicase activity provided by SF1H might cause deregulation of otherwise balanced transcription/replication of insect-infecting virga-related viruses, resulting in suppression of negative disease consequences.

### Adaptive acquisition of virus-like SF1H VSR domains by highly expressed insect genome sequences as a possible factor supporting herbivorous lifestyle

The extraordinary diversity of insects has been largely explained by the important role of co-evolution with flowering plants (Farrell & Mitter, 1998). Some authors have suggested that, among several other factors, feeding on living tissues of vascular plants is a major driver of insect diversification (Wiens, Lapoint & Whiteman, 2015). However, plants have evolved different defense strategies that negatively affect the herbivores. Plant resistance to herbivory can be achieved by physical barriers such as trichomes and waxy layer. In addition, defensive phytochemicals have been evolved to repulse and poison herbivores or interfere with the assimilation of consumed nutrients inside the insect’s gut. For example, many plants produce cyanogenic glycosides that can be converted into hydrogen cyanide when the plant is eaten (Wybouw et al., 2016). Nevertheless, insects can overcome these nutritional and defensive barriers using, particularly, the optimized assimilation and detoxification processes (Després, David & Gallet, 2007; Wybouw et al., 2014).

A recently developed pathogen control strategy, which is called host-induced gene silencing (HIGS), is based on generating transgenic plants that express pathogen-specific dsRNA to trigger silencing of essential genes in insects, fungi and other pests (Nunes & Dean, 2012; Koch & Kogel, 2014; Weiberg & Jin, 2015). Importantly, recent reports show that not only artificial transgenic HIGS dsRNAs, but also plant endogenous dsRNAs can be actively transported into insect cells; however, the functional consequences of consuming these dietary-derived plant dsRNAs for the insects remain to be clarified (Ivashuta et al., 2015; Sattar & Thompson, 2016). The natural dsRNA transfer from plants to insects was reconstituted in numerous studies of insect feeding on substrates containing artificial insect-specific dsRNAs. These experiments revealed that beetles (order Coleoptera) are very amenable to dsRNA-mediated RNA silencing, whereas other insects, most notably lepidopterans, are more refractory to RNA silencing (Swevers, Van den Broeck & Smagghe, 2013). As an explanation of the observed difference between coleopteran and lepidopteran insects in their RNA silencing response, it was proposed that persistent viral infections (and subsequently continuous synthesis of virus-encoded VSR proteins) are much more prevalent in lepidopterans than in other insects. This could be an important factor contributing to lepidopteran relative recalcitrance to RNA silencing (Swevers, Van den Broeck & Smagghe, 2013).

To our mind, this phenomenon can be rather attributed to less efficient silencing response due to the presence of VSR-encoding LINEs in Lepidoptera but not in Coleoptera insects. In agreement with this hypothesis, dsRNA administered to coleopteran cell lines and tissues (*Tribolium castaneum* and *Leptinotarsa decemlineata*) was actively processed into 23-nucleotide-long siRNA, whereas the uptake of dsRNA by lepidopteran cell lines and tissues (*Spodoptera frugiperda* and *Heliothis virescens*) did not result in detectable siRNA production (Shukla et al., 2016). Moreover, overexpression of *L. decemlineata* Argonaute-1 and Aubergine proteins, which are required for processing of dsRNA into siRNA, in *Spodoptera frugiperda* cells partly improved silencing effects induced by dsRNA (Yoon et al., 2016). This finding clearly shows that the impairment of RNA silencing in lepidopteran cell is associated with a suppression of dsRNA processing into small RNAs. Additionally, when the impact of the persistent virus infection on gene silencing induced by dsRNA was tested in two normally virus-free lepidopteran cell lines, no significant interference with artificial dsRNA-induced gene silencing was found in virus-infected cells when compared to virus-free cells (Swevers et al., 2016). These data show that the inefficient response of lepidopteran cells to dsRNA could not be attributed to persistent infections with viruses providing VSR proteins.

We suppose that these data argue in favor of the hypothesis that the continuous expression of LINE-encoded SF1H domain, which has the VSR function, in Lepidoptera insects makes them highly resistant to the negative effect of consumed plant artificial and potential endogenous dsRNAs targeted against insect genes. Therefore, assuming the existence of dsRNA-based pest defense in plants, the acquisition of VSR genes by LINEs in early evolution of Lepidoptera by HGT could have a significant positive adaptive impact in their evolution as herbivores and serve as a factor of herbivorous lifestyle expansion in insects of this order. Indeed, Lepidoptera is one of the prominent insect taxons with respect to species richness among insect orders and contains the highest proportion of herbivores (Wiens, Lapoint & Whiteman, 2015). It should be emphasized that this evolutionary scenario could take place only if the HGT-transferred VSR gene became highly expressed in the context of insect genome. In fact, RNA transcripts of SF1H VSR domain are widely represented in the tissues of most lepidopteran species at different developmental stages. Importantly, potential functional abilities of this domain, namely, unwinding of long dsRNA which could be especially enhanced because of Upf1-like organization of the C-terminal part of TRAS ORF2 protein (Lehmann et al., 2015; Lazareva et al., 2015, see above) and the VSR activity *per se* may result in suppressing the effect of exogenous dsRNA by converting it into single-stranded RNA or by blocking incorporation of already-formed 21–23 nucleotide-long siRNAs into AGO complexes.

If our hypothesis on the adaptive role of SF1H VSR for herbivores is true, one can expect that the phytophagous insects outside Lepidoptera may code and express this functional domain. It is known that, in addition to Lepidoptera containing almost exclusively herbivorous species, seven more insect orders are represented by a substantial amount of herbivores (Wiens, Lapoint & Whiteman, 2015). Among these orders (Diptera, Coleoptera, Hemiptera, Thysanoptera, Hymenoptera, Phasmatodea and Orthoptera), hemipteran species, like lepidopteran insects, are more refractory to dsRNA-induced silencing (Swevers, Van den Broeck & Smagghe, 2013; Wybouw et al., 2016). In support of our hypothesis, we found that at least five representatives of this insect order express virus-like SF1H-coding transcripts during their ontogenesis (see above).

## Conclusions

Considering all possible hypotheses on the functional significance of acquisition of virus SF1H coding sequences by insect genomes, namely, (i) providing selective advantages to retrotransposons as selfish genetic elements; (ii) using the SF1H coding sequences as a tool for anti-viral defense of insects; (iii) active expression of virus-like SF1H VSR as a possible prerequisite for herbivory, one can consider these scenarios as mutually exclusive. We prefer a more complex view on the acquisition and preservation of functional SF1H coding sequences in retrotransposons of many present-day insects. As suggested previously, it is possible that basal insect groups together with sister invertebrates represented a major reservoir of viral genetic diversity for potentially billions of years and, thus, have been central to RNA virus evolution (Li et al., 2015; Dudas & Obbard, 2015; Shi et al., 2016). Accordingly, anti-viral defense likely was very important for survival and natural selection in the course of insect evolution (Palatini et al., 2017), that can explain the preservation of expressed SF1H in insect genomes. As an independent scenario of initial evolutionary fixation of SF1H in insect genomes, we consider that acquired virus SF1H coding sequences could provide selective advantages to retrotransposons as selfish genetic elements. Later in the insect evolution, irrespective of initial scenario of SF1H fixation in insect genomes, the acquired SF1H-based machinery for silencing suppression could work in favor of emergence of herbivory and herbivorous lifestyle expansion.

##  Supplemental Information

10.7717/peerj.3673/supp-1 Supplemental Information 1 Amino acid sequence alignment of the SF1H proteins of positive-stranded RNA viruses and analyzed SF1H proteins encoded by Lepidoptera TRAS-like LINEs of Lepidoptera

10.7717/peerj.3673/supp-2 Supplemental Information 2 Nucleotide sequences of genomic RNAs of six analyzed invertebrate viruses

10.7717/peerj.3673/supp-3 Supplemental Information 3 Multiple sequence alignment of the analyzed SF1H proteins encoded by Lepidoptera TRAS-like LINEs and Jockey-like LINEs of three other insect orders

10.7717/peerj.3673/supp-4 Supplemental Information 4 Nucleotide sequences of genomic loci of four analyzed retrotransposons

10.7717/peerj.3673/supp-5 Supplemental Information 5 Amino acid sequence alignment of the SF1H conserved domains encoded by insect LINE transposons

## Additional Information and Declarations

### Data Availability

The following information was supplied regarding data availability: The raw data is included in the figures in the manuscript.
