# Supplementary material for: RNA helicase domains of viral origin in proteins of insect retrotransposons: possible source for evolutionary advantages
Source: PeerJ. 2017 Aug 16;5:e3673. doi: 10.7717/peerj.3673 (PMC5563155; doi:10.7717/peerj.3673)
Supplement: Supplemental Information 1 [file peerj-05-3673-s001.doc]

**Amino acid sequence alignment of the SF1H proteins of positive-stranded RNA viruses and analyzed SF1H proteins encoded by Lepidoptera TRAS-like LINEs of Lepidoptera**

Andesiana_lamellata IRWVNGVPGCGKTTWVVKHFDE-------EKDVVATTTTEAAKDLREKLAHRLG-----DRVKTKVRTMASILVNGFK-K--QEKCYRLTVDEALMNHFG

Biston_suppressaria MQWVNGVPGCGKTTWVIRHFEV-------DKDLIITTTTEAAKDLREKLSHRIG-----NLVKTRVRTMASALVNGFG-K---EGFSRLMVDEALMNHFG

Broad_bean_necrosis_virus ITLIDGVPGCGKSTYIVNNAD-------VRTDLILSMGKEATEDLKRRFTKEKGARQ---EDMKRVRTVDSYLLNDFG--N-KLRADTVHFDEALMAHAG

Caloptilia_triadicae FTWINGVPGCGKTTWVIDNIDY-------KDDAVITTTLEAASDLTNRLAGRIG-----AVSKSLVQTMASVLVNGLK-A---PRRKRLFVDEALMNHFG

Eudarcia_simulatricella INWVNGVPGCGKTTWVIGHFEA-------ATDVVITTTTEAAKDLRERLALRLG-----ADATSKVRTMASMLVHGLR-GRDKDKCKRLIIDEALMNHFG

Hubei_virga-like_virus_1 IEWTNGPPGCGKTYHIIQNAHIPISTTQEPEDLVLCMTTEGRRDVMAKMKSKRPEMSD-EVLQRDVRTVASVLVNGCA-----VAYQRVLLDEALMAHAG

Hubei_virga-like_virus_12 IYYVNGVAGCGKTQNIISNFDI-------NDDFIISTTKANTEELIERISKHYKIKKE--IIISRVKTIDSVLINKPR-----QNIKKLFIDEIFMVHAG

Hubei_virga-like_virus_2 IEWINGPPGCGKTHAIVHSANVSIDP-LVGRDLILSMTSEGKTSIRAGLKKRLPSLTD-RALQAHVRTVASLLVNGSA-----VKYDRVLMDEALMAHAG

Hubei_virga-like_virus_21 IALRNGVPGCGKTKYIIDNAE--------QADYILTTTRENKQDIVSRCP----------TMRSRVRTVHSVIINSKT-V-ENTSVRRLFIDEALMSHAG

Lodeiro_virus YRFIQGVPGCGKTTWIVQNFK-------PGSSLVLVSTVNGRDDVIGRLKEEYGGE-----HNRNVQTYASILMNGPK----QEGIEWVICDEAGMQHPG

Lyssa_zampa FTWINGVPGCGKTTWLVSRFDA-------NEDVVVTTTTEAAKDLLEKLTRRIG-----KAAKSKVRTLASVLVNGFR-EPDKRRCNRLMVDEALMNHFG

Ostrinia_nubilalis ITWVNGVPGCGKTTWVMSQIDT-------SRDIIVTTTCEAAKDLREKLEPKIG-----ARAKKRVRTMASLLVNGMS-E--GETCTRIMVDEALMNHFG

Paprika_mild_mottle_virus VTLVDGVPGCGKTKEILRRVN-------FDEDLVLVPGKEAAAMIRKRAN-QCGNTV---ANNNNVRTVDSFLMNLGK-SP-RNHFKRLFIDEGLMLHPG

Plutella_xylostella IAWVNGVPGCGKTTWVVNNFDV-------SKDTIITTTTEAAVDIRNRLAHRIG-----DMVRTRVRTMASVLVNGFR-E--HVGCQRLIIDEALMNHFG

Polyommatus_icarus YSWVNGVPGCGKTTWIINNFNE-------ETDVIITTTIEAAEDLKQRLSLRTG-----NKVKDKVRTMASLLVNGTK-----GAYKRLIVDEALMNHFG

Potato_mop-top_virus ITLVDGVPGCGKSTYVVKEAN-------LVNQYVVTIGREAAEDLRERFKSERNATA---TQLKRVRTVDSYLLNDTQ-----SRANVLHFDEALMAHAG

Sindbis_virus TIGVIGTPGSGKSAII-KSTVT-------ARDLVTSGKKENCREIEADVLRLRG-----MQ--ITSKTVDSVMLNGCH-----KAVEVLYVDEAFACHAG

Soil-borne_cereal_mosaic_virus ITLRDGVPGCGKSTWIVENAN-------PMKDMVLSMCKEATEDLKEKFAKRLRCTE---SALRRVRTVDSFLMHDYD----KFRAATVHFDEALMAHAG

Soil-borne_wheat_mosaic_virus ITLRDGVPGCGKSTWILNNAN-------PMKDMVLCVGKEATEDLKEKFMKKHKCTE---SDLKRIRTVDSFLMHDYD----KFRAATVHFDEALMAHAG

Streptocarpus_flower_break_virus IILVDGVPGCGKTKEILERCD-------FTKDLILVPGKEASKMIIKRAN-AGGKNR---ANQDNVRTVDSFLIHMK--G---TQVKRLFIDEGLMLHTG

Tischeria_quercitella IYWINGVPGGGKTRWIITQFKV-------GTDVIITSTTQSAADLKEKLSQRVG-----PNARSSVRTMASILVNGLR-G--QGTCRRLIIDEALMNHFG

Xingshan_nematode_virus_2 ITLVEGVPGCGKSTYILRNHKFSV---DDVKHVVLTATKETAEDMRRRAAEMYGVSSELSILRKRYRTVDSFLVHCGGMLEEDCVVETLWIDEGLMKHFG

Xinzhou_nematode_virus_1 INLVEGVPGCGKTTYIVNNHKFAI---DDLSDVVLTATRETAEDIRKRVCVAYSVSEDLPILKKRYRTIDSFLVNFSS--K-DVGINTLWIDEGLMKHFG

Andesiana_lamellata TIVMAVKLS-GASEIVLIGDVNQLPFLDRENLFKLRYTRPNL-VAGI--TQELHCTHRNPMDVAFALSE--IYS--GI-YSSKSSLSRVHSLKVKGYTG-

Biston_suppressaria AIVMVSRLS-GAGEIVLIGDVNQLPYIDRENLFEMRYHRPNL-VTKI--SQELLCTHRNPMDVAYALRE--IYS--GM-YSSVC---RIKSLEQKRYKG-

Broad_bean_necrosis_virus MVYFIAMMC-SAKRIKCQGDSKQIPFINRVESIKLEYA--KL-DIHE--TIAKRLTYRSPLDVAYYLTKKGFYG--LDFITSANP--LLRSMKTVGPRSS

Caloptilia_triadicae AIVMASKLA-KVEEVVLIGDVNQLPFIERENLFPVTYHRP-L-HFGI--EKDLLCTHRNPMVVAFALRK--IYG--GM-YSPKR---QVRSLAKKGYRR-

Eudarcia_simulatricella AVVMAARLA-GASEVTLIGDKNQLPYIDRENLFTMKYDRPNL-VAQI--TRELLCTHRNPMDVAYALSE--VYN--GI-YSTSP---KVKSLTLKTYKD-

Hubei_virga-like_virus_1 TIGFVVYYS-QAKAIAMIGDIHQIPYVDREHMCSVLYHVPSR-FADI--TKSLNRTYRCPVDVTYALSA--FYE--GL-HTTNG---IILSVRQLTYSGN

Hubei_virga-like_virus_12 TIMFAIQIL-KPKYVLALGDTKQIPYIERLALIPNNYSSIID-ISVK--LENLNVSSRCPADVVEIFKN--DYKDSGGFYTTN-G--RTRSVRIQNFISK

Hubei_virga-like_virus_2 TIGFAVALT-GTKKVLIIGDIHQIPYVDREHMCKLQYETPAV-FADV--TSVKEITYRCPMDVTYAISD--LYP--NL-CTMSL---VTVSVNQKPWSSN

Hubei_virga-like_virus_21 ELLIAITIL-RPESVEMSGDVNQIPFINRAAAIIMKFDDAAR-ICDS--ITHASVSYRVPKDVAALFSS--SYE--QG-FTTNNK--IESSMKWVEVTGY

Lodeiro_virus AIDFSIKMT-QCQKVTVLGDGNQIAFIDRHHF-NIVHGDLLE-ILKA--DEHLSVSWRVPQDIASYFSD--QYP--GG-FMTRNK--VRKSVNWVKIQSL

Lyssa_zampa AIVMAARLA-EAKEVLLIGDINQLPYIDRENLFPLFYYRPTQ-LTTI--SQNLLCTHRNPMDVAYALRE--VYD--GI-YSSVM---CVNSLKRGMYKG-

Ostrinia_nubilalis SIVMAVQIA-QASEALLIGDNNQLPYIDRNNLFPLLYNRPNL-ITNV--TKELLCTYRNPQDVAYALRE--IYS--GI-YSAKT---LTRSLQLKGFTG-

Paprika_mild_mottle_virus CVYFLVTLS-LCDEAFVFGDTQQIPYINRVQNFPFPKHFSKL-IVDE--TEKRRTTLRCPVDVTHFLNQ--RYD--GA-VTTTSK--TTRSVGVDVVNGA

Plutella_xylostella AIVIAARLS-RASDIALIGDINQLPYIDRENLFELRYSRPTL-VANI--TQELLCSYRNPMDVAYALRE--VYS--GI-YAATT---RIQSLQLKRFTD-

Polyommatus_icarus SIVMANQLT-GANDVILIGDINQLPFIERENLFKLNYTRPNL-VTGI--TQELSCTHRSPMDVAYALSM--VYN--NI-YSSKE---IVRSLKLTKYTG-

Potato_mop-top_virus MVYFCADDL-SARSVICQGDSQQIPFINRVESITLRYS--KL-EIDN--VVEKRLTYRSPLDVASYLTKKNFYG--TSVVTSANP--LVRSLKTVGPRDG

Sindbis_virus ALLALIAIVRPRKKVVLCGDPMQCGFFNMMQL-KVHFNHPEKDICTK--TFYKYISRRCTQPVTAIVSTL-HYD--GK-MKTTNP--CKKNIEID-ITGA

Soil-borne_cereal_mosaic_virus MVYFCADIL-GAKRVLCQGDSQQIPFINRVESITLKYA--KL-QIDD--TEYVRLTYRSPIDVAHYLTKKSYYS--GGRVLTKNT--TLRSMNTVGPRDA

Soil-borne_wheat_mosaic_virus IVYFCADIL-GAKKVICQGDSQQIPFINRVESITLQYA--KL-AIDE--TEYVRLTYRSPVDVAHYLTKKSWYS--GGRVTTKNS--VLRSMKVVGPRDA

Streptocarpus_flower_break_virus CVNFLALFS-HCEEVLVYGDTHQIPFINRVANFPYPSHFAQL-QYDS--VEKRRVTLRCPADVTHHLNS--QYD--GK-VMCTSS--ILRSVECEVVRGK

Tischeria_quercitella SIVMIVRIL-QAEELLLIGDVNQLPFIDRDNLFKIKYHRLPI-TPHK--HQELHCTHRNPVDVAYALQN--IYD--GI-YSSST---TIKSLNITNFTG-

Xingshan_nematode_virus_2 EIMWCVYLS-KARQVFICGDRAQIPFINRNGSVKLYYSKIDL-VLDSIKVKFLDKSYRCPADVVAHLNALNVYP--GK-VTTENT--VVYSIKVRNITGL

Xinzhou_nematode_virus_1 EIMWCVYLS-GAKNVRICGDRAQIPFINRNGSISLLYSKMDA-LTKRFSVEFLQNSYRCPADVVCYLNSLGTYP--GK-VSTINK--TMRSIHVQIVTGI

Andesiana_lamellata --A-QI-PS-TAQNTLFLVHTQEEKASLISQ---GY-GSG-EGSRTLTIHEAQGLTYDSVIIINTKSRR-LQIH----DSISHAVVAVSRHTVSCVYYSD

Biston_suppressaria --A-QI-PN-TLPNTLFLVHTQEEKETLTNQ---GY-GSG-TGSRILTIHEAQGLTYESVIVIKTKA-N-MKLH----ESVPHAVVAISRHTGNFTYYAD

Broad_bean_necrosis_virus TPMSSIYVIPKTKGASYLTFTQTEKDEMKQ----YL-GSG-D-WTVNTVHEAQGKTFNDVILVRLKNTEN-EIYPGGRNSEPYMVTGISRHKRSLIYYTR

Caloptilia_triadicae --S-TV-PE-DLPNTLYLVHTQAEKAALLSL---GY-EKG-EYSRLLTIHEAQGLTYEDVVILNTVEKK-MRIH----DSVSHAVVAISRHTRSCTYYTD

Eudarcia_simulatricella --A-HI-PA-DSPNTLFLTHTQAEKELLKSE---GF-GSG-DKSRILTIHEAQGLTYESVVVIRVADRR-TQLH----DSVPHAVVAVSRHTLRCVYYTN

Hubei_virga-like_virus_1 V-T-HI-DK-ACTNTLFLVHLQADKDALVSE---GY-GKA-SGSAVLTVHEAQGLTFNHVICIRRNSKP-LEIF----SSLPYAIVAISRHRESFVYYTD

Hubei_virga-like_virus_12 D---NL-KSLIDEETMVICFKEADAEELILY---G--------IDAYTIHKKQGTTKKKVILVRLSVKPNEEIF----KKREQILVALTRHTEQFIYYTK

Hubei_virga-like_virus_2 N-T-HV-TK-TVEGCLYLTHTQPDKDALIKA---GY-GKG-AGSAVMTIHEAQGLTYAHVVCIRSQPKA-LAIY----SRSEYALVAISRHTKSFVYYTD

Hubei_virga-like_virus_21 N---EL-PK-A---DPVLVFKQAEKAMLRLE---GY--------DVSTVHEYQGKQSQKISLYRHSTIPSDQIY----MSDPHILVALSRHTQSLVYYTR

Lodeiro_virus K------SL-DWDHDVYLTFTQQEKTEVLIE---GK-GKK-ENIKVRTIHEYQGDQARSVAIVRNRDKDVNRIY----ESDEHILVALTRHTQKLVYYSA

Lyssa_zampa --V-NI-PK-TLSETLYLVYTQDEKISLTNQ---GY-GSG-EGSRLLTIHEAQGLTYKRVIIVNTMDKK-LQLH----DSVAHAVVAISRHTIECVYHAD

Ostrinia_nubilalis --A-KI-PN--QEDTLYLVHTQAEKALLIGQ---GY-GTK-TGSRTLTIHEAQGLTFREVVIVRTTSKK-SHLL----QSVPHAVVAISRHTDSCTYYTD

Paprika_mild_mottle_virus A---TMNPVTRPLKGKIITFTQSDKITLASR---GY-------ENVNTVHEIQGETYDDVCLVRLTPTPI-HIIA---RDSPHVLVGLTRHTKTFKYYTV

Plutella_xylostella --A-AI-PK-SQTNTLFLTHTQEEKETLTSQ---GF-GEG-TGSRVLTIHEAQGLTYESVIIIKTKD-K-IKLH----DSIPHAVVALSRHTSACTYYAD

Polyommatus_icarus --A-RI-PK-TDLNTLYLVHTQEEKAALTNT---GY-GSG-TDSRVLTIHEAQGLTSPSVIIIQTKSRK-LAIH----DSVPHAVVAISRHTNTCVYYTD

Potato_mop-top_virus --MTSIYSIPKIPGTQYLTFLQSEKEEMRQ----YL-GRG-N-WNVNTVHESQGKTYDNVVLCRLKATDN-EIYPGGRNSSPYMVVGVTRHRRSLVYYTK

Sindbis_virus T----K-PK---PGDIILTCFRGWVKQLQI-D-------Y-PGHEVMTAAASQGLTRKGVYAVRQKVNEN-PLYA---ITSEHVNVLLTRTEDRLVWKTL

Soil-borne_cereal_mosaic_virus KPMTSVHCVPYHRDTQYLTFTQSEKADLYKA---LR-NRG-P-VNVNTVHETQGKTFDDVIVVRLKTTEN-EIYPGGRKAQPYEIVATTRHRRSLVYYTA

Soil-borne_wheat_mosaic_virus KPMTSVHCVPYHRDAQYLTFTQSEKADLYKA---LR-AKG-P-VEVNTVHETQGKTFDDVIVVRLKTTEN-EIYPGGRKGQPYEIVATTRHRRSLVYYTA

Streptocarpus_flower_break_virus A---VLNPKTKPLSGKIITFTQSDKLELQNK---GY-GEV-DVLDVNTVHEIQGETYEHVSLVRLTPTPL-EIVS---HGSPHVLVALTRHTQSLKYYTV

Tischeria_quercitella --A-NI-SR-TQENTLYLFHTQAEKESFKAE---GY-ASG-EGSLILTIHEAQGLSYKTVYIIKSLRKH-TQIH----NSVSHAVVAISRHTESCTYFTD

Xingshan_nematode_virus_2 V---DV-PFTDWKDATVLTFTQQEKTDVILHVGKFF-SKGLFDGKVFTVHEYQGKQTKRILLIRLQVKPI-SIY----DSVSHQLVAITRHTREFMYCTV

Xinzhou_nematode_virus_1 A---DV-PFLDFKRAVILTYTQREKQEVTLHLNKIFPGKD-VSYTVNTIHEYQGKQAADVVLIRLQMKEI-TIY----NSVSHQLVALTRHTHSFTYYTV
