# Supplementary material for: RNA helicase domains of viral origin in proteins of insect retrotransposons: possible source for evolutionary advantages
Source: PeerJ. 2017 Aug 16;5:e3673. doi: 10.7717/peerj.3673 (PMC5563155; doi:10.7717/peerj.3673)
Supplement: Supplemental Information 2 [file peerj-05-3673-s002.doc]

**Nucleotide sequences of genomic RNAs of six analyzed invertebrate viruses**

**Hubei virga-like virus 2**

**KX883772**

1 tggtgatcgt tttgttgaca attgccaagc cggcggtagc tgtattgtta ctctatcgct

61 tcctactgtt ctcgtgacgc cacgcgaaca accatgccga cgtttccgaa ccaacgcaaa

121 accctggcat ctgccaagcc gtcgaggacg ccccgagacg gtactaaagt cacggagaag

181 cgcgggtatt gttacctcgc gctctttgag gcttttaacg cttcgtctga gaagaagtta

241 gacgttgcgt caatcaaggc tcgtttggga gcctttcccc tcgtgagacg cgttgttggg

301 gagttgtacg ctcacgtgac atttgacttg tttgttcctt gcgtgcgcag ggtaagcaat

361 acgatgttcc acgtggacga atggcgcccc ccaatgttgt tctctgaggt actcgcgatg

421 acgatctttt cgagtgcgag aattggtgcg gatgaccgtg cccacttgca gcagcaacag

481 ctgatcaggg tgcaggactt atgtaagact gcaggcctgg accgcaacac cgtccttgct

541 gatgctgcta aaaagtgtct ctcccgtgcg cttgctgcgc cggactccga aatgaacgcc

601 attttcgagt ctttcctgcc caagtccaag tcgggctttg tcagcccgta ctatttggac

661 gtccaacagc tcgcctcgtt gcaggcggac taccccgaac tcggtgtgtc cggaggtgga

721 acgatccatc atcctcacgc ctacgctgct gtcgctacgc tgtgtcaaga agccatcata

781 ctcaagcgta tgaactatgg atcaaccacg gttcctccgc gaggttatga tgcctacgcc

841 atcgatgttg gtgctaacta cccgcggcat gctaaggctg gacgatttga cgtccacaat

901 tgctgcccca tcatcaccgg gcgtgactca gctcgcgaca ccacgcgcgc gcttgttatg

961 gcgtcccttg tctctggagg caagttgact gctgaaacgg cgcttaaata tactgacccc

1021 gcatcggtcc ccggaaaaaa cctgctgaga tgtgcgaacc gtgctgaaca gtgtactgtg

1081 accgctcctt acatgatgtt cgttcattct cagtacgaca tcaccctgga gcaactcggt

1141 gctatgttcg acgctcatcg caccatcaaa gcgtacagcg tgatgaattt tgtgcctgac

1201 attttcgtcg cttcgcaggg aaccgttgcg tcactcggat tgacctacca caaagaaggt

1261 gatgacgtag tcttctcttt cgtcggcgac tcgtccatga attatcgtca taatctcaac

1321 acgctgctca ggacctacac ggcgcaacat atcatttctc cctcgggcgt gatgtatgtg

1381 tgcgaacgtg ttgttcgtgg aggtgctcta ttcgtggagt acaccaggtg cactatggcg

1441 cctgagctgc aggaggagta ttttgacttt aatttctggg acgccgccgc ggaagacgtt

1501 gtctatctga ctacctggca gtacgaccac acgaggtatg ctggaagtaa gatggcggtt

1561 aagggccctg ccgggttcca tagactgatc atcggtgtgg accgcgcatt ctttgagcgt

1621 gtgtacgttc actgtttgcg atccggcgat acttcgttca ttatcaatca tgtggtctct

1681 gccgcttcca ctttcaacac caagatgagt atcaacggcg ctaacatccg cgctgtcaat

1741 cgtgtgggca tggatgtgtt ctttgacgcg gtgattgcga tatacatgat cgcgtatcgc

1801 acccgttgga tgggaacaaa gaccctgcat cacatgatcg agtcagagaa gggcgtgcgc

1861 ggcgattata agatcgggtt catcggcgct tttaaggcgc tgtggaaaat cctctcgtcg

1921 ttcaccactg gcaagctctt cgaatcgatt gctgaggtgt ggaacaagtt tgtcagcaag

1981 tgcggcgagc ttaattctgc tggcgactta gatgtcgagc tgctgcaatc cgttcgccag

2041 atccacttct cggatttgct gcggtacaac gcgaagtacg aaggagcgta cccggccctt

2101 tacgacatac gcgtggacgc gattaagttc gatgtcaagg acggtatgtt gcaggcgata

2161 tccgaggtag tgcgatccaa gtactcgagc gaccctgccg atccgccgaa gcggcccgca

2221 cacgtggacg gtcagcacgg caacgaccat aaggaccccg gcaacgtaac tggcaatact

2281 gacactccga aggagagtcc acaacccgga ctgccccccg cgccaagtgc gccaagagtc

2341 gtaggcacac ctgccgcccc gatgagtaac aagacccgaa acggtgtgat aacggagttt

2401 caccgcaccc agtccataaa gtctgagaag aacagtagca gactgcgcgc tgggcggcac

2461 gaggcgtacg cctgcacttc ggtgctcgtc gtgaagccga cgcccccgga tggaaactgc

2521 ctattcacgt ctttgggcta tttcgtcaat ttgaccgcca acgaaatgcg cgctgatttg

2581 tgtggcgctg tcggagacat ggaattgcga tacaatgatc gcggaaggag tacttgggga

2641 aacacagact gcgtcacgac gttttcgtct ctgtaccaga agcgtgtgtg tgtacacatg

2701 actgttactg acaagaagac gtaccccact gtcacttctg aaatgtattc cgcagaagga

2761 accacggaca tggtgcattt ggattggcgt gtcgttggtg acccgaagac gaataatcac

2821 acgggtcatg tcgaagtact tacgcagcct aacgacaagc gcgaactgcc gcgtgaagcc

2881 ccgacatgga agagcgtgtc gcacctattt gacaccacgc gacgccttcc ccacggatcc

2941 accgatgagt accacggatt tccgcgtcac caggagctca aagctttgag cctgctcgga

3001 agtgcggatg ccgctaccac tcgccctgct tccatcctgg aacttggtgc ggcgccgggt

3061 acttggacta aactgctgct gacctacgcc agcgcccgca gggccgctat cgacgttgtc

3121 agcgcaccca ctggcttaga gatggctgaa gaggtgttgg cagaaatcga atccgacgac

3181 gcatacactt tgcaccaaca ggacgcagtc acgttcttga cggagaccgc caaaaagtac

3241 gatttcgtcc tttctgacgt cgccactgcg gacagttggg ctactaattc accgcaactc

3301 gacgtgctat gcgctgtcgt gtcgcggttg aatttgggag cgacgttggt gttgaagttg

3361 tctaacgtgt ttctggataa ctccctggat gctatcgcga attgtcgccg actattcgct

3421 tctgttgaag tggtcaaacc ggctggatca cgcctgcgta acactgaggt ctatctcgtt

3481 tgcaaggcct acggtgaacc agcgggacga accaacccct actctcagag gcgtctcata

3541 atcgcagaca ttctcgcgca cctagaaaga ctgaaagcag gtgaaacggt agtgcgtgac

3601 gttgtcgacg agtattctcg cttgagcttt gcgttcatcg agtatactag agacgtgacg

3661 atggaatggg acgaccccag tgacggtgag gaagaatcgc aggctgaccc ggtaccccct

3721 gctacgcctg cgttgacccc ggtgcaggag ctatacacgt tgactttcgt ggaagctcag

3781 attgccgctg cggctagtcg acttgcggag gatactggtc gcccagcatc ttcagcctgc

3841 gcgcgcccgg ctactcccac accgagttgc gctccgtcag actttagcac gaagagtgcc

3901 cctgctgcta taccgactcc cgattcgccc aaactgaacc cgcccagcac catgccgaac

3961 gctgctggga gttcaccacc ggaaccgaca cctgcccgta gctctcctac atctagctgc

4021 gcgtcctcgg acgccggcgt ggagactacc tgtccaccca ggccgaaccc gccctgcacc

4081 ttgccgactg ctgcagggag ttcaggtccc gaaacgacac ccgcccgcgg atccgccgaa

4141 cgtcccacac gctctaagcg acctataccg tctttcgtgt tcaccccttc cgtcaagacc

4201 tcgctcgaat cgcagccttc cctcctctcc attggagaga acgcgaaggt tgaggcttcg

4261 actactctcg gcgcaaacgc atctcccgct ctatcctcca ctttcctcgc tgaaaatggt

4321 ttggttatga acttggacga tgcattgttt gacgccgaac acccgtggca gagcgcagct

4381 caccacatgc aggtctacca caagggcagc ggtctctacg aacgcataat ggagttgggg

4441 gtatactctg accggctgca ttacgaatac gacgagataa tgtcgctatt gggacccacg

4501 gcgtgcgatt tcgcgttacc tgaaccagtc gctctcctcg tccaacccga gggcgcagcg

4561 tgcttgacta aggcacacag tgcgagcaga ctggcggacg gaaagacggt ggctgttcgt

4621 gtgtctgacc cgggtgttcc acacctcacc gcgcctggct ggcacgtgca agtacatcga

4681 tgtcgttcta ccccgcccac cgacgtggct gtgtattaca tctactctca actaccctct

4741 cgggtggtcg cgggcagttg gaagcacact atgtcgcaat gcctcaacgc agcggtaaaa

4801 ggagttgccc cgtacgatcc cgctctcctg agagctgtct tgcgtacgac tgctgccggg

4861 ggttgtctgt cggatctaga agcggtatcg aagtgcgatg cgcatactga acttgacaag

4921 gcgtgcttgt gttgcgtcta cgtgacctct ggtgatattc aaccaccgat ggttgcgtct

4981 tggatgcgtc gcaagtacaa ccaaattgcg gctcgtgcga aacgtattta tctggagaat

5041 ggtggtgccg aagccctatc tctggatgag gctttaccag tttacatgca gcgcgaacgt

5101 agagatattt ctgcgtcggg tttgcgtgtg gacgggtcag ccattacgtt gaagtcctac

5161 gagccgaaca agtttatacc tgcttttaca accggcggtc aggtgattaa cgccatgaaa

5221 ccgggaacgc acaaagtgtc tgccggggat caagttgcac acgtgaccac ttacggtgct

5281 gtcgcaaagc aaacagtgga cttctggggt tcgaagggac gaacacgcgc tgcggtgacg

5341 gacgctaaag gaagacacac cacgctatct gaggtagtgg gtacttccag ccgcgtcact

5401 tctaccgtga cgaatgttgc ttctctccct gctccggaaa accgcactgt ccgtgatgtg

5461 gcggtcagcg tatccgatgc taaattcggc tgcgtgtctg tgcatgctag tatcccacgt

5521 gcagcgaatg tcgtcgtgtg ccagaagctg atcacgtaca agactgccgg aacgcagact

5581 gccgagacgg gctctgcagc ttacaccatc tgtgcgaaga aaacgtcgcc ggacttgccg

5641 agtgcgtcgg agagcgacac cactacgctt accagtacaa cctcgacctc agtgtcgata

5701 actaagcgcg gtgatgactt tacctccacg acgtcgacac cgactggcat ctcccagttc

5761 accgagactg gtccggttgc ttttaccgcc gccctcaaag cacgatctga agctgtccaa

5821 cgcgccaagc gcgaggccga gaccttccaa ccactggcta cttcctcgac tattagcgaa

5881 tcggacgcgt ccaagtcggt atctacgacc ggtacgggcg ttactgtacc cttcgcgtct

5941 ggggggaaac cggctagcga cacttccaac tcttgggcac cttcctcgac ggtcagtagt

6001 tcgagcacgt ccacttcggc ctccacggtc ggtgtgggcg cgtccacgtc ctcctcgtcc

6061 gggaagaagc acgcgagtag gactgcgtcg ctaatggcaa agttggggct cgacgacgct

6121 tcctcagcgt cgaccgaggt cgcatctgcc ggtcaggcca gtacgaacgc gttgttacct

6181 gacaagacta aacgttccaa gctgcaagcg tgtaaatcgg cgtttaaggc tgtcatgcaa

6241 ccgtcaaagg cggctactcc gcgtcaacct gccgcggagg tctgtcagtc actgcctcgc

6301 ggttgcaaag atacgttggc cgtcggtgga acttggactg gttatccgca cgattgcaag

6361 cttgttttgc acttgctcct ttcgtgtggt aaagagttcc ttgccactga cagtcttgcc

6421 aaacgcttga agaaggaacg aattaccgaa tgtgatgtgg cgagacgcgg gtattcacgt

6481 ctggagcata cgctggagct gttctaccac catgatggtg ttcacgattt cgttctcgtg

6541 acaccagctg tcggtgcaga aggggtcctg aagcggttcc tgtcgtggct gcctgaagac

6601 gtttctcgag ttgcgatcga aagctctgcg gacctagcgg atagtgcgtt gacggagctc

6661 ctacgtgagt gtgacagacc ggtaaccctt gtcgacggtc gcacgcgtag gtgggctggc

6721 gtcctacacc ttctggagcc gtccaagcga ttgcttcctc cggtgagacg tcccctggct

6781 ttccctaagc tctcccaggt tgagatgggc tacataccca atccaccgtg gatcgcagct

6841 aacaccaact tggacaggta ccggaacgcc atgatcgagt accgctacac tactgctcat

6901 gctgaccagt tcaataaact gcacttccag aaattacaac tggattcaaa aaacgggata

6961 cccgcgggaa agacggccga gattctcgca gcgggatacg gtgtgtacga ccagcagaca

7021 aaagcctatc tgggacagaa agcgtcgcgc acgtattcgc acgggtattc ggtcagcaag

7081 caggagtacg ttgcatggaa cgagaccgcg cgacagttca acagtcccga ccggtttatt

7141 ctggttggga ggaacaccga actgatgttg aactcgcaga tctcggcgca gattgctgga

7201 gtggatgtgg cgagtcagga actaccggaa atcgagtgga taaacggccc gcccgggtgc

7261 ggcaaaaccc acgcgatagt acactcagcc aacgtctcta tcgatccact ggtaggtcgc

7321 gacttgatct tgagcatgac atccgagggc aagacctcga tacgagccgg actgaagaaa

7381 cggctgcctt ccttgacgga cagagcgcta caggcgcacg tgcgtacagt cgcgtctctg

7441 ttagtgaatg ggtcggccgt aaaatacgat cgtgtactga tggacgaagc cctgatggcg

7501 cacgcgggta ccataggctt cgcagttgcc ttaactggta cgaagaaggt attgataata

7561 ggggacatac atcagattcc ctatgttgat cgcgaacaca tgtgcaagct ccagtacgag

7621 acgcccgccg tattcgctga cgttacgtca gtgaaagaaa taacgtaccg gtgtcccatg

7681 gatgtgactt acgcgatctc ggatctctac ccgaacctgt gtaccatgag tcttgtgact

7741 gtgtctgtca accagaagcc ttggtcatcg aacaacacac acgtcaccaa gacggttgag

7801 ggatgcctgt accttacgca cactcaaccg gacaaggacg cactgatcaa ggcgggttat

7861 ggaaagggag cgggttccgc ggttatgacg atccacgagg cccaaggcct aacgtatgcg

7921 cacgtcgttt gcatacgcag ccagccgaag gccctcgcga tttactcgcg ttccgagtac

7981 gccctggttg ctatttctag gcacaccaag agctttgtct attacaccga tgttgacgac

8041 gcgttgacga aactcatacg caaagcgcag tcgaaagacc cggctgccct gcagatatgg

8101 aatgctccgc gactgactgc cgcaaagaag aagttgactc cgctcactgc gggtgggttg

8161 ctctgggagg ccgacaccgt gttgtcccca ctcgccaatc ccactactgg ggattacatg

8221 gcgggtttgc ccgaagtgtc gcatgcccct ctaatgccac tcgctcccga acgaattgcg

8281 gaatcctcct tcagaaagtt gccgaagttc aggcccaacg ttgacacgga cgttgcattt

8341 ctgcagactt tttacgacga cgtcatggct gaggcgataa ccgttgagta caagtacgat

8401 caaatgatga tggaaatgga ggacacgtac cttgtgtcta ctcccatcac agttgaccca

8461 ctgctgggtt tacctgctga tcgtaagtac ggtaagttgc gcccactgct taggactaac

8521 atgcgcgcgg aaaagcaacc ctcgcaaaag gagtcattac taggtgcgat aaaacgcaat

8581 ctgaacgctc ccctgttgcg taacgccgcc ctgtccgaag aagcaattgg agaactgctc

8641 tttaagaact ttgagcggtc ggccatagac gagacgaaag tcgccatcta cgagtcgtat

8701 gccgatgatc cgatcaacat caactctcag gttgtatctg tctggctgga aaaacagcct

8761 ccctcccgca ggaagcagat tgtgtcggac ttgccgttac acttgcgtcc cttcaacaag

8821 ttcgagttca tggtaaagaa agacgtcaag ccacagttga cgcctgactc catgtattcg

8881 taccccagcg tgcagaccat cgtttacaac gatgcctccg ttaacgctgt gagctgtccg

8941 atttacaact tgttgtggga acgtttgttg gcggtactgg atcctcgtgt gttggtgatg

9001 actgggatgt ctccggttga gttcgaggcc gagtttaatg ctaggctgtg cccggaagtc

9061 gcgtcggtgc tgcgtacgct ggagaatgat ttcagcaagt acgataaggc gcaggctggc

9121 gccctacgtc gactggaaca cttggtttgg acgaagctcg gactagatcc ggagatcgct

9181 ctaatttggg acaggtcccg tcgaacaagc gacgtgaggg acaggaagaa cggagtcagt

9241 tttgtgacgg agtaccaacg taagtccggt gaggcgacca cattcagcgg taacacatta

9301 gtcgctatgt gcgtgatgct ggctgtgata gacatcgacg acattgtgtt gatgttagct

9361 gccggcgacg acaccctcat ctacttacgc cccggaactg acttctctga ttcttctgct

9421 ctggtcgccg atctgttcaa cttggagtgc aagctcttgg agtgttacga agtaccatac

9481 ttctgctcta agttcttgat ctctacaccc gattggacgt acttcgttca cgatctgctg

9541 aagttcgtga cgaagttggg gagacacgac atgtcgaatt acactcacgt ggagaactac

9601 cgtgtgtctt gtgtcgacac gatgagatcg ttgttcaacc ctgtggtcgc tcccggactg

9661 actgtcggaa tgcaggaacg ttatcacagt actttctgtg atgtgacgaa agtgttagcc

9721 gtacttcgta ctctttgtca tgacccgaaa aaattcgcgg ccttgtatct tcatgacaaa

9781 ggggttattc tctgtaatga ccccagtgcg agtaggttac attaatcacc ctgtccgacg

9841 ggtggcgtta agtttgatgg acatttacaa tgttttctcc gaacttttat cgagtctttg

9901 aagtcgattt acgtacgctg ttcagtcctt tgcacccatc tcggtctccg tctctcctat

9961 ccctcgaatc tgacgacgac gtcccagccg ctatgcagta cgttaactgg aacacctgcc

10021 aacaccgccc ggcactcact gccgccgctc gccgacacaa gtggtgctcg cttgtggacc

10081 tgtgtaattt tgtggaagcc cacaagatga acgggtttca cacccacgca tcccgcaacg

10141 ttgtcgttgc cgcgatcgcc cgcctccgga cccacgcccc gttctctcgt acagagagat

10201 ttccggagga cgtggagttc gtcgacttgg acgccacctc ggtgcttcct tacatcgacc

10261 agctccacca agcgtgcgac ctcaacgatc gtgccgtcaa cgtcggcaac gcgcagccga

10321 acggttccac taacgacgcg aagcgcgctt ttgacgtcgc tttcacgaaa attcgtgact

10381 tcgccaccgc cgttgacttc ccagaaggtt ccggtgtgtg gactcgcgag accttcgagg

10441 ctcgctatgg cttggagtgg ggcaacccgc cagccgccaa caactagtca tgtgtaacgc

10501 tacactggct ggtccctcgc ctttccctac ctccctttcc ttatcctttc ctcccttcca

10561 ggtttaaagt ggtctagtgg accacgccat cactacacgg tggtacacag aggtgcgatc

10621 cctccgtttc cgtggaacgt catccacacc

**Hubei virga-like virus 1**

**NC_033165**

1 gaaagggtat tgttaccttg gactgttcga aacagtactg atgggccatc cgaaagctcg

61 tctgtatttg tcaaaactgc gtaagcgtct gggttcgaac ccatacgtgc gcgtagttct

121 cgcggaactc ctcatgttgg ctcgtggtga tgtcttactg cctggtgtcg tacctgtcac

181 ctacggaaga ctacacgttg accgcatggc ggaaccgcaa cttctttcgg accttcttgc

241 tgcctacatg ttctctagag ccacgatcgg cggtgatgtc accgaagaga tggtagagaa

301 gatggacgca atacagaaat tcactgatct ctgtaaaacc gctggaatcg agacagcacc

361 ggccatggct aacgctgcgc agcgtgtcgt gagtcgtatc gcaacaacta acgctgacct

421 cgtaaccggt atgttgcgta caggaagcaa gaaactgggt gaccgtttcc cgatcagcta

481 tcagcttgag acctctgctg ccgcgcggct tgcaagtgag tatcccgagt tgaatgttgt

541 atttggtaac acccctcacc cccatgcata cgcagcagcg gctactcttt gtcaagagga

601 gctaattctg cgacggtgta ggtacgaccg taaaaatacc gtgcctaagg actttgacgc

661 tgcgatagtc gatattgggg cgaattacca tcgtcatgct aaagcaggaa gaacgacaat

721 acattgttgt tcccccatta tcgagcatcg cgactccgcc cgcgatacga ctagggcgaa

781 tcaactgcag catctcgtgt caaaaaatat aataacgcaa aggacgtgta atgcgtacac

841 ggacggaaag ggtagcttgc agaggtgtta caatctcgca caagactgct acgtcaccgc

901 gccttatgga atgctgattc acggacaata cgacatcagc atcgctgacc tgggaaaaat

961 gtgcgacgcg catcagttcg aacaagtttt cagcgtgatc atttttgacg taggtatgtt

1021 gttcaaagaa tccggcgtgg tcgctggggt cgaaatgaac tactaccgcg acggaagtga

1081 tatcgtgtgt ggatacgtag gcgactcgtc ggtttcatac cggcataagc tgagtaacat

1141 acaggaaatt attcgaacca ccatcatggt cacccccaaa ggccgcgtct attattgcga

1201 aaagacgacc cgaggtggtg cagtgttttt gcactacgtc tattgccgtc ggaaacctct

1261 caccgccacg cagtcagtcg atttttccgt ttggtatccg caagatgaga gcgccgtgct

1321 gctatcgacc tttgagttcg atcagactgt ctttggtgac agtaaggttc gcactgtgaa

1381 ggacccgatt ctgcggttta aacggcgttt catcgaggtg gacaaacggt tcttcgaact

1441 gttgttgggt cattgcattc gttccggtga taagtccttt aacctgaacg aatgtttttc

1501 cgcagccaac acgtttaata cccgtatgtg catcaatgga aatgatgtaa ggacagtgaa

1561 tcgtgttgcc agcgatccct gtacgcgatg cagtatatgc acactacagc cgtcaggaga

1621 tcctcgctgg attgcgggaa aggggataga agctttcgca aagtccgaga aggaaaaacg

1681 agctaagggt cgcgtgacct tttggaattt cgttgagtac gcttggaaga agagctctgg

1741 accccgtctc ctcaactcca tcggggagat atggaatgat ctgataaccg acatggctga

1801 attggctgaa gtcgatcaac ttagcgcagc cttcttccga tctgtccggg aggtacgtta

1861 ctccgagttc ctcactgtct caggtgagta cgaggaccat gacttaccat ccattttgat

1921 ggacgtaaag gtggatagtt tcgccatgaa aatggagaac gaccacttga aggaggtttg

1981 cgacatagtg cgaagcggcc tggtcggtga gaaaccggaa ttgtctaaac cgaacgtgtc

2041 cccgaagctc gagggtgaac cggttaaacc ggccactgac tcgaaagtaa aaaatttgtc

2101 gcaaccagga actccagtag ctgctcctaa gcttgtttca gcgcacaatg cagcaagtga

2161 gagttctcaa accgcgctga ccgccccgtc gactcagcag acctacttga cgcaagagtc

2221 tgttgtgcgc cccgtcgctc ctggcgagtc gcgccccgcc tcgtcggcag gttcgctgac

2281 tatgtcgatt agtgtggaca gtgcacctac tatgtccgag gagccgcgtc ctcttctgag

2341 ttccggatcc ctagcgatgt ctgtcagcga aatcagctgc gtgaacagtc tgcctgtctc

2401 ggatatgtgg gaccgtctgt cttcggtcgg ttctgtatcc agtatccctt ccctgtgcag

2461 tgcccgaagt aaccgcactg tgacacgcga aactgaacga gttcggtcgg ctttagtcga

2521 ttcggttcgt caggatatca tatcgatcgc ggcgtcggaa gttggatcag ttggatcagg

2581 taaacaactg cgtatgctcg cgacgaaatt gcatatgacc ggaactgaca agtcggagga

2641 gaacagccgc aggctgagca aggctaggaa ggacgccctc tcatacacgt gctccggtcg

2701 tcttacgcac gtaccaatgc ccgcggatgg acattgtctg tttcacgcat tggcccatgt

2761 gatcggctct acggcgttgg aagtgcgcaa acaaatcaag gctgctacgg agcctggaca

2821 cgacacctcc catctggaca ttgtcgatgg gtcacgagcc ggttggggtc gaattgaaga

2881 cactattgtc ttcagtagga tgactggtat tcgaatttgc acccatattg tctccccaca

2941 cgtcagtttt gctacagatg cgtattcggc cgcggaattg cctgaaaccg cacctgtcgc

3001 gcatttacat tggcaagtgg atggagaccc tatggatgcg agttataccg cgcatgttga

3061 cctgcttgtc gacaacaatc gatcgtctga ctctacgctc gccgtcgtcc ccgaaatacc

3121 actggactct cctgttaaca tcaccgaacc aacagcggtt gtcacacccg tcaccgtgga

3181 ggttgccgag gtggtcagag aggagacagt gtcgaacgaa cccctcgttc cggaagtgcc

3241 tgacaagact ggaattgccg tcacgatgct ccctgacgaa ctaaaggagc tcgttggtgc

3301 cggtgtcttg ccatggaagg aacttttcgc ccgtgtgaag aaacgcaaga atcgggatgt

3361 cttctcgaac ttgcaccgtg tcggtgtgtt gccggaccgt ctcgcttatg agttggatga

3421 tctgttcgcc accctacgga cacacacgga agagcgtttc gacgtgcgac ctatgaccga

3481 aaagtacaag cgattgtgta agtattggga cgcatccgga ggggattttg tggtcgactt

3541 gaaagccaag gagtacgttt ggacggcgcc cattgtttat cgggttcgag cgcatgtcgc

3601 ggaggatatg ctgctgaaac gcgatctttt cgtgttccgc agcaagttca ctgcgtataa

3661 tgacgattac gtatacgtgg tgaatggtct gcaatcaacc ggtgatcccg acgcgtggcg

3721 tgtggctacc gtccgctctg tagcgtccgc ctacactccc aaacacgttc cctacacgga

3781 gacgttgatt aaagagttaa tggtgcgggg ctattgtgag gacgtgttgg cccgaattaa

3841 cgccctcgtc aactgtaaga accacgttgt gatccggtct aattgcgtcg tttgcattta

3901 tgtgaacgaa aaacccaaat tgacgacgaa gatggtggag gcgatcctta aggtgatcga

3961 acccatcgtc cctaaaaacc ctatgcgttt cttcccggac cataagttag cggagaaggc

4021 tgtgcaactc gagtcgaaga gaactgccgg ttactccact gacggcaaca tggcaccccg

4081 tgacgagcga caaaccggct tagtcactgt tgttcgcagt gacgcttata aggcaggaaa

4141 ggctatggct gcacccggaa aacaccaact gcgaacggac gctgctaccg tgacttctac

4201 cgtgtacgga gttgaaggaa cgtcgaaaat gtccttcctc ggtgataacc gccttcgtac

4261 catcgcggaa aaaccccgtg aggtagccgt tcgtgagatt agtgccgtta atgtcgtggt

4321 gcaaccgaaa cgagtaatgc ggtcagtggg gtgcggaacg gatagtcaaa ctatcactgt

4381 ttcggacgaa ttccgtaaac ctgcctgctt cattcgttgc gccatggccg atcagggcac

4441 tcaagctccg gacgctaagc cgttggcccg cacggtagat gctgtcgtac cgactgccgc

4501 cggtgctctt atgaaggacg caaagacgga aacccaagtg atcccggcaa agtcgaaact

4561 ccagcgagtc gcctcactct tttctgtctc caagaagtct gttgacgaga accctgtcga

4621 catgatggag atagttaagt cattccccgt ccagaaaagt gctgaccagt tggtgaggaa

4681 cgcatggtgg gtttctttcc cacatgccct ccgaacgcac ttgtacctgt gtgttgcatg

4741 tcaggacggt cggctaatga taaccaagga gttgagtaag cgcttgggtg tcgggattac

4801 ggttccaatc aagaccttga ggacgaaggg ttacaccccg tttaccggag ccatctctgg

4861 gtggtggcgt acgtggttga cgggaaatgt cgaacattac gacgtcctta ttgtcgacaa

4921 tgcccttccc ctcgacactt tgcaaacttt cctttcgtcc cttccggaag atgtttgcag

4981 ggttactctg gactttgctg gtgaaccgga ccccatccgt ctgatcgacg tattgcgaat

5041 cgctaatcgc aaagtactgc tgactgacgt gtgttctggt gcccgcagct atgctgctgt

5101 cgtggccgta ttgcatcccc cgaagcgcga tccggttgtg gagaagccta tggttaccat

5161 gcccaagttg aacgcggaag atttaggaaa ggttcgtgat ccgcagttac tgccaggcga

5221 tagtcgtgaa atcgcatatc ggaatgcaat gttggagttt aagcatttaa ctgctaatgc

5281 cgacaacttc aacgctgcaa agtataaggc aatgctgcag gagtcaaaaa acgggatgag

5341 ctccgacgta ttagacaatc tacgtcggag tcaaatgaac gtttacgaca accacctaaa

5401 acgctttctg ctcacggggc agtcgaggac gtactctcac ggttatgcca aggaggggta

5461 cgttgctttc gacgagaaga accagaggtt cgaaaccacg gaacgatacg tgattacggg

5521 ccgcagtaca gaactcatgc tcaaccagcg tatagcacag cgcatggaga ccgtacctat

5581 agatcgggtc ttcatgccag taatagagtg gacaaatgga ccgccgggtt gcggaaaaac

5641 gtaccacata atccagaacg cccacatacc gatatcgaca acgcaggaac cggaggacct

5701 agtactttgc atgacgacgg agggtcggcg agatgtcatg gcaaagatga agtccaagcg

5761 tccggaaatg tcggacgaag tgctacagcg ggatgtgaga acagttgctt cagtactagt

5821 gaacgggtgt gcagtggcct atcaacgagt gttgctcgac gaagctttga tggcccatgc

5881 cgggacaata gggtttgtag tatattattc ccaggcgaaa gcaatagcca tgattggaga

5941 tatacatcaa atcccttatg tcgaccggga gcacatgtgt tcggtgctgt atcacgtacc

6001 atcccggttc gccgacatta cgaagagctt aaatcggacg taccggtgtc cagtcgatgt

6061 gacgtacgcg ttaagcgctt tctacgaggg tttgcacacg acaaacggaa ttatcttgtc

6121 ggtcaggcag ttaacttact cggggaacgt cacccatatc gacaaggcgt gtacgaacac

6181 attattcctc gtacacctcc aggctgataa ggacgcgctc gtgtcggaag gatacggtaa

6241 agcatccggg tccgctgtgc tcactgtcca tgaagcacag ggcctgactt tcaatcatgt

6301 aatatgcatc agacgaaact ccaaaccctt ggaaatcttc tcgagtctcc cgtatgcgat

6361 tgtcgcaatt agtcgccaca gggagtcttt tgtgtattac actgacgctg aagatgcaat

6421 caccggcctt gtgaagaaag cgaagacact tgatgcgtct tctcttagtg tctggaaccg

6481 caagcgttat gcagcaaatc ttagtgccgg tggtgtaatg cgtgccccaa tgacgattag

6541 ggaggatact catcgtactg atgaaaacct tacacgagac ttacccgccg acacgcataa

6601 cgttttggtt ctggagaagg acactggaat ttccttcgcg cttaaggaga aacctagtct

6661 gaaaggcgta tgggaggaag acctgtcgta cttgcaattg tggtacgatg acaaattacc

6721 gggagctgct ataatggaga atatgggagt gcaggagtgg atggaaacgg aagatacttt

6781 cgttagtctg gacagcattt cagtgaaccc gtcgctcgga ataatgcgta aaagtgggtt

6841 cggaaagttg cgtcctcgat tgcgaacgtt tatgttcccc actaaacgtc catcattcaa

6901 ggaatcgctt ttgggagctg tgaaacgaaa ccttaacgcc ccaaaactag ctaacgatgc

6961 tcttgaccct gattgtattg gacgcgcact attcaccaac ttcatccgtt cggcaatacc

7021 agctgaaaat ctggcgttgt tggacatgtt gtcggctgat actgtatctc tctcgtccca

7081 cctcgtcgac aagtggttgg acaagcaagc accggctgtc agacggcaga ttttgtctga

7141 aacccctttg cacctgcggt attataactc cttttcatat atgatcaagg gggatgtaaa

7201 gccgcaactg gaattgtctg ccatacagaa gtatccgagc gtacagacta tcgtgtatag

7261 tgataagaac ataaacgcta ttttttgtcc ggtgttcggt gtcctttttg agaggctcct

7321 gaatttactg gatgagcgga tactcgtatt taccggaatg tcaccgcacg agttcgagaa

7381 ggagctaaat gcgcgcattg cgggtttaga tttgtctgag gtcgtcacgg tagaggagga

7441 tatgtcgaag tacgacaagg cgcaaggtcg tgctcttagg caattcgaag atctcctatg

7501 ggacgctcta ggcatggatc cggacttgtt gcgaatttgg actgactcac acgtaaggtc

7561 gcatgtgcgt gatagacgga atggcattgg tttcgacacc gaataccagc gcaaatccgg

7621 tgacgccacg acgttcgccg gaaacacctt agtaatcttg gctgtgctcc ttggtgtata

7681 cgacatcgac gacattgcct tgatcatggt agcgggggat gactcgtaca tcttctttaa

7741 accagggtgt gcgcacttac cggatccgtc gcgacgcatt gctgaccttt ttaatttgga

7801 gtgtaaattg ctgcggaact tttccgtacc atatttttgc tccaaatttt taattcagac

7861 cccgcagtgg acgtatttgg tcccggaccc tttgaagttc gtgactaaat tgggcagact

7921 ggatatggcg aactacaagc acgttgaaga gtatcgcgtg tcttgtaatg ataccatgag

7981 tgcgctgttt aacccagttg tagcagaggg cctctctatt gccgttcagg agaggtatgg

8041 tggagacata gcagacataa ccaagctgat taacatcttg cggtcgttat gttcctgccc

8101 cgctaagttc gcctcactgt ttgttcacga ctatggagtg cgtcttagct ttgacccttc

8161 taccacaaag ttaaattagg gcttgtcaag gccaaagtta cacatgacaa ttcataaatg

8221 ttttgtgtaa atgcctaaga agaagcgaaa atctagaagt gcaccttctg acgagccagc

8281 cactagtacc gaagtgttac gcgagttacg tagtcgatct gtaactgcat cactagagtc

8341 tgaagccgaa acctgggaat cagacgacac cttagtgtca ccaccacccg aactagtgcc

8401 aatcactcgg tcacatcccg tccacgccca gcaaaccgtc cgcccgccta tctcgacccc

8461 acgcccggaa atgacgactc aaccacctgc ccccggaaat gcagaaggcc attacttggg

8521 atggaatgac gaaatggagc tgttgagtgc gcacagctat tgttccctgg tcgacttgcg

8581 gaactttgtg cacacctaca tgccgctcgc ctactccgtg catagtaatc gcgcgcgcgt

8641 actagaagcg cttaaggcct tgcgtgcacc ggccccgtat gccgagaaca tacgctaccc

8701 acagcgtggg ttttatgtgg acttgaacag cacgtccatc tcgcccatca tctcgaccct

8761 gatgtactgc tgcgattgca ccgaccgcgc aacgaacgtg cacggtcaga ctgcgagtca

8821 gcagactcag aatgacgtac gccgcagcta cgaagtcaat ttcgcgcagc tgcagtcgct

8881 tgcgtactgt gccaaggtcg atgagttggc gcgtaacggc attatggcgc gagcaatgtt

8941 cgagtcgtcg aacaacctga catggcagta atttcgccat tctttctact tttctgtccc

9001 ctttcctttt ctatgtgtta catgacctgt gtccttaggg accataactt ctcttcacgc

9061 acccctgttg cgtttttgct ataatgtcgg atggtatccg acaccgttat tacacagcgg

9121 cagcgaaggg ttcgattccc c

**Xinzhou nematode virus 1**

**NC_033728**

1 gggggggata ataacagtac cagaaattgc aatcgttgac gaattggtta tgattgatcc

61 taaggtgatc gatatcagga ccgaagaatt catcaaacag cgcttgcagt cgcacgagcc

121 taatccgata cgagatcttt atgatgtcgc gtgtgcggat aaaatacgtg cggagattca

181 gaaattggaa cgtcagaaag tagtgaaatt gactatatct gaggatctta cgaccgatca

241 agaaaatcga ttggtcaagc tttacccgtc gttgaatttg gtgtttacta agagaagcac

301 taattctcat tcatttgcgg cggctagtcg tcaatgtgag cttagcattt gcttacaacg

361 aacctcgtac gataaaaata tcgctcttaa gaacaccgac gggagagacg attatatagt

421 ggacattgga ggaaattatg ccaaacatct aaccgaagga cacgaaggta tacacagttg

481 ctgtcctatg cttgacgaca gagatattca gagatttgtg caacgtcgtg agaatctgaa

541 gaagcaatac gatctggaga aaaacaagat aaacgtgcag gttgtttatg acgaagaaaa

601 aactaaaaag gagagactta ggaagttttt ggaagaaacg cagaaaaact tgaagagcaa

661 tcagtgggtt tgtttgaaca agagtcaaaa ttgcgctaga acagctagat acggtattat

721 gatacattcc aattatgata ttactttgcg aaacatcgga gatattatgg tcacaaaaaa

781 tatgtcggaa atctatggca cttttatata tgacgatcga gtcttttaca tgactgaagg

841 atatatagcc gatttggact gttatttcag ctattctaaa gacaaagaat acattgaatt

901 tgcttttaaa aatgatatgt ctatgatcta tcgtcacaaa accagggttt atatgtctta

961 tttgttggtt aacacctttg tcgattctag tgggcgttct agattcgttc ttgaattgct

1021 ggaaaatagg ggcgggatac aatactttaa gattactaga ttaccttttg tagtcacaaa

1081 aaccttttcc cctcttaatc acaaaatatg gtttaaatct cttgaaaata aagttaaagt

1141 gacttttcct gtcgtggatg ttagtgctat gcgcaaaggt agtcttaaga aaatgattga

1201 gcaaaaaatc ttgtatcttg ataaggatct tatagacaag attatgagcc acgctatgac

1261 ttctactgaa aataaattta aacctgttga gattatgtct tttgtacaag catatactag

1321 tagaatattt tttggagaag atgtcttgtc tagaatgcct tctatgactt ttgacgaaaa

1381 ataccatttg tctttagctg tttatgtcga aatatttaga agaaaatatg atgcgggaaa

1441 ggttcttcag tttgtcttgg agtgtattta ttacgatcgc gatttgatcc aaaaaggttt

1501 cttcagaagg cttttgtcct ctcctactac tttgtattcc gttttcaaag ccgctttacc

1561 caatagtgtg ttttcttctt atttgggatc cttttatctc atggcattca acgcatctaa

1621 gtcaccagta gacggttata aattcatcga ggaaatacca tcatatcagg ctaatgatga

1681 cgaattaatg actcattacg ttactagcaa cttgaggaat agggttactg aagccatcga

1741 agacattaca tcgcgtctag ttaaaaatcc ttgtttctct caacttgaaa tatctatggt

1801 tcagtatccc aagattctcg taccgtcttt ggttctagaa cccactaggc tgactaggaa

1861 agttttgatg gataatttca gggttgatgt ttccatcgat catcaacaaa agattatacc

1921 tatccaaccc gtggacgatt ctaagattgg tatcgtgagt ctcctcaaga acaaaattct

1981 tcgcttgtgg aataaagaga ctaatttggt atcacctcaa aatgacgacg tcggtgaatt

2041 accagaaagt ttttctgcag aagatgtgtc gtctctagaa tctatgtctc tcatggatta

2101 tgacttctcc gatattagga ccaaccgtat aatacatcaa gatattatta atttagccaa

2161 acttatatct gaggaagttt ctgacgacgt atataagaat tacatggaca gaaacgtcaa

2221 gtgcgattat gactctaatg ctaaagattt catatctaga gcggaatata aattgaaaga

2281 aatagcagac atctatgctt tagctccttg tgacaatgtt ctggatcttt gtgctggtcc

2341 tgggggtttt accaaatttt tgttgactaa gacacttaaa aatttagtgg ttcattatta

2401 taaagactct gaagcacctt gttctttagc tatctctaaa ctctctaaaa tggattcttt

2461 tggcaaactg aaaattatgg atttgcttga taccgatttg ttagaaccgg acacccgttc

2521 tcagatagat actatgcttg aaaattttag gaattctttt ggtattatca ctgctgacgg

2581 ggctcttcat aacgatacat ttgaaaaaga actggaaaac tatcctttga taaaatctga

2641 gacggatatt atacaagatt atttgactac tggaggtacg gctgttataa agacttttgg

2701 attttatgat cacaaaacct tgtttatgtt gagtgaattc ctttctaaat tttctaaatt

2761 tcatattcat aggtcatctt acgttgctcc cttttctctg gaaatttatt tggtggctgt

2821 tggatatcgt tctagatctg cttctattaa gtcttcgact atatataaga atcttaaaga

2881 ctatatgttg gcttttgaaa attctcttcg catcaaaatg atcgcttttc ttaaacagca

2941 aaacctcact aataatgtcg attgtgtatt aacccctccc actgaattta gtgttgtttc

3001 aaaaactgac cttgagtatt tttccatagc ctctgaggag tctgatcccg aagatgattt

3061 gcttactgtc aatactgctc atacaactgc atcacctact acactgtatt cttcgaacga

3121 tgaagatgat tattgtacgg ctctttctct ttctacttgt aaacaagaat ccatacatgg

3181 tgaactcgta aaatctgata ttttggctca attatccttg gatcctactt gtcatcttct

3241 tatacaagag aacggtctca ctagtttgac tttttattcc tctaaatccc ctttttcaaa

3301 tttttatgaa attgaacttg attttcttgt ttctggtcga ttgcttaaat ttcggtctgc

3361 cgaacatgct tatcagtatc ttaaagctat acatcttggt caactacttt tggctgaaaa

3421 aattaaaaat tctaagactt cattgatggc aaagaagttg ggtaaatctc tcaattacct

3481 taaagagtct actacttgga tgtgcgttag agaaagtgtt atgtatgatg ttatatctac

3541 gaaatttcga caacaccctt cgttgaattt gttgcttgag aaaacctatc ccatgcccct

3601 catgcacact gtatcagatt cttattgggg tatcggtctt tctcattatt cagcttcgaa

3661 aaaatcttct ttagtttcct ctggtagtaa catcatgggt tgtttactaa tgaagtacag

3721 ggatacactt gttactcatt ctatatcctc tttgacgcct ccctctgatt ctactgattt

3781 atttttgaat tcattgggga aaaacaatta tatggacgtt agaggtgatg gtagttgtct

3841 ttattacgct ttgatgatgg gcgatactaa tgatcatctt ctcttaagat ctgctcttac

3901 ggcttattac aataatgctg gtccctttga cgatatagat tctaaaatgc tctttgccga

3961 attggagggt atgggcgggg cttttgtgct gaaactcttt tctcgctgtt atgattccaa

4021 cgtggtggta gcggatcttt gtaggaaaca agattattct tttggtgaag cttctaaacc

4081 attacactcc atatctttgg cttataatgg tactcattac gtacttaaga acacttgtac

4141 acttgatgct cctgttgtcg ttgttcccag gcattttaat tataatcctc ttaatgtttc

4201 tcacgttctg cttaccttgg ataatatgtt gagcaaaaat tcgactaagg tctctagatt

4261 cgtttcttat ttgttgggtt ctaaatcttt tcattatgcg acttttgaaa gtctggatta

4321 tacttctcac atgttttata atgcggtggc taactattgc gcttttcact ctcaatgcgc

4381 ttcgggaaaa ctttttactt tactgcaaga tcttaacgct catgattatg aaaagacttt

4441 atatgctttg gtttctattc ctgacactaa tttcgaacag ttgcaagctt atgttgatag

4501 caattctatg agatatttct taattaacat cccttggttg cacagagctc aatattctat

4561 tattattttt gcttttgata gaaaagccga taacgatgta tctatgtctg ctgccttgaa

4621 tttatctagt caacatattg agtcctgtgc tcactgtcaa aatgatgaat tttcatctga

4681 agtttctgga gtatggttta attctttgtc taacacttat tatacttgtt gtaaagctgt

4741 agatagcatt aacattttga agacttctca cttacccgtt gtagatagac tttataatat

4801 taagatttat gaaaagaaac ttttgtctcc tgatagtttt attatagata gagatggtta

4861 ttatgaaata ccagtttatt gtgatgttgg tagtgattgt cctgcggaag gctatatgga

4921 aattttactg cgaaatttag aaaatcattg tcgaaacgtt gaaagagtgc ccttaatagg

4981 tttggctaag acgcgactgt ccaacatcaa gaaaattgtt gatcttcttg ctacgacttg

5041 cgagcgtctt tacgtcgaaa tagaagaatc aacggaaatt cctatggaca tgacggtttt

5101 tagtccggaa tactgtcaaa ctctggtcaa caatgatata atgcttaatt ctatgaaaga

5161 ggctagagaa atgtggaaaa tcacagaaaa taccataata tccaatgtta agaatcttca

5221 caatcggtac attatgtttt tgaagggtac tgcgagggtt cctgatacca ctaatgacag

5281 accagattat ggattgttgg acttgaacac ggggaaattc atgatacagc ctagagaaaa

5341 acaccaacaa tattccagag gttacgatgg tactggcctt gtttcgttgg ataagtatta

5401 tgtggacggt aagtttaaac cgggactgtc tgttggtatg gtgtcggtta cgcgagatat

5461 gcgtatagtt aacgctgacg ctatatatac taatgtcaag aatgttgatc tgaaggatgt

5521 taatttagat cacgtgacta tcaatcttgt agaaggtgtg cctggatgcg gaaaaactac

5581 ttatatagtt aacaatcata agtttgcaat tgatgatttg agcgatgtcg tcttgacggc

5641 tactcgtgaa acagcggaag atataagaaa gagggtgtgt gttgcttatt ccgtatcgga

5701 agatttgcct attttaaaaa agagatatag gaccatagat tcctttttag ttaatttcag

5761 ttctaaagat gttggtatta atactttgtg gattgatgag ggcttgatga aacactttgg

5821 cgaaattatg tggtgtgttt atttgtctgg tgcgaaaaat gtgcgtatct gtggtgatcg

5881 ggctcaaatt ccttttatca atcgtaatgg tagtattagt ttattgtatt ctaaaatgga

5941 tgctttaacc aagcgctttt cggttgaatt tctgcagaat tcttatagat gtcctgctga

6001 cgtggtatgt tatttgaatt ctcttggaac gtatcccggc aaagtttcta ctattaacaa

6061 aactatgaga tctattcatg ttcagattgt cactggtata gcggatgtcc cctttttaga

6121 tttcaagcgg gctgtaattt tgacttacac tcaacgcgaa aaacaagaag ttactttgca

6181 cttgaataag atatttcctg gtaaagacgt gtcttatact gttaacacca tacacgaata

6241 tcaaggcaaa caggccgctg atgttgtgct tatccgtttg cagatgaaag agatcactat

6301 ctataatagt gttagtcatc aacttgtggc gttaactagg catactcatt cttttaccta

6361 ttacacggtc aaagatgatt ccttagcggt tatctgtaga agacagtatt ctaccaatca

6421 attgttcgac gctttacaag attctctagt gggcggtgga aagatgcagg ataagcagtt

6481 tcaacgcact caacatcctt tgcccttgta tacttatgaa gatagttcca ccagggacat

6541 tcttaaaact aacaagatta taagggaaat aattatcgat catgacggtt tcaacgtttc

6601 ttctatgcct gttagatctc gtggtgttga agttccgttt caagttattc ctgaaatacc

6661 tgtcagtaac atctcggatc ctgtcaatgc tttacagcat ttggttgacg tggtgtttcc

6721 tggagcttcg tctgtcgatt cccatttgga tagggttatt tttgaaggtg accctttgat

6781 ggttactagt gaaagagcta acatcattga cgagactatg cctagattac ctaagtatga

6841 caatctgaag tctcggttac gcactaattg tcctactcaa attatagcga ctcagaaaca

6901 agtggtcaag gcattctttc agcgtaatgg taatgtacct gatttgtatg gtgagaatga

6961 tgaaacatct ttggtgaaca aaatggtgga tgtcttcaca tccacttata tatcggatag

7021 agctcttttc gaaaaatttt cgcaagaacc cttagatata aatgtggctt ctattgaaga

7081 atggttgtct tctcaaccac cgcaagttaa agatattatt gaacaagatc cggatgttaa

7141 cgtattcttg aaagacttgc agttgtacaa cttttctttg aaacgcatgc ctaaaccaaa

7201 attggatatt ggtaatgaaa gtaaataccc tagttctcaa acaatagctc atcattgcaa

7261 aaagatcaat gctatatttt gtcctatcgt tcgagaactt aagaaaaggc ttttgtccgt

7321 tcttaagtat gacaaactta tttacactga tatgtctgtg caggaattcg aaaatatttt

7381 gaactataga cttccgccta aagattttgc taaatatact catatgttgg aagtagattt

7441 ctcgaaatac gacaaaagtc agggtagagt agctcttaag ttcgagcttg ctattttgcg

7501 tttattggga tttccaccgc aacttttggc tacgtggact tatatgcatg tttatactag

7561 actttgggct cctgatgtta ggtttaaggc ggatgtatgt tttcaacgaa aatctggcga

7621 tgcgatgact ttcttcggca atactatgtt tcttatgtct gtattggctc atacttttga

7681 tttatctaat gccttttgta tgttttctgg tgatgattct ttaatttttt ctactaaacg

7741 tttagattct ttggaaacta tatacaattt gtcgtttaaa tttaatctgg aaagtaaatt

7801 attgcattat aatacgccat atttttgctc gaaattcttg ttgcaaaatt atcaaggttc

7861 ttggtccttt attcctgatc caataaaaat gcttataaaa ttgggaagaa atgatttggt

7921 tagttatgat catgttaaag aatatcatat ttctcttaaa gataatgcta gacatttcac

7981 taatggtata tttttccctg ctttgagtta tgccgtttgc gatcgttaca aattgtataa

8041 agctgatttg acttattatt tttctgcact ttacactctt ttgtattctg agtctagctt

8101 tgaaaatctt tattatttag aacctggagc taatttaaat ccttatagaa tggtattacc

8161 ttctattgat atatagattc ctggttttta tattattcga aatttcttca taatccaata

8221 catcatgcaa tatattctgt tcttcctcgt ctcttcggtt tttgctgatc atggactgcg

8281 tgttcatcat gttttgagga ctaggaaagg atattttgat ccttatcttg aaaagtattt

8341 taagcccttg caacacgttc ttgatatgct ggctcatccc catctttctg attctgattt

8401 tgcggctttc actctgtcta aggaacaata caccaagaaa attcaagatt atatgtttga

8461 tatctataaa caatctaaat cttcacatca actggttatg ggcgtggcta aaagacgacc

8521 tgtggtttac actgctccag ctgacccctt ttatcctaga tttgcttatt acggtatgtc

8581 ggatacttgt tggtttaaag atcgttccgc taactttata tattttttga ataatcctga

8641 cccctcttgc atgtcggcta ttgatagaca cgaatttgag attactggtt gtaagttggc

8701 tagtcaatgt tcttatattc aagcccatca tttctttaat actactattt gttacgggcg

8761 cagaggagac aatgttaaat atatggcttt ggcaccttat aaaatccctt atgttcctct

8821 tactagaaat tctcaacaga ttttttctat ttactgtaac attactactt ctactatcgt

8881 tgttttaccc acatatttct tgcaatctta tgataatgtg gcgttggatt attataatta

8941 tactttgcat catccttata taactttagt ggatttttct tatcaaaatg tctttcttcc

9001 tatgcttcat tcatctttta gtcctttgca actaaaacta gatcaacctt ttaaatatgt

9061 ttgcaatgat tcgctttttg aaaaaacttc ttttgtagac gactacaacg tctttaggtc

9121 tcctagagga tgtgaaaact ttatatactt ttatgaatat gaacaacata ttaatatatg

9181 cgtcgattat ttatttctgc caacttcttc tttgatttct aaatgcccgg atgattggaa

9241 cgatttttct tctgctcgtg ctcctagaaa ctatgttact ttaattatta ggtctcattt

9301 gaatgacact tggtctatga ttaaatataa tattgattat tttatatctc gtctttcaaa

9361 aactattttg caaatttcta ctactacttt tgattctttt attcgtactt ttgatactta

9421 ttttctaaaa tttttagatc tttttcaaag atttctttcc gatttcgaaa gtggtatatt

9481 ttttaaaact agtgtttatg aaaatttttt cgcttatttg tctctgtttt taagtccttc

9541 tcaatttaat cttcgacaat tgattaagga ttctatagtt ttctttttta aaactatgac

9601 tggtaatttt actcaacttc cttctgacaa tgaaactctt tatatggaca ctaacatgtt

9661 cgggctcgat attggcaaat ggttagcgga cgcttttact tctattctta agcccttttg

9721 gcaacttttt ctgactatat tagaagacgc tttaaatatt atttctgatt ttttatttga

9781 tttggttcct cttctgcaaa aatttgtatt tgtttttcaa aggactatgg gtaaatttct

9841 ggacttgttg actactataa ttaaaatttt agctactttg cttctgcata taatagtcta

9901 ctttgataca aaaatcttcc tttccgaata tttgattttg tatatatttt tagcttatta

9961 ttggagatct actatacctc ctttgatctt tttgattatc ttaattttga tttttggtat

10021 tactagacgt tttccttctc ttttcttgct cttgttgaat aaagaattta gagatctgcg

10081 ttatctaggt tttaattcct ctttctttta tcacatagct tttaatacta catctcataa

10141 caatactcac gattcggtct ttatatttct ttctcatgga gatattaata tgactgtttt

10201 tatacccaaa catcatcttt ttgaaaattt cactttacaa aatatttttc tagattattc

10261 tgctataaat actacttcta tttatcaatc acttaattcc tccgaattta gcttttggtc

10321 ttatctcaaa aactttggta aattcatgaa tattactcac tcttaaaatt tattggttat

10381 attattgttt accttttcat attccaattt gacatgtcta ctcaacaact cctcgtgcct

10441 tcggaagata atagaccgcc taaattttac aacctcaaca ccggaattca atcgatccgt

10501 ggcggtcagc aggtgggtga tgggtttttc gcttcggcgg caaattcctt ctacaacatc

10561 atataccatg cttgggcttt gttatttgcc atactggccc ttcttattct cttgtcggaa

10621 tatggtacca gcgcgggccc ccttgagatc cttttcaaag ctctcatgaa atttaaggaa

10681 gaccctttcg ctcccgtcat tttgaaatcc atcgcgtcag gtatccttta catcttggga

10741 tacatgatca cctacaaaat ggcggtgggc tacgccctac ttctgctcgt cccagtgatg

10801 gtaaaaccat cggctcggaa ctttgtattc gcggcggtta ttatactgct tgcgtttatg

10861 cattacatca ctataataca agttctgatc cttgccatct tgttttattt atttgtgatg

10921 ctaagaacac cagcacacaa atttttcata gtggccatgg ccgttgctgt gtttggagtc

10981 ggtattacca ccggtccgga ttccattcga tttaacatca ccagctacaa tcttgagact

11041 tacaccaagc cactttcttt taaacctaat ttagaacttt tcaaaagcct tcccacaacc

11101 ccatattctt taactaaaga ccctcaggat cgcgatatag aagatataca taaagattat

11161 tatgaattgc gcaataatgt ttttaatctt ttagatcata ttgaacagtt gggcctcgat

11221 agaagagccg cggctgccag cactaccacc gctgctacta catccaccgc gcacgccccc

11281 tctgtagcca cttcgaccac gaccaaggct accgaaaaac ctaaacaacc ggttcgtact

11341 ggaccggcga ggggcgcaca ggctgagaac gtgaagcgat ggaaagaagc cggaggcgac

11401 gaactgttta aaccttaatt ctgttttatt ttatatttct tttatttttc aggattattt

11461 tcaaaatttt atactatatt ctaaatttta ttcctatatt ctttattact ttccctatcc

11521 ccaaa

**Xingshan nematode virus 2**

**NC_033727**

1 gaggataata gcagtaacag tacttcgagc tacgatggct ttggtcgatc ctttgttggt

61 tgattctaaa gctaaagagt tcatagagca acggctaggt tcacatgagc cgaatcccat

121 tagggatttt ctggatcatg cttgtgccga taagttgcgt gaagagtttc ggagaatgga

181 gaaaatgaga gttacgaagg ttgtaatacc tgaggatctg actagtgatc aagaaaataa

241 attgatgaga ctttatccgt ctctcaattt gattttcacg aagaaggctt gtaatgccca

301 tagttttgct gcagctagtc gtagatgtga aggagctatt ttgctacaga tgattggtta

361 tgaccatgat gttgtggatg aaactgacgg acgcgacgat tatgctactg atgttggagg

421 aaatttttat aggcatttgg cggacttgaa tagaggaata catagttgtt gtcctatgtt

481 agatgatcgt gatgtacagc gatacgtgca gagaagagaa gcgttgaaga aactttacta

541 ttcggatata aagaaaatta aattaaggat gtgcggtgat aggtgtggtg atgcacgaga

601 cgaagaaatt agaatatttc tcaacgacac tcgggcgcgt gagtttcgaa ataagtgggt

661 ttgtttgcag aaagctcaat tttgcgacag aactgcgcgg tatggaatta tgttgcatag

721 taattatgat gtcacactca gagaacttgg agatataatg actaaaaaga agatgactga

781 gttgtgtggt tgtttcatat atgatgatcg gattatgtac atgacagaag gctatatcga

841 cgatttggaa tgctactttt cttattctaa gaatcgcaaa tatatagaat ttactttcaa

901 gaatgattcg gcaattactt acaggcacaa ggttagagtc tacatgtctt atttgatggt

961 caatacgttc attgattcta ccggacttaa tagatttgtc atggagcttc ttgagaatcg

1021 tagcggtatt cagtacttta aagttactag tttgccctat tcagtctctg tggtcttttc

1081 tcctttggtc cataaagttt ggttcagatc tttggagggt aaaatgaaag ttactttccc

1141 tttaactgat cgggacgcat tgcggagtgg taagactgca gatatcgtta gagaaaaggt

1201 gatgtatctg gataaagatt tgatcgataa agttatgggg catgcaatga cagctactga

1261 aagcaagttt aaaccggtcg agatattgtc ttttattcag gcttatacgc atcgtatatt

1321 ttttggagaa gatgttacgt ctagaatgcc gtctatgacc tttaatgaaa agtatcattt

1381 atcgctggct atttacgtcg aggtctacaa gaggaaatat gatgttggta aaatggtaca

1441 atttgttttg gagtgtatac agtacgatag agacttactt tccaaaggta ttataaaacg

1501 attcttctct tcttcgcgag ggttgtattc cgttttcaaa gcggctctac ctgcgtcatg

1561 ttttcaaagt ttattaggac ctctctattt gatggttttt aacatggcta aatctcctat

1621 tgatgggtat acatttattg aagaagtgcc gcgttatatc tctaatgagt ccatcgggtt

1681 ttctacatat attgttacgg ctattaaaaa taagatagaa ggattgtttg atcaagcttt

1741 tgataggatg tcttgtatac ctagattctc cactttacaa atagtaccgt tggtttatcc

1801 gaaggttttg gttcctgaat tgatttgggc tccagagcga gttgttaaag ctgttatggc

1861 cgataatttc aatgtggacg tcgaaattca aacagctgtt gatggtgaaa aggacatatt

1921 gagcgacggt gcattgactg agcgtgtggc tgatgatacg aactggtttt ctagaatgaa

1981 aaataaaatc ggaaaaattt ttggtagtaa gactagtgcg gacagagacg aggtaaaaga

2041 aggtatggat ttcgatcttg atgggatgtc tgatattact gatgttgacg ccatagcttg

2101 tgattttgcg gacttgaata tgtcagcgaa agtaaaaaaa gatattatta tgtttgctaa

2161 ttatatttct gataatgtac cggataaaac ttatcgtgaa tatgtcgaga gatacactaa

2221 gatggattac aacaagaacg atcctatgtt tatatcgaga gctgagtata aattaacgga

2281 aatattgaat cagtactgtt tgcgtgatat caagaatgct ttggatttgt gtgccggtcc

2341 tggtggtttt tcgaaagctt tacttggcaa agttagtgac acgttggttg tgcactatta

2401 taaagaagct gatgctggat gttacatgag tattgataaa cttcgtcgtt tggatttgtt

2461 tggtaaattg aagtttttgg atttgttgga cacggacttg acttcggatt ctgttgttga

2521 ttccgttatt aaagatgcgc agaaacgttg ttttccttat gaattgatta ctgctgatgg

2581 tgccaagcat aacgacgtca ttgacaagga aaatgaaaat tattctctta ttgctggaga

2641 agttagaatt gcgcagagtt tattggccga cggtggtgtt tttgttttga aaacctttgg

2701 attttcttct catcgtactt tgcaattgct tagtgaaatg ttgatggttt ttgaaaaata

2761 ttatattcat agatcttcgt atgctacccc tttttcgacc gaacattata tcgtggcggt

2821 ggggtacaag aggaatttca gttgtgccag gaagaccagt gccgataaat ttagaagagt

2881 gatgatttac agagatttgc gaaatttcat gatgggcttt gagaatgcat tgcggaatag

2941 atactttaaa tttgttgacg aaacgatcgg tcctgatgta gtgcagtctt tgttggaagg

3001 tgatttgact attttgtcta atgattccaa ctctgctgtt ggtgatgctt gtgttggaga

3061 gcaagttaaa cgtgtggatg atagagttga tgaaatttat acgctttgct atgacaaggg

3121 catggatgtc gatagtagga gcgagtatgt tactggatcg gagaatgtcg gcagtaataa

3181 ttcggattgt gaagttgttg aaccgcttgt tgagtcttac gttagcgatg gcattaagat

3241 ggattttgac aaaggatact gcgtttttgc tggcagtgat tgttgtttgt ctaatataca

3301 tgttgaggac tttgaatttg tatacaagga tcgtatatgt cgagttaata ctatcgagca

3361 ggctgtggct ttcgcttgtt tttgtgtttt tgaagttcag catttttgtg gagcggacat

3421 gcgcactata gctagcgcgc ttttgcgaaa acgaatgaag aagttgttga gtacagaaaa

3481 gaataaagta gtgtgggatc gaattaagga cgaatgtatg gaacatgctg tacgtacgaa

3541 aattcttgca tctactatta tgaaagaagc attacgctct actaaggact gcaaattgtt

3601 taacgcagtt tcggacgatt attggggtgt gggagttact ttcaaaaatt tttcttatga

3661 acgcagttcc gaatatgtcg ggttgaatgt ttatgcacgt atacttgaga aagttcgttc

3721 ggatatgttg gacgaagaaa ttagtatgaa cgatgtcgat attgatgagg atactaagat

3781 cggagaaaat tttgttgaaa gttatagcgt taaagcggat ggtaactgtt tatactatgc

3841 gttgatgagc ggagatgata acgatgctgc gaaattgagg ttggttctta aagaggctta

3901 tcgacaaggc tgtggacgtg acaatgacga cataatagaa aaggaacttt gcgaagagct

3961 tactggatgg ggcggtcgaa gtgtgttgtc tctgttttcg caatactatc atgcttttat

4021 tgttgtcagg gatgtgaaga atgctgttga atataagttt ggtgaagata ttgttaaacc

4081 tactcttata ttacggttgg cttatgatgg tagtcattac agtattttac catcgtgtag

4141 tgacagagtg tgtactcgtt cgagagaaca tactgtatat gtgtcgcgga cttttaatta

4201 tagtccggtt tccattcctt cgcttatgaa gactcttgac tgtctgctca acaaaaattt

4261 tactatattt caaagctttg tggcgtttat cacgggttct cagacgtttg catatgtgtc

4321 gtcgctgtct tccatggatt attctcgtca tatgtttttc aattctgttg catatttttg

4381 tggttttcat tcttcttgta attccgcttc tattccggct ttactggatc agctgcgcac

4441 caataattat tccaagactt tgtatatttt tgcttccatt tctgatatgg actttgatgc

4501 tatccaaaga gttttggaac gtaccacctg cagatacttc ttaattgcta ttccgtggct

4561 tctcaaaaat ggatacattc tttttgctat tacttttgtt ttggataaag aaaatgaagt

4621 tcctttggtc gaagcctgta atttggctaa gatgcatgtt gatcagtgcg ccacatgtct

4681 tggtattaat gatgacactt ctattactgg ggtgtggctg aatagagcat ctaatactta

4741 ctttgtttgt gagagtcgag agtttactag aagagtcttt aagactgaaa ctttgccaac

4801 agtggacagg atttacaaaa tttttcaaca tgatcttgca gttcaatatg atactattga

4861 tgttgttgca agagccgata atgaattttc tattgtggtg ccagtcgatg ctggacggtt

4921 gagtgacagt tttgatcgtg aggcttatgc ttcctatata gtgcgacatt tttgcaaatt

4981 tggtggagtt agtaccattg gtattagcag atctgctttg aacaacgttc gtgagttgct

5041 tgatcctttt cttacttatg tggacattgt ttatgtggaa gttgtggatg acgttactga

5101 aaaagctgaa gatcacgttg agttttgccc tgaatttaag caaacggtta taagtgatga

5161 tgtattgatc aatgctatga ttgaagctag agatatttgg aaagttagta atcagatgat

5221 agtggacaac gtcaaaaggt tacatgatcg tacgctctca cttatgactg gcatggcagg

5281 caggattgag ttcactaata ataagccgga ttatggatta attgacttgt ctacgggcaa

5341 gtatttggtt agaccacgtg atggttgtgg taaatattcg cgtggttttg atggaactaa

5401 attaattgat attgagagat gttttgaagg tgatagattg ctggcgggtg ttatgtgtgg

5461 tagagtgtca gttacgaaag acatgagaat tgttaatgcg gacgttattt atagtaatgt

5521 caagaatata gaactcaaag acatttcttt tgatgatgtt tgtattactt tggtggaagg

5581 tgttcctgga tgtggtaaat ctacatacat attgcgaaat cataagtttt cggttgacga

5641 tgttaaacat gttgtgttga cggctactaa agaaactgca gaagacatgc gaagacgtgc

5701 ggcagagatg tatggtgtat ctagtgaatt gagtattttg cgcaaacggt atcgtacagt

5761 tgattcattt ctggttcatt gtggtggtat gttagaggag gattgtgttg ttgaaacact

5821 ctggatagac gaagggttga tgaagcattt tggagagata atgtggtgtg tgtatctatc

5881 taaggctcgg caagtgttta tatgcggcga tcgtgctcag attcctttca ttaatcgaaa

5941 tggcagtgta aaactttatt attctaagat agatttggtt ctggacagta ttaaagttaa

6001 atttttggac aagtcttatc gttgccctgc ggatgtggtg gctcatctta atgcacttaa

6061 tgtttatcct ggtaaggtta ctactgaaaa tacggttgtt tattcgatca aagttcgcaa

6121 tatcacaggt ttggtggacg taccgtttac tgattggaaa gatgcgactg ttttgacgtt

6181 cacgcagcaa gaaaagacag atgttatttt gcatgttgga aagtttttct cgaaaggttt

6241 atttgatgga aaagttttta cggtacatga gtatcaaggc aagcaaacta aaagaatttt

6301 gttgataagg ttacaggtta aacccatatc tatatatgat agtgtgagtc atcaacttgt

6361 ggctattaca agacatacga gggaatttat gtattgtact gttaaaaatg attcgttggc

6421 cactatgtgt cgtagaacat tttcagtggc gcaattgaag aaattgatag ttcccacatt

6481 gaaaggtggt ggagtgtcgt ccgatgacga taaagtgcaa ttcccattaa tgaaaacgtt

6541 agcggtggag tcgttagcgt ttgatgcgtt caggaagaat aagtatttga gtgattttgt

6601 aataaatcat gatggtgtta atgtcgttcc atatcatgtt gagacttccg ccgttgatac

6661 gtatgaattt tctgtaccgt atatcgcgcc tgtgtctatt agcaacccca tatccgcgtt

6721 acaagaattt gtcgacgtgg tgtttccagg cgcttcgact gctcaaaagt tttatgataa

6781 tgagattttt gagggagata acatggttgt gccgcgtgat agagttatta ttactgcgcc

6841 ttatgtacct cgtttgccta gatatcaatg tttgacgtct cgtttgcgta ctaattgtcc

6901 agataatgtc gttactacac aaaaacaagt tgttaaagct tattttcagc gtaatgggaa

6961 cgtgccggat ttgtatggtg ataataacga agaccttttg gtgtctcaga tggtcgatcg

7021 ttttgttcga acgtatatag ctgataagca gttattcaat agattctgcg tggaaccggt

7081 gggtgttaat gttgtgtcta tacaggattg gcttgccaca caacctgtta aagttatgga

7141 tatgcttttc aaggatcagg acgttaatat cttcgttaaa gagttgcgat tgtataactt

7201 tgctttgaag agattaccaa aacctaaatt ggaattaggt aatgaaagta agtacgttag

7261 tcctcaaact attgctcatc attgtaaaaa ggtgaatgca atattttgtc ctgttgttcg

7321 agaattgaaa agaagactat tgtcggtttt gcgcgctgat aagatcgttt atactgacat

7381 ggctgtggaa gattttgaaa aaatcttgag ttatagactg tcttatgaaa aatataaaca

7441 atatcgttat atgatggagg tcgacttttc caaatatgac aaaagtcaag gcagagtcgc

7501 tttgaaattt gaattggcta tacttaaaat gctgggtttt cctgaagaat tattggctac

7561 atggagtgtt atgcacgtgt atactcgatt gtggtcgccg gcggttaaat ttaaggctga

7621 aatatttttc caacgtaaat caggtgatgc tatgaccttt tttggtaata ctctgttttt

7681 gatggctacc ttggcacaca cctttcctct tgaacgagaa ttttgtatgt tttctggtga

7741 cgattctctc atattttcta ggcgaaagat cgagagtgtt gattcgatct tgaatttggc

7801 gttcaagttt aatttagaaa gtaagttatt gcattacaag gtaccttatt tttgttctaa

7861 gtttttgctg cgcatgcctg ttggaaactg gaagataata ccggatcctg ttaaggtttt

7921 ggttaagttg ggtagaaatg atttggttag ctgggcgcat cgagatgaat atttgatttc

7981 tttgcgtgat aaccttaaag attataaaaa cgcttatttt tatccatttt tgagtgctgc

8041 tgtgtcggat cgttattctt tgtgccctgc ggactttacc tattatttta gtgcgtttgt

8101 gtctttggtt tatgatgata agaatttttc aaatttgtat tttttagaag aaggacatta

8161 tttaaatccg tatcgtgttg ttttgccttc tttggagttt tgaaatgttg ttatgctacg

8221 gtaattggag gtatattacg actttgtgga attcataata attgcgctat gcgtgtgtgc

8281 gttttgttga tttccttttt cggtgcgttt ttgggttcca ccgaacgttt tggacttaga

8341 tatcacgaaa gagctcgtaa aattcctggg gcttatgacg tttatttgga ctcgcttatt

8401 tcgaaatatg aaaacgcttc ctactattta aaaatttttg ggcttgtgac tagtaatcag

8461 gctaagagat tgattgaaga gcttgactct cttagaaagg aaatacagga cagagtggat

8521 tatgtttccg aaaatcataa aagaatgtcc atgataccgt ttggtgtatt ggcgaatcgt

8581 tatattgttg atgatgccgt cgttgcaggt tttaggtcta tcgactatta tcgtagaata

8641 ctgtcctttt gcttggctga tttggaatct cgtattctta ctgtttctga gacgtttttg

8701 gaccctgtgt gcatgtctgc ttataataag tcgtttaatg aagatattga ttgtcgtctt

8761 tcctccttat gcttcggtgt tatcaagaag aacgttgatg gtctctttat atgcagtggg

8821 tactctaaaa atttgtttcg ccattatcgt cttgccaaat ccactttgct tgctgatgtg

8881 tttcctttgc gagagggaca gtttgaattt tattgcaatg tgtctaataa tcgtccattg

8941 cttgtccata agagtctgtt gaacggtgac aaaagatttg tagatccctt ttcggttctt

9001 ttgcagagtg attttccgtt tttgactttt tctgatggtg gatatcgcgc tcttgttttt

9061 tcgcagtttg tcgattctta ttctactgtt tctttggcta ctgccggatt taagtacgtt

9121 tgtaacgata cattattcgc ggaatcgtct attattgatg atgtcaacgt ctttagaagt

9181 tttcgtggtt gtagaaattt tgttttaacg gaaggttctg acgtgtcttc taagatctgc

9241 gtagatcatt tgtatcagga ttttcaagct cttgttgatc ggtgccccga agattggtat

9301 acatttgacc acgctgtggc aacttttgaa acatatacta ttggaattaa tgattattct

9361 gaagttttgt ggaagacgat accacaagtg gttaccgatt tgcttcggta ttttgataga

9421 aatcatatta atgttcgcgt tcaagatttt gaaaaatttt acaaggactt cgttgactat

9481 gtaagaagtt tcgggacttt gacgaatgtc actgtacggt ttgcggatca agtttttgat

9541 ggaattggcg cgaatttgcg acttttggat tcgtttactt ttcgaaattt ttccgaaaat

9601 tttgttgact acttttgggc aaaagctatg ggtgttgacg ttgaacaaat tcgtgatact

9661 acacattttg aatctattca tagcgttact tcggtatctg gttggttggc tcaagctatt

9721 tcatcttttg tgcgaccttt ttggcaagtg tttctcgaac ttttggaaga catattggac

9781 attataattc gtgtgctttt tgatttgact cctctgctcg aaaagtttta tagacttacg

9841 ctcgcggcct ttgatagtct tttgaatctc ctatttcaag ttctaacact tgtcttatcc

9901 tttttactgc atatcttaat tattttggaa tcaaaaattt tgctatctga atatttgttg

9961 gtttatcttt ttctgtctcg ttactggtca tctcctgttc ctccacttct tattttgctt

10021 ttgcttatat tggtttttgg ttttcaacgt tcttatcctt cttttctctt ttttgtttta

10081 aatcatcaaa ttaaatctgt ccttaatgcc accgcaattc cttcagttac ttacgattat

10141 caatttgttt ataatatcac tcttgttaat tatactcatc atctctatca tttttcaatt

10201 gtaaatacac cctattcata ttccttctat gtttctaacg tttctgtttt tacttatgtt

10261 tctgaaaaat tttcttcttt taattccaca tttttcgtta ctacattttc tcattatctt

10321 gattcttccg atgattgtga ttttgtttgt ttcctaaaat ttttcggttt ttaaatttat

10381 tttaagttat aaaatttata ttaattttat taactttcat taacaacaca cttggttctc

10441 atggcgtctt cagcgttgat gttaccgtcg gaaaatggca gaccgcctaa attttacgat

10501 catgctactg gtattcagac tgttagaggt gggcaacagg ttggagaagg tttcttttcg

10561 tcggtcttca attcgtttta caacattatt tatcatttct ggggacttac ttttgcagtt

10621 ctcggaatct ttactttgct ttcggaatat ggtacggcca gtggaccgct tgaagttttg

10681 cttcaagcta ttttgaaatt tatatcggat ccggacgttc ctattgttct taaatccatt

10741 gcttccgctt ttgcgtggtt gcttggatat atggtgaaat ataagtacgt tgtggcttat

10801 tcagctatat tgtttgtccc ggccattgtt aaaccctcga cacgcaacat catgttttca

10861 tttttgttga tattcatggc gtttttgcat tatatcacca tattgcaggt gttgcttctg

10921 tccttacttt tttatttgtt tgtcatgctt cgcacaccgg cacataaatt ttttgtgttg

10981 ttcatggctt tgatagtttt tagtgtcggt tttacggaac atcaaagttt tctttctagt

11041 aaacttaatt tgctcctccc ggccgacttg tctaagccct tgacttttaa acctaataca

11101 tttttgttac ccgacgcgca atctccatcg gcacgcgcaa aaagggatga aaatgaaatt

11161 ttacgtcatc aagtcgctca attgatggag aagatttcga atatgcatca agaaatggag

11221 accatgcgac aggctgcttc aaaacctaat gaagctacta ctgcggctcc tactgttttg

11281 ccacgtactg aacgccataa atcctcttga attaactagt ttttctttaa

# Lodeiro virus

**NC_031748**

1 catgtgcgct actggtgagt tccatcctta aaacgcaact gttataagaa tgaaacaaac

61 aaccaacagg ctataaaccc attggttgat cttatttcat tcttataaca gttgcgtttt

121 aaggatggaa ctcaccagta gcgcacatga gacgttgagg gcctatttgt cggtctgtgg

181 catctctgtt ggcaacttgg tgaaaagtta tgcgcaggat caacttaaaa atcctgattc

241 gcaagtaaag gtttttctcg acgaatatat cgagaaggaa gccagtaatg aaaagctcat

301 aagatctatg agagaaaaat gcatcattcg agaaagtctt cgaagtgagt tgaaaacgag

361 gttacttatg gagttcccgg aagttaggat agaattcgca gccccaaggc attttggaag

421 tcactcaata gccagagcct tacggcacat ctgtagggta aagatgttga cttccatggg

481 attccataaa ctcgaaaaga gagggttttt ggtcaaggat gtaggttcct atagtgcagg

541 agtagttctt tccggcgaaa ccaacattca catgtgttgc cccatcttaa gtcaccgaga

601 tgcgcaaagg cattctgata gtatgttgga tatcgagatg aatttagacc agctggaaga

661 aggaacgaga atcagggccg agactttcat cagacagtgg agcgagttga ataggaacgt

721 cgggaaaaga ttcgatcact catacatggt atgtaaaaac atctcgcagg aatgtagtgt

781 acaaagtaag ttttgcatgt tcaaccatag tagctatgac atcagtgtgg aagacatggt

841 tgaagtcatg gaaaaagctg gtagcgttag ggccaaagga gcgttcatct ttgatcagga

901 actcctcatg aaagatagag gagaaatacc tgatatagag tgcatgttcg aaaagtacga

961 tgaaaatggc gtcaaacgca ttaggttttg gttccgtgac gatgtgtcta tagcttacga

1021 tcatgactgg aagaagtaca ttagcatatt cagtaagttc agcgtgaaaa gtaaatcagg

1081 aaagtattat tatgtacaat tgaatgaaca taagttttca catattttct ttactatcac

1141 taagaatatg gttagagcca atgttcctaa agagataatc cagagaccag tttgtccccc

1201 tatgggagag gacttggttg tgcttcatta ctatgattgg gatgaaacag caggagtgat

1261 cgagtatgaa aagatggttc ctacgagggt catagttccc aagagattgt atgaatcggt

1321 ttcagcctat gtcaggaatt taccagccgg taaaaccgat atctcgcgag ctgtaacgtg

1381 tgcatcttca tacaacacca gaatcgtgca aaatggagta gaagttcaaa ttcccaagag

1441 tgtcccagct gacatagctc aaaaactagc tcacgcagtc ctttttgaag tgtatgtaag

1501 cagatacaaa gacagtaaag ttctccaaga acttcaaaag gatcaaaacg aaattcgaga

1561 cacaaggggc ctttgggaaa gtctgaagtt aaagatatcc agactgaaag accccaagcc

1621 agtaaaaatc tacaaggacc caatattgac ggacatcgac catcctgaag ccgtcaaaac

1681 agaacatagt tggtggttgc ccaaggtaat attttgtatg attcgtaggt tcagagagtt

1741 gacaaaacca gaaagcaaat atgactatcg agtatatgat ttcgtcaagg ttttcgattg

1801 gaatatagtc tgtgaggcag cgaaactaga ggcatctcca ttaccagact atcacgagta

1861 tgtcgaaatc ctggaaggac ccgacggaga aaaacatgat gaggaagaag taaaagaaag

1921 accgaaagaa aatgtaaaga aaacgagggc gcccaaacca gctcctctct ttatatcgga

1981 taagtgtcaa gaagaagata ccgattttga aatggttaat gcttcctcta cgtcgggaac

2041 tcatgaaggg gtttgctgtt atgacgcagt taggatagct ctgcttaaaa agaaaacttt

2101 agacgaattc ttgaaagagt taagagaaaa aggggtgaat gtcgactgtc aaggagacat

2161 tgacttctta atatgggttc accaacgaga gagagtaaat atatgcttac atagtgaatt

2221 agaggaccac cccgaaggaa taatgtacac cagaatagtt tctcagcagt ttaatcagtg

2281 gattcactta ttctttagac aaagtggtga tagtggacac tatcaagctt atgtgaaaaa

2341 gagtaaagct gaagaaacgt tggaaagtct ggatgagggt acatttgaaa atctaaagaa

2401 gaaagtctac ccagatctga aaagtagcgt gtgtcgaagc gaatacaaac tgtttgagat

2461 ccttgagaaa caccaaatca tggaaggaac aggaaagcga gctttggatc tgggagcata

2521 tccaggagga tggtccctct tgctcaaaca attgggttat catgtcacaa gtgtcatcca

2581 acctcaccaa aaaataaaag aaaaacaaac attcgtggtt aggtcaggga tagaggcatt

2641 cgaaggtaaa cttggtttcg atttaattgt tagtgacgca gccccagatg acctagatca

2701 actaatgcaa aataataaca aagcattggt agaattttac aagttggtct ggaacaagat

2761 cactcaggtc tcgaaaggtg aagaacactt cgtcataaaa atgcacttgg tggacggaat

2821 tcatgaagtg cttgaaagca tgaaagttga attcgttcat atagaaagac ccaaagtcgt

2881 tcgaaagata agtagagaat tctacttgta tggaagaatc ggacgaacgc cgatcgatag

2941 tcagaacctc ctattgaaaa taaaggaaat gagagacgaa ggaattattc gatacctcaa

3001 aggggaggct gagtacgaaa gtgaaaaact ggaaaatcag tatgaaagta cggtcaaaga

3061 aaaggagaaa gaagttgaaa gtagtaagta tgaacgaaaa ttcaaaatct gcagacaaga

3121 atacgttaac tacttggaac actcagtaga cgaaagagaa aaacaattgg ccaaaaccgc

3181 cgaaacttac aagaaacacc cattgttcag ggccagggct tacaaaagaa ttcaaatagc

3241 tatcaggcag tcgcccgata acatagcttt ggtaagaaaa gaagcagatg gacagctgaa

3301 agcattgaca gatccaaaaa gtctgaaacc agagtacaaa tatgcttata ggtatgaaac

3361 gaagaaaata gaaggagtag aaaagatggc caaaggagaa atgggaatgg taagtgattt

3421 caccgaattt ttaacggaca agaagatctt agaaagggtg aaacttatgt cgatcggtga

3481 aagaaaagtt caatacagat tcatacaagg agttccaggt tgcggtaaaa ccacatggat

3541 agtacagaac ttcaaaccag gaagttcgct tgttttggtc tccactgtta acggaagaga

3601 tgatgtaatc ggtaggttga aagaagaata cggaggagaa cacaatagga atgtacaaac

3661 ctatgcatca attctcatga atggaccgaa acaagaagga atcgagtggg taatatgtga

3721 cgaagcaggt atgcaacacc cgggagctat tgatttctcc attaaaatga cacaatgtca

3781 aaaagtgacc gttttgggag atggaaatca gattgccttc attgataggc atcactttaa

3841 tatagttcat ggagatttgt tagaaattct gaaggctgat gaacacctca gtgtaagttg

3901 gagagttccg caagacatag ccagttattt ctcggatcaa tatcccggag ggttcatgac

3961 cagaaacaaa gttagaaaat cagtcaactg ggtcaagatt cagagtttaa aatctttgga

4021 ttgggatcat gatgtgtatt taaccttcac ccaacaagaa aagaccgagg tattgattga

4081 aggaaaagga aagaaagaaa acatcaaggt gagaaccatc catgaatatc aaggggatca

4141 agcccggagc gttgctattg ttagaaacag agacaaagat gtaaatagaa tatatgaatc

4201 tgacgagcac atattggtgg cattaactcg acacactcaa aagttggttt actacagcgc

4261 ctccagtgac gataaaatga aaagaatcat caagaaaatg caaacattca ccgacgatcg

4321 attggacaag tcgttttatc actccactgg agctggagag gatttgttga aaatgagaat

4381 gtattttcaa caggaaaatt tgcctcctca gggacaatat accagacatg tcataaattt

4441 gatgaaaaca tacagtaatc tggtggttgt cgaatcaggc accgatcaaa aagtatgtag

4501 gatcggaaag aagatgagga tacaggaagc gattaacatc gttttggaga cgcggggaaa

4561 gaaatccatt ccatttgagg tgttgaaaaa attaccagag aaactgataa tctctgtaga

4621 caagaaaaat caaagcgacg aggtcgaaaa aatggctcgg aggcttagaa aaatgaaaaa

4681 ggaagtgctc ttgtacgatc gagatagttc ccacaagtat gtgattgatg ccaacgctag

4741 ggaagctata gatagtgtct tggattgtag cttgctcaga gaccttccca atgctcggtt

4801 ggaagatttg tatgagatgg agcaagtcga accagaagaa aacaaatcag gattgggtag

4861 ctacacagta gaatctttac agaagtatca tgacgaaatg ttccccaatt gttcgtataa

4921 cattacagaa ctggacacaa cgcaagtcta tttgaacgat ttagaagtgt acaccgaaaa

4981 catgactcat actaatcatt ctgagattat gcaggaacct gaatttgaca cagtgaagag

5041 ttccctatct actatggccc ctatcgttag accattagtt aaaaaggaaa gtgtcatagc

5101 atattccaag cggaatttta tggttccgga tgggcagttc acagtagatt ctcacaagaa

5161 agccgaagaa ataatgggag ccattaggaa gtacacgtac agaaaagatt atcaaaaact

5221 cttggattac tatcaacaac atccggttag aacaagtgat ttagaaataa gtcagtggtt

5281 agaagaccaa gaaaccaata tcgtaaacgc gatatactct gataaggcct tctgggaaga

5341 aggtctggat gtataccacg caggattgaa gagaatgcca aaagcaaatt taacagaaga

5401 tgcatggttg aaatatccgg cgttgcagac gatagtattc catcccaaga gattcaatgc

5461 agtgttttgt cccattttta aggaatgcaa gaacagaata aggaatctta tggcagaaga

5521 gacagtattc ttttcggata tgtcgcccaa agaattagga gaattaataa ccaggaaagg

5581 accgtgcctt aatggaatga ccggagaagg cgacattagt aagtttgatg tctgtcaaca

5641 acttgaatcg gctttagtcg attattatgc tatgagagac gcaggtctgg atccgttttt

5701 ggcactactg tggttttggg cacacactgt aggaatctgt aaagatttca aaaataagat

5761 aagattcatg acatcatggc aaagaaaatc aggagatgct tggactttgc caggaaattc

5821 ttgctacatg atcgggtgtg ttttgcgagc ccttgaaatt ttacaggaaa aatacaaaaa

5881 gatctataaa aagaagaaag caatggatcc aaagttggct agaaaattcc tccttaagca

5941 tggggaattc atgagagcgg atttgcgtaa gttggttaag ttaatgagtg gagacgacca

6001 ttatatcttt ggtataagat ttccagagga aattaattcc gtagtgaaag atctgtttaa

6061 cttggacatg aaagtgtttg attatgattc acactacttt tgttcaaagt tcgtcatcag

6121 aaacccaatc tcactcaact ggtacgctct tccagatcct gttaagctgt tggtaaagtt

6181 ggggagacaa gatcttagaa atcaagaaca taaaatggat tttagaaata gtttgagaga

6241 tttagttaag gaatatgtac atctaggtaa cttcgaagtt ttaaattccg ctacatctga

6301 taggtacaac cttaaaacac ctggtttggc tcaggctctg tactcagtga ttaacgatga

6361 tggtgagttt gacaggttgt ttcctattga tccggctgct aattatacta aagatcccag

6421 tcgtcctaga gagtaagtat gtcaaagatc ttcatccgta tatagccaac ttttcacaga

6481 ttcattcatt gttggtagga aggagagcgt atctagaaga cttcaacttt ctccagcgaa

6541 acataattat ggacgtaaaa ccccatagat atgtgatgca ggacagagat gaatggaaca

6601 tagataattg tattaaattc atagaaactc attcacttat tcccgatgat tgtcaatcta

6661 ttttgggaga cgaatgcgaa ggagcaacct tcaaaagtat agaaacgaaa tggcatggtc

6721 tgcaattggg catatgtcat ccaacaccca attgtacgtt ccaagatttt gatcaatctt

6781 atcaatgcgg caacgcaaaa tatgtattgt atcacgaata cactttcgat atcccatatg

6841 ttgtattcgt tccagttaaa aggaataaca aggtaacgtg gacggccatt ttggaatata

6901 atgaggaaga tctctcaaag tatcagtcac cgggttcatt ttcattaaaa tcacacatgc

6961 aacaaagttc ttattatatc aagcaattat tcgacaggag tctacccgac gatgtcaaat

7021 atgagaccca gattcaacaa accgaatgcg tggaatcact acctttttac gacgctgatg

7081 gtttttatca tgaaaatata gcttatctta atcatgattg gaaaaggatg tgtttctcgc

7141 ttgcaccaca aggttcgtat tgcgattcct tgtggtgggt cgatgactat tttgacaaaa

7201 caactttagt caaagcattg ttggaagaga ttccttggta tatcgtctac catgactgga

7261 attcactaaa acagtctatt aaagatcaag tcaaaactga ttgtgaaata gaaaatgaca

7321 tacatttgcg atggaatgta acacattgtt accaaggaga ttttaaatgt atgtatggac

7381 catttccctg tagcgattca gcgaatagag tcagtaagtg tttggatgtc gaagaacatt

7441 tcacttctcc cgtcaccttt cacgcagtga aagtggttcg ggatgttgtg aaagcaatat

7501 tgccttacat taagaccatc ttcaccacgg ttttaggaat catagtagat gttttcaaca

7561 gtgtattagt ccaacttgga ttggtattta tgttgctcac aactgcaagt attcttgtaa

7621 tggtaataaa attcggtctt cgatacttag attcatcatt aatattatta actttgactc

7681 tactcttatc gactctggga atagatctta aaaattcata tttcatcagt tgcatattgg

7741 tattaacagc aagtaagtat agtaaagttt tagaaagata tgtaaaggaa aattaaaatt

7801 ttgtttttaa aatggcaaag aaatatcaga ccagacgcac ccgcctcaca gcggtgggca

7861 ctgctaggaa ggcgattcgc aagactaaaa ccaagtctcc caagactcaa gttttagtgg

7921 tttccaatgg taagaggaag tccaggaagc gagagaataa gaaactagta ggggtagaag

7981 gtgcttatca ctcagttgta aaagtgctta atgaacccta cacacttatt ctcattgggt

8041 tagctttgtt ggtaatcgtt aactaccaaa actccaaaac ggataatgtt gtcaccaagt

8101 tggctgacac cattggaaac caaaccagcc tggggatatg gatgaaggca aatgtggcca

8161 aagtcatagg cttggccatc atggcgcctt ctgttttcac atctccgaaa agtattagag

8221 taccattagc cataggctct cttgcactcg tctacctagt caaggctttg tcattatgga

8281 attatttctc aattgcaatc gcaattagag tgtttttcaa agttaaagat caaaacataa

8341 gattaatagt ttttggatta gctttcgcga tgtattatat aggagcaagt taaaataaat

8401 attttctttc aaaatggaca tggagaccct agctcaaaag tttgagaata tgcaaggaca

8461 gatcgttgag ggaatgagtt attctcattt tctcaccgat tttcagtcat ataaggacac

8521 tttagagaaa gctcaaaaca tctcaaatag tgtcgtggtt attcgagggc cgtacacggt

8581 tttattcagt cgagatggat ttgaatcaga catgtaccct ctcagaacca aaattcaaga

8641 acaaaacact tttaagttct cgaatcctga agtaaaatca atatcagtta ttcattatga

8701 ggacctccat ttactaattc atgttgaaat ggaaaagttc gatacattcg tccgaggaca

8761 tttggtttgg ggagaaccac attgcgttgg atattatacc tctctattta tacgaggaat

8821 aagaaacaga ggctttgagt tgtatgaaca taccgtatta tccagaactt ataccttaga

8881 tcagttgata aattttggag agtacaaagt aggttccaaa ctagaaaaag ccgaagaaag

8941 atccatccca ttttggagag ggaaatccca atcaccaatg gcagaattac atgaagttat

9001 tcccaaagaa ttcttttatg tatcgaccca ccgatccaac tccgggggtt tttcaacaac

9061 aatcacagtt cctaaactaa atgtcttttc acaaggagaa gctcaaaata agagaaactc

9121 aagacaaatc gcagcttcgc gcttgttaaa gaaggtcaga gaagttaagt ttgaagaagt

9181 cttaaaatgc gcctgatttt gatattcgcc ctaatcacct ttgttcattc aaaggacctt

9241 aacaacacca agatcgtgtt ttgggaagat ggaaatgaat atctttatcg agaatctata

9301 caatataacc cagtctctcc tagatcttgc gactatagga gagttgctta taatctttta

9361 gaaacatcca agaacgagta tttggtattt tacttctgta aaaataaaga tcacaaagaa

9421 gtaaggaaaa ttactcgttc gcttaaacgt cccgcagccg aattgacaac tcggtcgagc

9481 attttaaata ttgctcgtta tgttagcgga gaataacata aaagtcttgt cggttaccgt

9541 taagactgtc agtgttcggc ttacaactag tccgaaccct gtcagcctta aaggttattg

9601 atccgacccg cagtcacata tgtggcggtc gatgcgacca ttctgagcgg agaatcagaa

9661 tatgcaatag tgagacattc agtgtttggc cccaaacaac tgatgggccg ttcactgatt

9721 gtatcactag gaagcctaaa ctcccgcttt ctgaagcttt ttcattttgt tttttgctta

9781 aagtcattta tcttattcta gcttttttgt ttatgttgtg ttttgccaat catatgaggc

9841 tgtctcttat

# Hubei virga-like virus 21

**NC_033192**

1 gaacaaaagc ataaagcccc ataattcatc aatactactt tttgcgtgtg attacaccgt

61 tccaagtgtt tgatgaattc cggaattcgc tgattattgc gtgtgattac accgttctaa

121 tctcgttttt cggctcgctt tcgccaacgc cttatttcaa ctagcttgtt tcaactagat

181 tgatcttact cgccaacgct cacttaacac tgtgtgatta caccattgtt aatcctactc

241 gccatcgttg cagtcaagat gtacgattac cgcaggattg aattccagtt cggatattgt

301 tatatcgcac tgctaaatga cgtctgcgtg cagctggggc ttcctgcaac cgggagaatt

361 ctcctggggg agcgcgcttt ggcaagcctg gggcggtttc cttccgtccc atctttgctg

421 aaattcctta ccgaacttct tccatatgcc cagcgcgttg aggttgcgtc gctgtttcat

481 gtggggggaa tgaccctgca ctacccaggc aggagacgca tgatgctccc ggatttcgtg

541 gagtattata cgtgcttcaa atcctatagt gtagggtccc tcaccgttca gcccggtaaa

601 ttgatgcagg acttgggcgt ttctccgtcg gacgtgacaa ctagtgcact caaagcggtc

661 gtaccgatgg aggccgccac taaccttgtg gctgccacta ttgcggggga agtggacaag

721 tgtgcgcgta tccacaaatc ggatcgccac gtccgcattc ctttcagacc gactcgcgtg

781 ttgcaacggg tgatcgaaac tagttacgat cattacaaca tcgagttcgc tgggggcatg

841 actcagcatg cgtacgccgc tgcgtcacgt attctcgagg tcgaagaaat gctccacaag

901 ttgaactaca gcaacaacaa accgcctcgc cgattcgatg ctcatatcat tgatgtcggt

961 ggtaactgga tgtcgcattt ggacaaagga aggaaatata tacattgtga ttgtccgatc

1021 cttggggcca atgacgctcg tcgcgcagcg gatcgtttaa ctcgtttgcg catgcgccac

1081 ggtgctacat tgaaccaagt ccgtattctc aagaatgcgg ctgataaggt gcagtactat

1141 gatcgttctt gcccaatgtt ctgcatgaac aaaggcggtg cctgcacggt ttctgcacct

1201 gcctgcatgt ttgtgcattc agtatatgac atgaccggtc gggacattgc ggacgcaatg

1261 gattcgcacc aaagttatat cgggtatggt accttcattt ttaatccgga cattttggcc

1321 tcaatggatc tggacaccgc cggagacata ccggtgtatg acgcgcgctg gaagatcgag

1381 atgcacaata aggtgaagta cattcgcttc tcctttaggg atgattcatc ttaccattac

1441 gtgcacagac tcgatgacta cctagagctg gtgtgcaagt ctcacatcgt ctcgtcgcgc

1501 ggagttacat atatggtgga gatgcttaac aacgtcaact ccacccaatt cttccgattg

1561 actagaattg atatcactac ctgctttccg gaacaaattc accacagctg gtggttgcct

1621 gttgacgacg ctgtcaatgt gacgtattac gtcttagatg atgctgcgtt caagttcggt

1681 tatcgccgac ttctcaagcg gaacatgata gtctctggat cgttcctgac gccgctgatg

1741 cagtacgcct gccgcgccag cgagtcgaaa tttacagttt ctgacattta cggatttgcg

1801 tgctcgctgg cctcacgcgt agtcattaac ggtaacgtcg tggtcgcgcg caatgccgat

1861 tttgcaaagg atacggactt tcgtttgctg gatctcgttc aatttgtgta cattcgaacg

1921 tttgagacca agtacagtag tggaattgta ctgcagaaaa tcatgcaaga gataaagaag

1981 gagaggttgc gaagtgaggg cggtttcctc atgaccctat gggatgcggc gaagacgaaa

2041 atcttgggga agatggagaa ttggtcgaat tcgctttgca actttttcct tggtgcactg

2101 gttaaaaggc agtacggtct agagtacgga gtgatcgatc gcgaaataac tttcagcagt

2161 ctcatacacg ttcgcaagac ggtgctcgct cgcagagaag gggtgactca taatgaattg

2221 gacgccgtgg ctgatgtcac cggtgtctat ttcagtgaga aagccgtcat ggatgatctg

2281 ctcggcggta agaagtctga cgtgctatgt ggaacagggc gcaattcgtg tctcgaaaac

2341 cacaaggagt ttgtgcaggt cggtgtgccg ggggacgggg actgccagtt ccacgcgatc

2401 gcattagcgt cgcgaagatg gctcgacgga aaagccgtac ggacgttctt tgcgggattg

2461 cccggtgcac cgaactgcct gctaaaaggg gattgggggg acgacgaatc actcgcgttc

2521 atatctgcta aagcgggtat gcgtttctgc gtgcacatga ctggcacggc agctcagtac

2581 gaacattttc gcaccttcgg ggatcacggg caggtctacc acttgtctta caacgggtct

2641 cattatgacg cgttgttgca gttatcggaa atcgaaccga tgactccagt cggaccagcg

2701 acagtggaga cgtctgaagg aggtccgccg cacagcgtcc tggactacct gcaccgcaag

2761 ctgttggggc aggatgttgc ccagtatctc cgagaaggtg acgatccagc cgtgcggagc

2821 gccttggagt tccgtctgcg catgctcaac ttattcgctc gaaacgcaga caagatgccg

2881 aaaatacttc gtacgaatgt ctcttacgaa gagctcttta agtctttagg tgctgggaaa

2941 ttcaagatcg cgtgtgggaa acgagtctac atgcatgtct ttgacaaacc attgtaccac

3001 agaactgaaa gagtcggtgc tgatcttgaa cacgaggaag cttttcccgg gatgtcgggc

3061 acagagcagg agaagtcgga gcgattgtat atgaaagatt tcgtatcggt cgctcgtgat

3121 atcgcccaca tgtgtgtcaa acaacccgtg aacaagacca tcacgtccgc gaccccatta

3181 ccgactgtcg cggaagcgcc cgcgcaggat acccaagcac agaccggtcg tgcccagagc

3241 actcaagcgc ccgttcccgt cgtggtagcg cagcccaccg ttcaggacac ccagaaagag

3301 gtgaggactg actctacccc gtctgagcca cggcactctc gcccggtttc gcgcgcaact

3361 gcaagatcgc gtagcggttc tgtgaccagc gtcacttccg aatcatcctc ggtgaccgtt

3421 gttacgacaa ccggggaatc tgtcagggaa attagaaccg tcttggctac gaacgcggtg

3481 agtgatgtca tcaccgcgga acgatcgtac tacccggata tacccttcag ttgcgtggat

3541 aggcagcatg cccgtgtact tgacatactt acgcagagtt gtgtgtatgg tgagacctgc

3601 ttagacctca gcaatggtca aggcggtagt gcaattgcgc tcgctggatt attccagaga

3661 gtttacgcgg ttcatgacac cgcgcaacag catcagcaag cagtgctgcc gcagatcact

3721 cacctgcaac tcacagaaag taactcgatc gagacgaccg agttcgtgag cgcgctgtgt

3781 gatagggtca ccagtgtcga attgctgctg tacgattacc atactgaagt cgaatcgata

3841 cccctcgagg acacggttct cccgaaaata tcggtggttg cgtccattgc cacaaggctt

3901 gttaagactg gtggggtact cattttgaag tgttacgatc tgcttaacac cgccactcgc

3961 agcgttatta aggcgttagc ggcgcacttc gagactgttg agtatctcta ctcggagcac

4021 gccccgcctt tcacgggcgc ggtgtttctt gtctttaagc aacgccgtga tctgttgcaa

4081 atggacatta ccgttcaaac ccgcgctgtt cgcgaaaaga tgcgcgggtt gtcctccaag

4141 tcgctcgggc agttgcgaaa gttgaccgag gacgtttacg cagttgtggc taggtccatt

4201 gccggaggag gaactaagcg catgcccacc gttgccaaac ccggtaagca gtgtaatgat

4261 ccaaacgaac tgcacaagcg ggatcaggtc atcgcgatct gtcccgagac ctaccagttc

4321 tgttgtacgg acgacattgg ggtgcgcgaa agtgtgttac ccccgcccgg actaggcatt

4381 gtcgcaatac gtggaaaacc tttcaaggta gacaagcgtc aacatctcat ggtcatccac

4441 ttccgcaaca cggatgaact gctgagttac attcaaaagt tcttcgtgaa catgaatgtg

4501 cgagttgcag tggttggggg tagtaaaggg gattttccgg cagtatcaga gcacctgtat

4561 atcgttcatc gcgataaccg gcagatcact accacccatg cctgtgcagc tggtggcggt

4621 ctcgaatggt actggtccca aaccaacgct tacaagcagt actgggacaa ggccgacggt

4681 gagtatgaag ttgacttcag catcccgcca agacgcgaga cgttctgtcg caacgctgtt

4741 cgcgaatgta gagaacagtg gtatatctcg ctgaagaatg tcggacagaa gtacggttcg

4801 ttctacaagt ggcacatcga taacattgct tcagtaccac cagaagacgt tcgcgggatc

4861 aagaagctgt gccaagacag tggtgaagac ttcggtatca tcaagaacgg tgagttcatc

4921 gttagaccga tggacatcga gttttacgaa aaggcttatg acggtgagcg atttatcgat

4981 ctgacgtatc cagacaacga tcccatgtgc ccccgcgccg atcatgtcgg ttatctactg

5041 gtcggcaagt cttctaggct catgcaaggc gcgagcatgt tagaggcgac tagggatttc

5101 gatccctacg gtacctatat cccaccaata gccctgcgaa atggtgtccc gggctgtgga

5161 aagacaaagt acatcatcga caacgcggag caagcggact acatcctgac gaccactagg

5221 gagaacaagc aggacatcgt ttctagatgt ccgacaatgc gttctcgcgt gagaaccgtc

5281 cactccgtca tcattaacag caagacggtt gagaacacgt cggttaggag attgttcatt

5341 gatgaggcgt tgatgtcaca tgctggtgag ttgctcattg cgataacaat cctgcgccca

5401 gagtctgttg aaatgagcgg tgacgttaat caaataccgt tcatcaatcg cgcggctgct

5461 atcataatga agtttgacga tgcggctcgc atatgtgact ccatcactca cgctagcgtg

5521 tcatatcgag ttccgaagga cgtagcggcc ctattcagct cttcgtatga gcaaggtttt

5581 accactaaca acaagatcga gagttcaatg aagtgggtcg aggtaactgg ttacaatgaa

5641 ttacccaagg ccgatcccgt cctagttttc aagcaggctg agaaagctat gttgcggctt

5701 gaagggtatg acgtgtcgac tgttcacgaa tatcagggta aacagtcgca gaagatctcg

5761 ttgtacagac attcgactat accatcggac caaatctata tgtctgatcc gcacatattg

5821 gtcgcactct caaggcacac acagagcctg gtttattaca ccagactcaa agataaggtt

5881 tgtgacgtca tcgataaggc cgttggagaa ctggagaagg ttcagcaacg cagcatgtcc

5941 ggtggtggtc cgtcgtgcgc aatgatcatg aacaccaatt acgcgggacc aaagtacaca

6001 aagatactcg aatacgggtt agcctatgat gtccctcgtt ataggttgtt caaatccgtg

6061 gcatcgatcc tacgaccgag acacaccaga atcaggttga acaacgttac accgcatgtt

6121 gagattttgc agcagtggta tgataccatt ctgccgacaa tcagcaccgc cgacagaacg

6181 tttgataacc atatgataca taacgacccg ttgtccgtgt caattgctgg aaaagtgacc

6241 ctggatctgt cgaaactgaa atacgacggt cgtaaatttg acaacaagag accggttctg

6301 cgtacaggaa ttggcttgga gcgtattaga tcccagcgtg aatctctact agcctacatc

6361 aaacgcaacg acgcggtccc gatcccgctc gaaccagtcg acccaacgta cgtcgtgaat

6421 ctcatgatgg agaagttcca aggctacttc gacccggaga gacttgaagg tgttctcggt

6481 gtacccttaa ccctcaattc tgagtcaatg accaagtggg cattggccca ggataagagc

6541 atagacacga cagtcgatca gtatttgcac gagcaggatc tgagcagata cgaattcatg

6601 atcaagcctc gtccaaaacc ggatttgacc aaacttgcta acagcactta tgccgcgtta

6661 caaaccatag cctatcaacc gggaaagatc aaccaatttc tctgcccgtt gataaaagac

6721 atgaaggaga gaatcttgtt ctgtcttcgc gatagattca agatcttttc cgatgtgacc

6781 attgaggagt tcgcagctaa agtgacagac ctgtttcctg atgggtttga tccgaattcg

6841 ttaatttacg aattcgacat ttctaagttt gacaagtcac agaacgaaat cgctctcatg

6901 ctagacgccg cgatcatgag gatgttcggt ataaacgaag aaattgttca gctatggata

6961 agcggacaca ccgccactac actggtggat tacaaggggg gtctgaaggc ggaggtaact

7021 taccaacgca aatctggcga cccgttcacg tttctcggta atactctgtt cctcatgtcg

7081 tgtctggcag tcatcgtgcc gctcgagcag atagagttcg ctgcttttgg tggtgatgat

7141 cagatcatcg tcactaagac cgacattggt ctcagctccg tgcaatatct cgagaatgtc

7201 ttcaatctcg aggcgaaact tttcgtgcgg aaatatccgt atttctgttc gaagtttctg

7261 ctacatgcag gagaccggtg gtattttcta ccagacttgc taaaactggt gaccaagcta

7321 ggaagacacg accttcgtaa cgacacccat atagaggagt acagagtctc gttgaacgac

7381 ctgttgcaag tgtacaggga caagacagtt tttcctgtct tcaatcaggc gttcaatgaa

7441 cgctacccga gcccgatcgt cgaccatacc tacattatcg aagtggtttt agctctatgc

7501 gagagggagt tgtcatttcg gtcgctgttt tacagcagac ccgatgacat cgtgtgtaga

7561 gacccgcaga gacctaagtt taagggggag taggtgggta gttaaccctt tattcttaga

7621 ttaattgtta gtgtaacatt atggttaacg gtgcatttcg cagatcgcgc gctcgtgcta

7681 cggtggcccg gcgttcgttt ctcgactctc tgctggtggt gctgttgcgt gttgttgcgc

7741 accccgtgtc gttggtttta gctgtctttc ttgttctttt tgtcgcagct gaagttttag

7801 agactaccgg tcctctggag tctctagaca agctgattaa acaggagttg ggctccaaag

7861 acatcaacag tcttgaaaag tttctcctga agggctttga caagtgtata gtatttgtca

7921 tcttgtacaa aaccaaagta gtcgccactc tcgcctattc catatttgtt gccttaaatc

7981 cgacaaaact acgctggtct gtctttggtg ctgcagtttt gatagtcgtt gcgatacctt

8041 cgcttcccgt cttctatcat atcatcactg cagtcgcgct agtgttctat ctcgcgcttc

8101 agcgaattga acacaaggct ctcacggtgt tcatctatgt cgctggaatg gtgttgtata

8161 ctagcgctgt tgtcgtgtcg gttgcgacag ctggtgctgg taagcgcaac tccactggtg

8221 caccgtagga tacagccctt ggtgacgttc tcgggggtgg agcccgagta cactttgctc

8281 gtcgaccatc ttccggtggt cactgagggc gagaagtgtc ttgtcgcagt agccggtgac

8341 tcgtgtgagc tgccggacta ttgtggtcaa gtcgagaggt ttcacatgaa ggttcaggat

8401 ctgctacttg tcatctgtgc gggtcatcat gtgttacctg ggcacaaatc ggatatcgaa

8461 atttttagag cctactcatt ggatgcccgc ggtgagttta ttcccgttga attatatttc

8521 atcgacgttt actcattgga caggaagtac cgttattcgg tcgtgcttcc gtgggcgatg

8581 ctgtctaaat tggtcgtcaa gacagctgtg caacacagtt tttatgtgga caaggatctt

8641 cggatctgtc gccgtggtac gtatttaagg tctctgacca gactactgtc acgtagaatt

8701 cttaattcgg aaaagcaact ctaccctggt gacgcgcctt ttcttgaagc gaattcactt

8761 cggggagcat catgccgccc gtccggtaca cacgaaccct attcctgcac tgatctcata

8821 gtcgttaatg agacctatgc attgtgctcg tcggtttgga cgcaattgca tcccggtgcg

8881 tgcccgatgg atcggcaata tcgtcgtggt gaattcggtt tcgaagatgt ttgtactaac

8941 atcacagtcc tggagaagaa gaaggctttc ggtgaagatc cagacgacgg gtggcttcaa

9001 agatctttga aacaagtcgt caattggttg accacgaaga ttgatgactt tgccgagttt

9061 atcgaggggt tgtttttgaa actactcgag aaagttatag cttttatgtt ttcacagtta

9121 gaagttcttg attccctggt ggaatttgtg gactcgcggt atgtagtgtt tgagctaatg

9181 gttgtatcgt tcatcatctg ttatagatcc aacctacctg cagctttgat tttcgtagtt

9241 atcttcggag tcacctgcgg ttacgatcgg acacgtgatt tcagactcct cccagaactc

9301 aaagctctgt tgctatggtc gggataaatc tattatgtta aatgtttcgc ctaagcatca

9361 taccaattaa ttacagattt gattatttcg agttttcaat taaattaaat tgggtcacta

9421 cttgttccga atattaaagt gaagttacca tgagtttcat caaggatttg ttcgcccgct

9481 tcctggagtt gttccgcggt agtatctcga atgagatcgt ccgtgaacta gtccgcttgc

9541 agcgggagcg tgcaattacc atcgctcgca ccgtccttac ggacgtgtac gtttacgatg

9601 gtaaggcgta cttccggact gccgaagtga gacgtcatga gaacatttac tatcaatata

9661 aggatggaaa atgtgcagtg ctggaggagt caatccagaa ggttgttcgt acctgctctg

9721 cgtactctcg tttcacgagt gatttaatcg tcgaggacga tattggagtt ttagttccgc

9781 ttgaggtttg ctttatcaaa actcaagcga acgttgatct tgcactttcg ccggcgaatt

9841 tggaagatcg tgttgcagcg atgcgtaaat tcgttcgcga ttacgacgct gctcactcac

9901 caccggcccc agctcagggt gtggtgagtc atccgccgag acccgcatct cgtaagatgt

9961 gagtctcgcc ggagagccga cggttgaata taggtaccta gtaccgggcc gagacgacga

10021 ccgttgtata ccagtctcaa ttcaatataa attaaagtaa gactatggca ag
