# Supplementary material for: RNA helicase domains of viral origin in proteins of insect retrotransposons: possible source for evolutionary advantages
Source: PeerJ. 2017 Aug 16;5:e3673. doi: 10.7717/peerj.3673 (PMC5563155; doi:10.7717/peerj.3673)
Supplement: Supplemental Information 3 [file peerj-05-3673-s003.doc]

**Multiple sequence alignment of the analyzed SF1H proteins encoded by Lepidoptera TRAS-like LINEs and Jockey-like LINEs of three other insect orders**

Andesiana_lamellata NIRWVNGVPGCGKTTWVVKHF----DEEKDVVATTTTEAAKDLREKLAHRLGDR-----V-KTKVRTMASILVNGFKK---QEKCYRLTVDEALMNHFGT

Biston_suppressaria KMQWVNGVPGCGKTTWVIRHF----EVDKDLIITTTTEAAKDLREKLSHRIGNL-----V-KTRVRTMASALVNGFGK----EGFSRLMVDEALMNHFGA

Caloptilia_triadicae NFTWINGVPGCGKTTWVIDNI----DYKDDAVITTTLEAASDLTNRLAGRIGAV-----S-KSLVQTMASVLVNGLKA----PRRKRLFVDEALMNHFGA

Ceuthophilus_sp TITLVQGVPGCGKTTNILSK-A---T-SNDLILFATRESAEAFKIKYQEKHQAKNQ-KKINMEHIRTLHSFLINSHKL--QNHNYYRVIIDEALMAHCGE

Eudarcia_simulatricella RINWVNGVPGCGKTTWVIGHF----EAATDVVITTTTEAAKDLRERLALRLGAD-----A-TSKVRTMASMLVHGLRGR-DKDKCKRLIIDEALMNHFGA

Gerris_buenoi IIKWIDGVPGCGKTHYIVNEH----TPGQDLVLTQTRANLRDIKSSVEKNIKTKDN-RRI-DRDYRTVSSFIINGSD-----KDYQRVFIDEAVLMHAGY

Homalodisca_vitripennis ELELVQGVLGCGKTTFIINK-H---T-KGDLVLFPTREGATDFRNRLKQKDQTASY-NNI-KDSCRMIHSFIINSTNHIKSGGKYNRLIIDEALMLHTGE

Leptopilina_boulardi V--WVNGPPGCGKTHYIMQNHVLPHKGGSDLVLCTTSDGVSEFRKRAEAKGVPKV---FL-NTYYRTVQSVLVNGCDK-----QFAVVWMDEFLMSHAGV

Lyssa_zampa KFTWINGVPGCGKTTWLVSRF----DANEDVVVTTTTEAAKDLLEKLTRRIGKA-----A-KSKVRTLASVLVNGFREP-DKRRCNRLMVDEALMNHFGA

Nilaparvata_lugens KIKWYDGVPGCGKSYFIVSHH----EPGKDLVLTQTRAGIKAIRETVIERYGRKHC-NRL-KLDYRTVGSYIINHNQ----NKTYNRVFIDEALLMHAGY

Ostrinia_nubilalis TITWVNGVPGCGKTTWVMSQI----DTSRDIIVTTTCEAAKDLREKLEPKIGAR-----A-KKRVRTMASLLVNGMSE---GETCTRIMVDEALMNHFGS

Planococcus_citri YFKMVQGVPGCGKTTYILNNLH---YLSLDLVLFPTREAAIDFRSRYCAQFGVPIDTAWL-KYRFRTIDSYLMGTDK-----NIYKRLFIDEASMIHFGQ

Plutella_xylostella TIAWVNGVPGCGKTTWVVNNF----DVSKDTIITTTTEAAVDIRNRLAHRIGDM-----V-RTRVRTMASVLVNGFRE---HVGCQRLIIDEALMNHFGA

Polyommatus_icarus NYSWVNGVPGCGKTTWIINNF----NEETDVIITTTIEAAEDLKQRLSLRTGNK-----V-KDKVRTMASLLVNGTKG-----AYKRLIVDEALMNHFGS

Sindbis_virus TI-GVIGTPGSGKSAIIKS-T----VTARDLVTSGKKENCREIEADVLRLRGMQ-----I---TSKTVDSVMLNGCHK-----AVEVLYVDEAFACHAGA

Tischeria_quercitella SIYWINGVPGGGKTRWIITQF----KVGTDVIITSTTQSAADLKEKLSQRVGPN-----A-RSSVRTMASILVNGLRG---QGTCRRLIIDEALMNHFGS

Andesiana_lamellata IVMA-VKLSGASEIVLIGDVNQLPFLDRENLFKLRYTR-PN-LVAGITQELHCTHRNPMDVAFALSEI-YS--GIYSSKSSLSRVHSLKVKGYTGA--QI

Biston_suppressaria IVMV-SRLSGAGEIVLIGDVNQLPYIDRENLFEMRYHR-PN-LVTKISQELLCTHRNPMDVAYALREI-YS--GMYSSVC---RIKSLEQKRYKGA--QI

Caloptilia_triadicae IVMA-SKLAKVEEVVLIGDVNQLPFIERENLFPVTYHR-PL--HFGIEKDLLCTHRNPMVVAFALRKI-YG--GMYSPKR---QVRSLAKKGYRRS--TV

Ceuthophilus_sp LLLS-AYTAGCKELELFGDQNQIRYINRTTHCTVRYADI-L-QITDKQTCHNTSYRCTNSVAAILSSY-YGNNGMKSIST---VKNEMEIRTYVSA--TQ

Eudarcia_simulatricella VVMA-ARLAGASEVTLIGDKNQLPYIDRENLFTMKYDR-PN-LVAQITRELLCTHRNPMDVAYALSEV-YN--GIYSTSP---KVKSLTLKTYKDA--HI

Gerris_buenoi VGFI-AELANAKEIILLGDANQIPYIERSALISE-WSNI-A-RFCSPSKTLSVSKRCPMDVCFLLQGY-YK--EILTVNT---KVHSIR-PPIADG--SF

Homalodisca_vitripennis VLFA-CALAGVKKALLVGDKQQIPFINRTTCNMIHYD-I-T-KIAKTTTVLNFSYRCTNSVTTLLSPY-YEQ-GMATCNS---VENEV-DSVYLDDL-NK

Leptopilina_boulardi LGAV-TLLTGAKKVVCLGDKNQIPYIDRDHVSDLKYTL-PS-SYMDVREELHASYWCPIDVMYVLSPM-YSSLPIYTNNL---LVRSMVWKRYSENFSGI

Lyssa_zampa IVMA-ARLAEAKEVLLIGDINQLPYIDRENLFPLFYYR-PT-QLTTISQNLLCTHRNPMDVAYALREV-YD--GIYSSVM---CVNSLKRGMYKGV--NI

Nilaparvata_lugens IGFI-ANLSKASEIIVVGDANQIPYIERSNYATR-WHKI-S-EFCEPFTKQTVTRRCPIDVCFVLSTV-YE--NITTLNE---RATSIL-PTYRNG--EY

Ostrinia_nubilalis IVMA-VQIAQASEALLIGDNNQLPYIDRNNLFPLLYNR-PN-LITNVTKELLCTYRNPQDVAYALREI-YS--GIYSAKT---LTRSLQLKGFTGA--KI

Planococcus_citri IVYA-ICKSSARIVIMIGDTHQIPYINRSPIPAIFHSSIPD-ALINETEYLSTSYRCTTTSSFLLCHL-YKH-GMFTTST---VRREMKLHQYVSI--EQ

Plutella_xylostella IVIA-ARLSRASDIALIGDINQLPYIDRENLFELRYSR-PT-LVANITQELLCSYRNPMDVAYALREV-YS--GIYAATT---RIQSLQLKRFTDA--AI

Polyommatus_icarus IVMA-NQLTGANDVILIGDINQLPFIERENLFKLNYTR-PN-LVTGITQELSCTHRSPMDVAYALSMV-YN--NIYSSKE---IVRSLKLTKYTGA--RI

Sindbis_virus LLALIAIVRPRKKVVLCGDPMQCGFFNMMQLK-VHFNH-PEKDICTKTFYKYISRRCTQPVTAIVSTLHYDGK-MKTTNP---CKKNIEI-DITG--ATK

Tischeria_quercitella IVMI-VRILQAEELLLIGDVNQLPFIDRDNLFKIKYHR-LP-ITPHKHQELHCTHRNPVDVAYALQNI-YD--GIYSSST---TIKSLNITNFTGA--NI

Andesiana_lamellata PSTAQ-NTLFLVHTQE--EKASLISQGYG-SGEGSRTLTIHEAQGLTYDSVIIINTKSRRLQIHD-SISHAVVAVSRHTVSCVYY

Biston_suppressaria PNTLP-NTLFLVHTQE--EKETLTNQGYG-SGTGSRILTIHEAQGLTYESVIVIKTKA-NMKLHE-SVPHAVVAISRHTGNFTYY

Caloptilia_triadicae PEDLP-NTLYLVHTQA--EKAALLSLGYE-KGEYSRLLTIHEAQGLTYEDVVILNTVEKKMRIHD-SVSHAVVAISRHTRSCTYY

Ceuthophilus_sp IQIPK-EAQVLTFTQS--EKMELVHAGY-------KAMTIHEFQGKQAYTIVLIRTSNYNSEIYN-SIPHCIVGISRHTKKFIYY

Eudarcia_simulatricella PADSP-NTLFLTHTQA--EKELLKSEGFG-SGDKSRILTIHEAQGLTYESVVVIRVADRRTQLHD-SVPHAVVAVSRHTLRCVYY

Gerris_buenoi YQLKP-DTLILTFTQN--EKAMVTRCLENRCSEPLLVHTIHEAQGLTSKRVILIRINTSPLEIYN-SIPHVIVALSRHTHSFRYL

Homalodisca_vitripennis LHLNKSLFKVLVFKQA--EKRSLNSLGL-------NASTIHEFQGKQASHVAVVRVNRTKDNIYD-SIPHCPVAISRHTKVFRYY

Leptopilina_boulardi RRDQD-YTLYLVHNQS--DKQQLVDNKYG-IGKGSAVLTIHEAQGATYEHVICIKKNEKPLMIYD-QVPWAIVAISRHTKSLVYY

Lyssa_zampa PKTLS-ETLYLVYTQD--EKISLTNQGYG-SGEGSRLLTIHEAQGLTYKRVIIVNTMDKKLQLHD-SVAHAVVAISRHTIECVYH

Nilaparvata_lugens HLIQP-DTLILTFTQE--EKLMVGDT-MK-WREDVALHTIHEAQGLTHKNVILIRIKYKENEIYN-SMPHAIVALSRHTETFRYL

Ostrinia_nubilalis PN-QE-DTLYLVHTQA--EKALLIGQGYG-TKTGSRTLTIHEAQGLTFREVVIVRTTSKKSHLLQ-SVPHAVVAISRHTDSCTYY

Planococcus_citri LPHIK-EAHYLTFTQQ--EKAFLAKHGF-------PTNTIHEFQGKQNGHIIVVRFIKDKAAIFE-SLPHIIVGLTRHREKLDYY

Plutella_xylostella PKSQT-NTLFLTHTQE--EKETLTSQGFG-EGTGSRVLTIHEAQGLTYESVIIIKTKD-KIKLHD-SIPHAVVALSRHTSACTYY

Polyommatus_icarus PKTDL-NTLYLVHTQE--EKAALTNTGYG-SGTDSRVLTIHEAQGLTSPSVIIIQTKSRKLAIHD-SVPHAVVAISRHTNTCVYY

Sindbis_virus PKP---GDIILTCFRGWVKQLQIDY-------PGHEVMTAAASQGLTRKGVYAVRQKVNENPLYAITSEHVNVLLTRTEDRLVWK

Tischeria_quercitella SRTQE-NTLYLFHTQA--EKESFKAEGYA-SGEGSLILTIHEAQGLSYKTVYIIKSLRKHTQIHN-SVSHAVVAISRHTESCTYF
