# Supplementary material for: RNA helicase domains of viral origin in proteins of insect retrotransposons: possible source for evolutionary advantages
Source: PeerJ. 2017 Aug 16;5:e3673. doi: 10.7717/peerj.3673 (PMC5563155; doi:10.7717/peerj.3673)
Supplement: Supplemental Information 4 [file peerj-05-3673-s004.doc]

**Nucleotide sequences of genomic loci of four analyzed retrotransposons**

***Ceuthophilus* sp.**

**GAUX01000930**

1 cctacatgtc aaggtcttac aaatcattaa atcttgaatt gcttaaaatt aaatggatat

61 aaaaataata cattgcctga tgaaacccta ataaatcaaa taataatttg acaaaaataa

121 ttgaaccaag aacagcgtat cacataattt atatctgatg catcccacta gttattttct

181 tcaggagcaa tgagggattg atcataaagg cagtggggag gttaggcacc acgttaaccc

241 aaaactatga ttctaaatgt ttgataatct tgtaacaaaa gtatttcatt gactaattca

301 tatgataatt tagataacat tttgtattgt tcaacatgga ccaatgttac atttcagaaa

361 ttaaaaggaa aatataattt actgtaatac attatggttt atgtattgca aatgacaaaa

421 gagccaatct tctctgctgg caatagcacc aacaactttt taacaaatct ggttagatat

481 tgataatttg cattaagtca gtttaatgga aagtgatggc gaaatatttg atcagtgaaa

541 caacttctat ttattctaga attaaaaata aagtaaacct cacaatcacc accttgaagt

601 cataggacaa gggtggttat aaaatagttt tctgaccttg taaaatgagt acatatgcag

661 tgccttaaaa acaatatata attgaatgaa gacattttat tctatatctt aaatgcttga

721 aaaaatagga acttaggatt ccatatttga actgaaaatg tttattcaac atttcactga

781 caaaactgta tgcaagataa acctgtacta tgcgtcaaga ctctggctcc tccatagtgc

841 cgtagttaag agcccagtct cctaacccat agttcaactt ccactgtaga taagttgaaa

901 gcaaacagag cccactccat cacctattga gtgacagctg gtggccagtt tggtcatcta

961 caaaagtacc cggtttaatc ccccactccc cttagtaaat gataggaaaa atctacttaa

1021 gtttaccata caaattaaga cataaataaa tctgagattt aaatgctatc acttttaatc

1081 caagcaaaca tgatgtgata tcgtgttgca taaatatatt agaataacct atcgtccaac

1141 aacacagcat aacttatgca ggtacaaaac tttatttcaa atactgtatt tctccgcata

1201 atcgttgcag attttatgtc aaatttgttt acgaaaaagt ggggtgcgac cattacatga

1261 agctttttat tttaaaaaat taatttaata caaaccattc tctatatttc ttgcccaaca

1321 accaccaagc taaacccaaa aacaatgttt ttcgcaggtc aaaataaaga taaactgttt

1381 tcactcacca tatgattaaa taacttacaa ttgtatttta tgacaattta tttaaaatct

1441 gtttacacgg ttgttgagag taagaataaa tatgtcttta aaaaattgaa acatcatagc

1501 attaaaattg tgggctgtgt agcaatgtat ttgaaaataa tttcaaagta ctgaaaactg

1561 tgatgtcaac ttcaaatgtg tgtttaggat gtttgttgtg ctaaatgtaa acaatcaaca

1621 gtgtgaggtg cagaaaagta taatctttgc attgggatgc ataaacatac aataattaga

1681 caattactgg tactttttgt cataaacaaa acttggcaaa tgctcctgtt gtgctgattg

1741 acttgtttaa aatatcaggt ttggcgagca gtatagtcat gtacgtggat aatcaaagtt

1801 attacaaata tatattaacg aaagagtttg taaataactt atatattaag aaaagatttt

1861 gtaaataacg tgactagaac gtatgagatt ttccatatgc tgtttactta agtcaaattg

1921 tacattgtcg tctacattgt tttgatgcat tgaccactca agcaccaagt gtacaataca

1981 atacaataca atacaataca atgggcttta ttcatgaaca ttgagtacag aattggtgtc

2041 atattaaaga ttattattat ttgttatatt aacaattagt caagaattag tcaagtgaat

2101 atagtgttcc ttcagtcaac ttctttcgtc tctccaccca aggacctctt ctatggagta

2161 gaatgactgc ccgagcaacc agtcttccaa ttttcctttt aaagctgtga ggttctctgt

2221 tcccttgatt tcttgcggta ggatgttgaa aagctttgct cctatgtatg aaggcttttt

2281 ttcatacagg gcatggctgt gagcagggat gctgaaattg ttcgcatttc tggttttgtg

2341 ttgatggaca ttacgtaatc tcactagtcc ctggttcact gcatacatca ccacagcact

2401 gacatataga gccactactg tttgaatctt tagatcttta aatgttgtac ggcaagtctc

2461 tctctgatca agttctgcta gtactcgcag ggctttcttc tgtagcacta acactttgtt

2521 taggttgcct cttgacgagt acccccaggc aactagccca tatcttagat gtgattcgaa

2581 cagtgcaaag tatgctgttt tagctgtatg taggtcactt atcttcttta ttctttttat

2641 cacgtatagt gcagtgctca gtcttttaac aagcagctgg tgtgtggaga ccatgaaagt

2701 ttgtcatcta tgatcattcc caggtatttt gcttcacttt ctacagtaac atctggcagt

2761 cctttgctat ttatggcttt ttttccgaat attagttgtt ttgttttatt ttcattaagg

2821 actaaatcat tgttatggca gtattgtttg gccatgttta acactatgta agctccaatg

2881 tctaactcct cattggtttt tcgtcctatc attagtgcag tgtcatctgc atacatgagg

2941 gttgagcagt attcctgtag atgttctggt agatcgcttg agaataagat aaagagtacg

3001 ggtcctaaca ccgatccctg tgggacacct ctagttgttg gtaggtggcg cgatctgact

3061 ctgtatagtg tattgttaga actatgcgtt atttctgtaa gctgtgtcct gttttgcaga

3121 tagctagtga accatttgca tgttgtatct ctaataccta gcgttttaag tttattgatt

3181 aatagttcat ggcccagaca atcaaatgcc ttactaaagt caagtagtat tccagtgact

3241 gcatttccct cttccagttg atcaattagg tactccacta gttggatgat agctgtagta

3301 gttgattttc ctttggtgaa gccgtgctgc tttgttgtta ataagttatg ttctgaaaga

3361 tgggctagaa gtcgttttag ggctattttc tctattacct tagagattgt tggggtaatg

3421 gagattggcc tgtagtttcc tgcttctttg ttatttcccc ctttgtacat aggtagcacc

3481 ttagcaagtt tgagattaga agggaatatg ccttgtctga acgatttgtt gattatgtca

3541 actagtgggg gtgttagctc ttctttgcat agctttagga tcttggacga catatcatct

3601 attccggcag attttttatc ctttagagaa gttattatgc gtacaacttc attttgagtt

3661 gtaggatgta ggatgaggtc cggtatattt ctatcttccc tttgtaacaa ggtaggtttt

3721 tttcggtttg aaggttgtcc tgatctttgt atagtttgtt cggctacatt ggtgaagtat

3781 acattcatat gctcagctat ttctccagga tctgttgttg ttacaccctg gtcgattagg

3841 ctagtgatag tgttggcatt gttagcttcc tttctttcat tatttatcac ttgccatata

3901 gctttagctc tattttctga accatgaata tattccaccg tggcttgtcg tctctgttct

3961 ttgagcctta gatcataatc ttttttcctg gctgctgttt caactttcct ctcttctcca

4021 ccttccaggt tttcgttgtg tagtgcatca agataagcca gtttaagtct tctagcttct

4081 tcatcatcaa tgtttcgtct ttttccttta ctcttgggtc tggttctggt gtatggacag

4141 gctatgttta gtgcattttg atagattctg ttgaaagtgt tatatgcctc atcagcatta

4201 tctgtttcta ggatacagtc ccagcattgt tcagaaagga tgtattttag ttgtcctagg

4261 ttgtcctcat tcaaaattct ccttgttgag ttgagtcctt ttactacttt ggtctgctgt

4321 ttgatagtac atatttgggc tttgtggtcc gatagacctg tgatgatgac attaacttca

4381 agttcggttt ctgggatgtt tgtgcataca caatcgatag atgatttagt attgtgtgtg

4441 actcttgttg gcggaagatc ccgcctgtgc atattacagc tgattagtgt gttgttaagc

4501 ttttcattat cactgtcatc cttaaggctg tctacattta tatcacccat gattattata

4561 gggctggtcc aggttggtat gccatctatg atgttagcaa ttatgtctag cgctgtgtct

4621 aggtttccac ttggtggtct gtaaattccc atgatatata tgtatttgct tcctgtctta

4681 attcggagta ggtgtgtttc acatgtgagt tcttgctgtg tggtttcaag atattctatt

4741 tcatttccca gattcttgtt gatatagatt attactccac cctttctatg attttctctt

4801 gcaaaacctc caacaagtga gtagtcagga aggcgtgtgt tttccatgtg gtgtttctta

4861 agcccgtgct ctgttaatat taccaggctt ggtttgtaat caatgagtat gtgatttagt

4921 ctttctattt tatttgctaa cccatctatg ttttgatgta ggatggtgag gagtctttta

4981 tctacattgt gcatcttttg tgttttttcc cttttgtatg gtgtgatcct aaaaaatagt

5041 tatctgtgtc ttcttcctgt tctatgtttc cattcttttt gcagtttgta ttttcctcca

5101 tttgcatatc ttctgtcatt tctgctgtgt ttccttgact gtatgtcagt ggttcttgca

5161 gatggtttac tagtagttga gttgcaggtt gtagacgtat tgctgtgtat ttaggcattt

5221 ctatgtgttt attgaaaaaa ctttcatatg tttcacctac ttccagtagt ctggcagtgt

5281 atggtggttg acgtttgaat gtttttattg ttgacctatg cgcgggcgag ggagttttca

5341 tactattatg ttctacgata tcggcattag tttctgtgtc atctagactg ctgggcagct

5401 gtggttcgtt agatcctgtt ttattattat atgggtggtt tgttaatatt ccttctggtc

5461 gatgtagctg tttgttgaca ctgggttgcc cagattccaa gtgcccatct ttatccttga

5521 cacgaatatc cttttgtttt tttgctgagt gggcttgagt ttcttggcgg gttgatcctt

5581 gatgtttctt gtttgaattt gtatagtatt tcaccctgtt tcgtacactt gatgtatcta

5641 ttcttcctgc ggcattcttc tgcagtctcc gttcccctgg tttcgaagga agttcagcga

5701 tgaactgtgt ttgatattca gcttgatctt cagaaaggtg taaccgctcc gagtcatcag

5761 taaggtaggg cacacaatct tccctatttg ccctattgtc agtgttgtag gagtcttttg

5821 ctttagcatc gctatgttgc ctgttggtgt ttatataata ttttgttgca tgccagtcat

5881 ttgatgtgtc catttcttct gtagtgtctt tctgcagctt ccgtttttcc tgagcacctc

5941 gtataggcgc gccggtgttt ttgctgtttg gtacctgcac gtttggaact tgttctgggg

6001 tggtgtttgt tattgttcct gctgctgtgg gtagtgaggt tcccactgga ttagacagag

6061 aagcagggga ttctactagt gtttctgcat actctactat ctcatttctt attgtagatg

6121 taacaacctc tgatgacgtg ctgtatattc tattttcttc ttctatgtct ctttcttctt

6181 cttttttatc atctgcctca taaggattta tattatatgt tgtcattaca gataaatgtc

6241 cgtccacatc ttctaggagt accgattcaa cttcttcctc cagtgattgt ttatgttttg

6301 attctttaac atggttattt aataggcttt catatgtttc attttgtttt aataatctgg

6361 ttgtatacgg tggtttacat attctcttcc ccactagcct aatgtcctgc cgcacttctg

6421 caatttgctc tacaactgct gattcagtca tgagggggca ggcggtatta agggagtttc

6481 ttggtgaatt tttagatgag tcttccattc cttcaattac gtccatttct acaacatttt

6541 cagctaggtg aaccagctca atttcttctt ccgttatttg tttttttctt agttcatcta

6601 tgaattttat atatatgctt tcatatgttt cgtgattgtc tagtaatcta gatgtacacg

6661 gtggtttact tgttctcctt ttatttactg gcatactatg atgcatttcc tcaatttgta

6721 tgacatctgg gggtttgagt ggtaaactac tggaagaatt ttgtggttta ggctttgatt

6781 tgttttgtga ttctgcctca tcatttattc cctttctcgc gatctcttcc ttatcttgtt

6841 gttgatttcg tattgctacc aaatcatagt ttttctctat ttgagcatct agcctttcct

6901 gttgtgtaaa gttttcaatg tgcgagttta gaatgctttc atatgtctcg ttttgtttta

6961 atcttctagc cgtacgaggg ggtctgtttg atgttattaa atcatcatcc tcttgagatc

7021 ttggctgtac ttcctcgtgt gcttctataa aattagaagt tttatctgca tcattttctc

7081 cttttatccc ttgtctattg gtatatttat tgttgttgga aaagtatcta gtttgatagt

7141 aagtcctagc accactttcc agttgtatgc aagatgtaat atgatgggcg ttgactttgt

7201 tctttatatt cgtacagttg tgcattgtat tttcactgtt agttgttaaa ttttcccttt

7261 gtggttgaga gtttaccttc tgtctatagg ctgttgtttg taattctagt tgggggaagt

7321 tgattatttc atttgtttta cttgtttgta atttgtgttc ttccttctgt ccattggata

7381 gagttgatga ttccacctgg attatagtgt gtgcatctct atcatcactt gattgagcac

7441 ttgtagattg gttctcaaca gttgggattt cagatttttt attattatct ggagcaacta

7501 ggtaattgca taagtccatt gttttaccct tgtttataaa tttggccatt tggtctaagg

7561 gatccgtggt gtagtagatg aatttcttgg tatgccttga aatccccact atacaatgcg

7621 gtatgctgtt gtagatctca ctgttatagt tagatgttct tattaacact atggtatatg

7681 cttgctttcc ttgaaattca tggatagtca tagctttgta tcccgcatgc acaagctcca

7741 ttttttcact ctgagtgaat gtgagcactt gtgcttcttt aggtatctgt atttgtgtag

7801 ctgagacata tgttcttatc tccatttcat tcttcactgt ggagatactt ttcataccat

7861 tatttccata atatgaggac agtatggcag ccactgaatt cgtgcatcta taacttgtgt

7921 tgtgacaagt ttgtttatcc gtgatttgaa ggatatctgc atatcttaca gtacaatgag

7981 tggttctgtt aatatatctg atttggtttt gatcaccaaa tagttctagt tctttgcaac

8041 ctgcagtgta agcactcaat agtaattcac cacagtgtgc cattagtgcc tcatctatta

8101 taactctgta ataattgtga ttctgtagtt tatgggaatt tattaggaat gagtgtaatg

8161 ttctaatgtg ttccatattt attttctttt gatttttagc ttgatgtttt tcctggtatt

8221 tgatcttaaa tgcttcagca ctttctctgg ttgcaaacag aattaggtcg ttgcttgtgg

8281 ctttactaag tatattagtg gttttgccac accctggtac tccctgaact agtgtgattg

8341 ttggcagttt gaatttttcg acatttaggt cttttgtggc ctcgtaaagg gtaagatcat

8401 ttagcagttg agtctcatcg ctaatcagca caatgttatt gcaatggatt acatcagggt

8461 cagctaatac gtatccaact ccagattttt gtatctctac tagtcttgct ccatcacatc

8521 catatttgta gttcattttg gcggcggggt gtttgtatat ccactttcca gtcgatacat

8581 ccgctataca gtagtgatgg tgtgatgcaa gtaggcagtc tcttgttttt tgattgattg

8641 ccgttatgga gttcagtttg tgagtgcgat agaaattact aagttccttg agagttattt

8701 tttcttgtac tctccagagt tcaatttgtt catacatagc atttattgca aagtgtttat

8761 tatcacttac cgagatgtga ctaggatcga acccacaagg tggtggatta gcactactta

8821 gtgattgcca ataatcgagt ggtttgatat ctagttgacc aaatcttctt ccaatatttt

8881 gatagctttt aaagatcttg tttgttcttt tttgttctgc ttgtggcact atgatctgga

8941 atctgtgatg atatgattgg cagttaaaat tgtcatatat ctctttgacc tgattcaacc

9001 agtttgggtt tgtgggtaat agtatagatt ccatgttaat atagtctaaa ctaatcagca

9061 tggacaagtt caagcaggag tttatcaggt ccagaaacct gtgtaattga gcttcccagt

9121 tatgacccgt ggtcgagtta gtcatattaa ggtacaagtc caaagtatgc agattgttac

9181 catcagctgt aacggtaaag agtccatgcg acaatgttgt tttagcttgg taatggttat

9241 gaatgcataa cttaactttg atgtcatttt caggccttag cgtgatcaca acaagaggag

9301 tttctctagg atcaaagtta gaagctttaa gtttttctag tgattttaca tcattatccg

9361 ttataggcag tttatcatcc aattgataaa ctcgtacttt gtcaatactt cgggtttgtc

9421 taatttcata tacagtatca gcattaagtt gaactgtttt ctgtgctatg ctactttgct

9481 ccggtaattt aggccagtta atgttatggt ttggctgatt gatgatggtt tcaatcagtt

9541 cttgattttc tagttccatt ttttgtgtgt tttttacatg tgttgcatgc attttctcta

9601 gtgtttgaat atactgacat agttctgttt tttcggcttc tttctcaact attagttctt

9661 ctatgcagtt gttatttaga ctgatttttt cattagcagt ttttattacc tcttcttttt

9721 catttgttag tttaattatt atctctctag cttgtttgat ttgatccctt atttcagaga

9781 tctcctcttg gtgttcattg tttgtcgttg aggtgtcttg aagttcctgt attttatgta

9841 ttagtagttt attttccgct agtaatacaa tactgttctc tgcagccgtt cttagtgcta

9901 tttcgagttc catttctttt tcatctagtt tctcgttatt atttgatctt tcattcagaa

9961 gttccaggtt ttttctctgc gcatatactt taagttcttc agattctgtt tttagtttat

10021 ctatttgttc ttgagcaagt tgtagcttgt cttcaagttc attttgcggt tccgcatatg

10081 tttgttttag tctcatttca tgcaagtctt gttttaggag ggcgttttca tgtaggagtg

10141 catttcctaa ctctgccgct agaggaatcg aattttcatg ttgattttca ttgtcttcta

10201 tgttgtccag atcttttatt ttatctcgaa ttctttcaag gtttaaggca ttttctcgcc

10261 tttcacatcc cacacagagc cattttatag gttctgcttt cctgattttg ttaaagactg

10321 catgcgatat gcctacacat tttatgtggt accatttatt gcatatcccc gtacagttta

10381 tggccgagtt tttacaaaat ttggtacaaa taggacagtc atattgattc attttagatt

10441 tcttaagtta gggtatagta gcacataagc tctttattgt tattgttctt attatgtttg

10501 gtgctgctag gtttgttata agtaagtgta tttactttgg ttgagttgat tattattaat

10561 tatttggata gtagatacta actataatgc aagagttgta tctactagcc tatgtttatt

10621 tacataatgt cctttaatta tgtagtcaat tttaattaca tgttttattg ctattgccta

10681 gagttgtata atccttgagt cagccactgc agtgtgagga gtgcacagtc agcaagcagc

10741 tagtgccaat gtactgggtg ctgagttggc tccaatgtct agcagctgtt atgtatgtgt

10801 ggttgctttc aagatatctt gtaataagat atatgattag tttcagatag tcggatgata

10861 ttttgtagac aatttcttat agtatcgtga gttatatata ttgtggtatc ttctcttagt

10921 tatgtgtcat tttgtgctgc cttataagta ggtcagctct gtgatttttt agttgtagta

10981 tgtaggtatc cttatattga gtattttgta tttataattt atatttatac aaactcacgg

11041 aactgcaggt atgttggtgt ggatgcaaat cttctgtatc cacattttat tcaacatatt

11101 cttcacttgt tcactaggtc agttattttc acaaatcagt tttttttttt tttttacact

11161 tttacacatc aggcattgtc acattcattt acatttcgtc tttactattt aggttactca

11221 ctacattggg tataacaccc agaaacagct ttaatccttc atattttgtg ttaaattggt

11281 gatcgttttc actgttttac tggctagtcg gccattttat gcttgtatca gataaggtga

11341 gtcgttgctg tgttacgagg ctcattcaat gatgcaatca gtctgtatct tatatgagtg

11401 gtgctgagca cggcgtgtga tataacaaaa ccaatgatag cagcacagaa tcttcggata

11461 cagatgattc aattctgtaa gtctgtactt cgcaacactg tagtaaatgt tctcaagtgt

11521 aatattgcga ctataat

***Nilaparvata lugens***

**AOSB01052258**

1 taagagcagg gaaaaagaga gacaggagag ggagagagag agagagagag agagtgagag

61 agagaaagag aggtgaaaaa tagagagaat gagatatata gagagagaga tggattgata

121 gataggatat atagatacat agatagtaat tcagtgagat agtggaggag aaagataaac

181 cgggatgaga aagaaaggga gaacttggat gaggaatata taaaaggatt gattatgtgt

241 caggtactag ttcactaatt ttcaaattta tcagctgtta tcattgttca agacagtttt

301 tctgaatagt agttttagtt ttcctttatc cactgcatag tgctgagaaa gagattttgt

361 cctgttatcg ttctcattct cacgacttga gccatcccct ccgctctagg cgtgtgcaac

421 ccggagctgc ataagtagta cctcaatcag gaggtactgg gttcgattcc cgggctggca

481 actgattttt ggatagtaga ctagttctca tcgaatttcc atctagctgt taaccctgtt

541 gtcaatgtct gcagtagcag aagtcttcgt ggtgatttac agcatttatt taggattttc

601 gttaaataat aacatggaga atcgatagca taagtagata tcccatggta tagggcgttc

661 atgtcgcaac tttcactgtt atcccaagcc gatagctcac gtagttcttt cctatgcagc

721 tgtgtgacac tggtagtctc tcaaattgtg ccgttccaac actctcaccc caacaaaaca

781 gtaaaaaatt gacaagagtc gacagtaatc ggcttgagat aacagtaaaa gttgcgacat

841 aaattcccta taccatggga tatctactta tgctatcgat tctctatgtt attatttaac

901 gaaaatccta aataaatgct gtaaattacc ccgaagactt ctgctacagc aggcattgac

961 aacagggtta acagctagat ggaaattcga tgagaactac tatccaaaaa ttagttgcca

1021 gcccgggaat cgaacccagt aaaaaaaaag cattcattac aaatgctaaa gtatatatga

1081 attagcattt gaatcaatgc attctctatt aatttaattc catctgtttt ctatatctct

1141 ttcacttttc gaaatatccg ctctaaaaga tgtaacattt ctgagaaaac ccattttccc

1201 taaatttttt gctttttttg ctcgtataac ctctaaatat cgatgggaaa aatccatgct

1261 gattttaagc ttatagagca tctaattttc ttcaatttga tgtataattt cacaagtgta

1321 cgcacatccc cacgactgtt gcagcagctt cagtgttgag tgtgagatct tcagttatgc

1381 aaaaatagac caattgacaa aggaatttgg agagaatgtt ttgaacacaa tttttgactt

1441 cgccgctttg ttgtgaccac tttttgtgta gttgagaagt tgatattgtg gtaattattc

1501 atattgaatg aaaaagacta aggaattgtc aaaaaaccac tgatttattg ataatcagga

1561 agaccggttt cggttattac accattgtca atctctgata aactgttcat cagacaatgg

1621 tgtaataacc gaaaccggtc tttctaatta tcaataaatc agtgtttttt ttgacacttt

1681 cctagtcttt tacattcaat ttgttgggac caattggtgg gtgaacatat caaaagtccc

1741 catccctacc ccttttgcta aaaagatgag ggtggtttaa aagttgcatt ttccaatgtt

1801 ttgcttacac gctcatatct tgagaaaaat gcgtttaacc gacatgacta actgttcaga

1861 aattaagctt gataaattct ctacaatatt tgttctctgg tgaattctga tatctccacc

1921 agttttcgag atatactgat aaattttgtg aaaataataa tattaagaaa ctatgtcaat

1981 ttctagattt caagatattt atctcacctt gggttggcct acttctcggg tagagatgcc

2041 catcttctca tggtgcctca gttgcactgg gtagatgatc tggtagtaat gtgcgcctat

2101 gtttctcacc aactcctgat tttgacgaaa ttccctcagg agtctctcta cttctcctga

2161 aaattgagaa gaaaaaacac aaaatgtgag taaaaatact tcaaattctt gtgtagaaat

2221 tgttgtaatc cagctaaaat ttataacata atagagcaaa tatttagaaa tactcaaatt

2281 tgtgtagaaa ttgctgtaat ccagctaaaa tctataacat aatagagcaa atatttagat

2341 agaagtactt gaatttgtgt agaaatcgct gttatctata acataattaa ggaaatagct

2401 ggctcataca ggtaggggat aggaaattca cgaatgacgc atcatcacgt ctactcaact

2461 actcaactca ttaacttcac attttgcata taaattcttg atttatcgag gatggttata

2521 ggcctattct caattcttca agatttcaat ttgtcaagtt ttcagtttgt ctagttttca

2581 gttatcaata aagatgtggg gtaggtttaa taagacattt tcgatgtgag aatggggaaa

2641 tagtgatcag aatagagaaa ggagcagaga tgaagaaaga tgtgcaggag tagaggggaa

2701 aggagaagaa gaaggagaag aagtagaagg gaaatgagaa ggagcattgg gagtagttga

2761 gggaggagat gatgtaggag gagaatggga agaagaagaa gaaggagagg aaggagaaga

2821 agaaagaggg ggaggataag gaagagcaga aggagttgag gaaaatggag tagaagtgga

2881 ataagtggaa gagaaagaaa gagaaggtga agaaggagta ggaggaggag aaggagcagc

2941 aggaggtgtt gaggtaggag atgaattgaa gaagaaggag aagaagaaga agaagaagaa

3001 gaagaagaag aagaagaaga aaataccatg aataagaagt gagtttcgta gtattggaaa

3061 tacttggttc tcagttttca accagtcagc aatgcttagc tattttctcg ctaagttccc

3121 gacaagttta attgcgagga gagagagcgt gacagggtgt gagagagagg gagtgagaga

3181 gagtgagagt gagaaaacag cttgagtgac tgtcgaagaa gaagaagaag aagaagaaga

3241 agaaggagaa gaagtagaag gaggaggatg atgagcagga gtgatagaga gagaagaaga

3301 aactcgaagg tgtatctttc gaaagagaat atttcgagag aaaatgagaa agaaaaactt

3361 cgtgattgat ggggaagaag aagacaagaa gaaggagaag gagaagaggg agaagaagag

3421 ggtgaggaga gggtagtaga aggaaataaa gaggaggagg aagagagtca gaaggatgag

3481 gagaaggagg accatcttag agatttctcc aattaaactt ctctgaaccc ttatctaatc

3541 attgtctcga tctctctccg attgtttatc ttcctctcac tgcacgttac tctgcgcttg

3601 cttctggttt ttcctctttt catactcctc ctttccccct ccatcttgtt cttcttcttt

3661 ttctccatct ccttgtgctt cttcttcttc tcctactcct ccatcttctt ctccggattt

3721 ctcttctctt ccttatccat aataattttt caatatcctc taataccata atattttcca

3781 cttcttccac ttctatttct tcttttctct tttcttttgc ttcttcttct tcttctccat

3841 cttcttctcc ttcttcttct tcttctccat ctcctcctga ttcctcgtct tcttctcttc

3901 aaaaatcatt ctattaataa ttttcaataa tattacccct tcaatcctac atgattctcc

3961 ttcttcttct tctcctccac accattctac tctcttctct ctcttccttt cttctccctc

4021 ctatagtgag atccacttca caatggcagt ggagaaagat aggagaacaa cgttgccgaa

4081 cctctgtctt gtcaatgcct tctatagacg gtagctgata caggattatt gatgtgatat

4141 ttcaactgtt cattctcgtt tataataatc aattattata ttttattaag caagaaattg

4201 tatttctcaa taatcctgta atcaattctc ataactgaga agagatattt tgttaattaa

4261 ttattaattc tacattgttg aaagatgata tgggaacaga gcaaagcgag agagagagat

4321 agcgctcttc gctttgttga gtgatagaca aggatagcaa taccattgct aatcaaacac

4381 tgccattata acgtggacct cactatatca aatccactat agtgagattc acgttataat

4441 ggcagtgaag aaagatagga gaacaacgtt gccaagtctc tccattttgc cactgactgt

4501 acacagctgt tactcaattc atccattaaa tttaatccaa taataattat catttccttg

4561 ataaaataat caatttgatg tcaaattgat aaataagata tattttttta taatttgatc

4621 caaaaatccg cttttataac tgagatcaga tttttatttc tttacaaacc ttgaatagcg

4681 gctaatttaa aaagctgtga tacaacagtc tggattccaa aacaacagaa ataaagttat

4741 ttggtacgta ttctattcca acttctttta aaaatgatag agaaatagaa atccttcttg

4801 aaataagtaa tttcaatgga aataattctg aaaaaatatt tcaatattat ttataccggt

4861 atgagtaggt ctaacctaaa cgtgtaggct aactgaagct tggatgaaat ctaggatggt

4921 tagttatcaa cttttaaaat gtcgtttcag gtatttcaat ttgtgtttct catacggtaa

4981 tgtttaaaaa ttatttcatt gtttcaaaaa tacatttttc gccacactgc acagaaagca

5041 gctgttttcc agtccctacg tagatctgaa agacattgtt tacagacaac tcttgtctga

5101 cgtcagaaaa gggtttcttt caagcctagg ccgaaaaacg tactcttttt gtagccgcta

5161 acttggaagt ccgtagttaa taagtcatac gctagatcgg gtgagtgtcc actttcttca

5221 tcaaagtcgc catgatttca gttcgagttt cgaatcaaac taattcagca atcgctttgc

5281 aaccattttc attcaattat ttattgttga tcagtacatt ccaaatttcc caaattccag

5341 atacttgaag atgacgacgc ctgtgatatt ccaagtgaaa ttttaaaaga atgcaaatct

5401 tctaccacta aaatcaagag ataggtacaa taacaggtat tatttatttt gtattctcca

5461 ttcattttgt cagcaatacc tctagtaagg caagaataat atcgttcgca ccatgggcaa

5521 aaattttttt ccggctcaca atcttttcta gtcttgaaaa ccgatttcga gccggaaaaa

5581 tctcattttc tgctctaggt gcgaattata ctattatatc aattatacga cttgtgtact

5641 caagtatttt gatagaatga aaaaaaatgg cggctgggaa ttttttgtac agttacaact

5701 gtcaaaagtg aatataaaat attcttgcaa atagacaaca ttgcaaagcg agagagagat

5761 agtgctatcc gcttcgttga gtgatagaca tggatagaaa tatcattggt aatcaaacac

5821 tgccattata acgtggacct cactatatca attctactag cacatcgagc ttataaacat

5881 aattatcgtt gcctccccat gattatcttc aagataatga tgatgataat caaataaagt

5941 tacaatctac tttcatcttc aatttctgag ccaattctca aatttgaaat aatttcaaat

6001 attgaagagt gagaataact ttgaactgtg agcagtgttg ttcgaatggg aagccttctg

6061 ttatctatat aaggtatgct gacggttgaa agtgaatgtt atcatagaac atcagatttt

6121 acgcgtgata tctcatctca gattctaatc tctgtcaatt tcaatcacag attaattgtg

6181 tttgatctcc gattaagaga cgatattctc tgtagaacag agtcgactct cttctattct

6241 gattttcact tcttcagtct cttattctct ctctctccct ctccaattca gtcttaatca

6301 gacctccgta gagtgcagat cgtagcgaat aattagtgta gagtagtact agtagtgaaa

6361 gtggaacaag tggtgtcaac aacaaccagc agtctactca gcagtttttc atagtgaaaa

6421 ctactcaacg tttttttctg agtgaaaagt gtgtcaccaa atttttcgtt tgaacgttgc

6481 tcgctttaaa aattaggttc tgtttcttta ataaacaatt atcaaactat caataaacta

6541 tggtgttttg gatctatcag ccttaagatg atccattcgc caataaaact accaccatcc

6601 gcgcagctag aatacgagga agaggaggaa aaggatgaaa tgaatatgcc tcccgaattg

6661 ccaccgaaaa ttaaaaacga tagcggaata cgtttcgagg tacgttctct tagaaatata

6721 caagagaaat ttctaaaatg ggggagtgaa attatcatga aaaaaaaagt atccagagag

6781 gactatgatt cgtttttaaa tgtgtgcaat gaactaaaca taagtatcga aagaatagac

6841 gtgaagcaaa atattcaaga aaacaacgcg gaaatgtttg gaaaaattca cactctggag

6901 cagagtgtga ataaactagc aagtgaagtt gcaaaaagcc acaacaagcc gatctcattt

6961 gctgaggccg tgcaactgcc aaagaagagg accacacctt ctacattgtc gagtgccgcc

7021 tccactgctg cagccaggga gtctccgccc cggaccaaga agctgttacc caaggagaat

7081 gtgatcatcc tgaagccagc aaaaataaat ggaacggaaa aggaacaaag cggaaaaatt

7141 cgagaaacaa taaagaagaa catcacccgt gaacaaaatt tgaacattaa aaaagccgtt

7201 gatgtccgtg gtgggggtgt gctgttagtg cttcacccaa gggccaataa agaaaatgtt

7261 ttgggtgata aggtcctgca acatccaaac ataaaggtaa gcgagccaca atccaaattg

7321 ccaagaataa ttctgtatca cgtcgctgca gatataactg ctgctgaatt ggcaaacgat

7381 gcatttgaga gaaatctcga gtcatcgtca ttaagtaggg aaaatttttt gaaaaacttc

7441 aggccgattt ttaaaatagg accgaaaaac aaaaacaccg tgcactgggt agtggagtgc

7501 actggtgaat taagaaaaga atttattaac aaatcgcgga ttggtattga ttggagggtg

7561 tgccgagttg gagactatgt tgccgtctct cgttgcttca agtgccaaaa aattggacat

7621 atcagtaagc attgcactca agcacaaaac acctgtgctc actgttccac tacaggacat

7681 gatgttaaag agtgccctaa tagagacaaa aagccctcat gtttaaattg ccgcaatgat

7741 aaaaaagctt tcgatcacaa agtaggtgac agagagtgcc cttcctatca aaaagcgcta

7801 caacaaataa ttgatagaac tgattatgga atctgaaaga aaaatattta atatactgag

7861 gataaataaa aataatagtg atagtaaaat caagcaatca acccaacaca aaatgattac

7921 tttaaattcc atccttaaca aaattgatag tttcaacgta acaactaata ctgataaaag

7981 taagataagt ctggtttata attttggtga gagggttgat gttatgatat cttttacaaa

8041 aataggaaat attattaaaa catccaaagg tctctctctg cttagaaata aaaagtattt

8101 ctctcttgaa gaatggaatc acctgaatgt gttactggat tctaattgta taagagtttt

8161 gttttctgat aagaacattc ctattttcat attaaatgat ggtgaaaatg gggtacttat

8221 tgatcttttg aatagaataa atgattggag taaattaaat taccctttga ttataggaat

8281 tgattctttt aacaatctgg gtgaaactta ttttgatatt cctgccactg aactcttgag

8341 tactgtcata ggagctagag atttgaagtt tattttttat tatcgaggtt attgtgaaat

8401 taattttctt aatataccta agctgtctga tagtccggga gttgagtacc ctgtgggatt

8461 ctttgaaggt aataataaac gtgaagcttt cataaactct atgattgaat tacggaccct

8521 acttcaaaac actgataacg aaaatgctaa aatatataaa aatattcttc ttagggtcac

8581 gcaaagaaac atgtcaatat caggtgatga actaataagc gtcctgaata acaatatgaa

8641 catctatgat aataatttga aaagattcct attcaaaaca aactctaaat accgaatggg

8701 atattgcttg gagcagggtg gaagttacgt agaatttcaa gaggatactt taaaattctc

8761 tactaaagaa cgatatgttt tagtcacacg taatactaca cttatgctga atagcgaatt

8821 aataaaaact ataaatagaa taactcttca tgagtgtaaa cttcccaaaa taaaatggta

8881 tgacggtgtt cccggctgtg gtaaatctta ctttatagtc tcgcatcatg agcctggcaa

8941 ggatctagta ctcactcaaa cacgggcagg tattaaagca atccgtgaaa ctgtcattga

9001 aaggtatggt cgaaaacatt gtaatcgtct taagcttgat tacaggacag ttggctcata

9061 tataataaat cataatcaga ataaaacata caatagagtt tttattgatg aagctctact

9121 tatgcatgcg ggttacatcg gttttattgc taacttgagt aaggcctcag aaataattgt

9181 agttggtgac gcaaaccaaa taccctacat tgagagaagt aattatgcca caagatggca

9241 taaaatttca gaattctgcg aaccttttac aaaacaaacg gtaactcgta gatgtcctat

9301 cgatgtttgt tttgtccttt ccactgtcta tgaaaatatt acgacactta atgaaagagc

9361 tacctcaatt ctgcctactt acagaaatgg ggaataccat ctgatacaac ccgacacttt

9421 gatattaaca ttcacccaag aagaaaaact tatggtgggt gatactatga agtggagaga

9481 ggatgttgca cttcacacaa tccacgaagc acaagggcta actcataaaa atgttattct

9541 aatcagaatt aagtacaagg agaatgaaat ctataacagc atgccccatg cgattgttgc

9601 cttgtctaga cacactgaaa ctttcaggta tttaacaacc agtctggtag atgatgctgt

9661 ggacaaacta attaaaaaac ttaaattcat agatagtgag gggcttgttg aatggaacaa

9721 ggaaaagaga agtggacctg atgatataat ttatgagtaa cactgaagag gttcttaata

9781 ttttagaaac agaaaatgaa acagatgtaa tcgacgatta tttgaactat cactttcacg

9841 aggacagaaa taaagatgct gagtgtttca atattatgtc tatgaatata cgtagtttga

9901 atgctaattt cgatcagttt atttgctgcc tcaatgaatt gaaaactgat attcacatta

9961 taatattaac tgagacctgg ttgactgcag atatgccgtt cattttcaac attcctggat

10021 atacggcaat aaataagtac actaaacaga ataaatgtga tggattatgc atgtacataa

10081 aagattgcat aggattcaat gaagtttcac tcgatatctc tgatgctaat gtagttagtg

10141 ctgacttaag aattgatgat tttattgtga aggtgattgg ggtttataga tcaccaagta

10201 atcaaaatat tgataatttt cttaatagtc tgagtaatga aatcagtaaa attcctaatg

10261 gaataaatgt atgtgtggcg ggagacatga atataaatac acattcaact agtgttcctg

10321 ttcaagatta tctgcatatt ttcaatagta atggctataa gtcttacata aatggtgata

10381 cccgagtaac aaccacaaca aattcttgca ttgaccacat tttttataaa aactcaggca

10441 accattctat gtccactaaa ggagttatat ttaggacaac cataactgac cactacgcgg

10501 ttgtactgaa cttggtgaag gtccctatca ataaggataa tagaaacgta ttgataaaca

10561 cattaactag aaccaactat catgcactac tggaacgcat aaggaatgag gattggggag

10621 aaatctatac cgtgaatgga agcattgaca ctattatgga taaatttgtc gatcgtttga

10681 ccagtctcat caatcaatgc aagaaggtaa aagtgatacc taacagactt cgtcccctga

10741 agccctggat atctgaaggc atcataagat caataatcac gcgggacaaa atgcattcta

10801 gactcagaag agatcctaat aacaatatct taaagaatga atataaggct tatcgtaatg

10861 gtttgaataa gatcataaat catgcaaagg aagtctacta taaagggaaa attgaaaatt

10921 gtaataacaa cagcatgaag ttgtggaagt gcataaacga ggttataggt gaggccagta

10981 tcaaggaaaa gagtactgaa cttgatgctc actccctcaa caattatttc acaaatgtgg

11041 gtaaactaca agctcaaact atcaatatcc ctgataatac tggtgatcga tttcctgcaa

11101 tacagatcga taatacgttt tttatgagac caacgtgccc agttgaagtt gaggcaacta

11161 ttaaaaatct aaagaacaac tgcagtcctg gtattgatgg tctaggtaat attacgctga

11221 agaaaatagc caattttata tctcaacctc tcgctttcat ttttaataga tgctttgagg

11281 agggatattt tcccgagcac ctcaaatcag ccaaaataaa acctctattc aaacaaggtg

11341 acccaactaa tccctgtaat tatagaccga ttagtcttat cagcaacctt gctaaaataa

11401 tggagaaact aataaagtca agagttgttt ctttcttgaa tcttaacaaa ataatcaaca

11461 agaaccaatt cggtttccaa aatagtaaaa gtacatctga cgctctaata aaattcatca

11521 atactttatc tgataaaata aactccaata agaaaactat tgcggtcttc ctggatatac

11581 gcaaagcttt tgatacaata cctcataata ctttgttcaa taaactggag tcctatggtt

11641 tcagaggagt ctcgcttaaa ttgttcaaaa gctatctgag taatagaacc cagtccctaa

11701 ccataaatga tcaatctagt gtaactaact taacttctta tggtcttcct caaggtactg

11761 tactttctcc catccttttc atactctatg taaatgactt tttaaattta agacttctaa

11821 actcaactgt gatatccttt gcagatgata ctgcagctat ttttcacgga aattcatgga

11881 atgaagttca tactatagct gaaaataata tgcttttaat taagaagtgg ttagataaga

11941 ataccttaag tttaaatatt gaaaaaacta attacattac tttctctgca aatagagtag

12001 ggcagcctaa cgagaatcaa gatttgagac tccattctca gtgcaattta aacttgggta

12061 attgtgattg tcccaccatt aaaaaagtca ataatactaa gtatttggga attataattg

12121 atgaaaacct taaatgggat gttcatatta aatacatatg tagaaaaatt agatatttat

12181 cctttaaatt ttaccaagtt aacagaattc ttaataagaa ccacctgaaa atgatgtatc

12241 atgctctagt caagtcaatt ctgcagtatg gcatagttgt ttggggaggc tgtttcaact

12301 gtcatattgc tgaattattt gtagcacaaa aactgataat taaaactata ttaaacaaac

12361 ccaggctcta tccaacggat atggttttta gtgattttga ggtcatgact atacgtcaat

12421 tatacataaa aaccgtaatt gagtacttaa taaaatatag atccgatttt cctctcacag

12481 taaactctac tcttaattat tataatctaa gggccactgc taacaaatat gtaacctata

12541 acactaatat tgaatgtata aggaggcaat taatttacat aggtactaaa ataataaacc

12601 tcattccaaa tcactttctc atggacaata agatcagcgg aaaaattaaa ctggatataa

12661 aaaactggac atacagtaat ttcttcttcc atttagatat atgactcgag atttctgctc

12721 cagcattttt ttattttttt tttttcatta gttttttcat ttttcagtgg actcgcttac

12781 ctttatttta agtacctatt cctctgtttt cttccttccc cccatttctc ccttcctttt

12841 ttcctcatta cttctcttct cttctttgcc tgcctattac atgggaaacc atagttggct

12901 aggtatcttc ctctcttttt ttctcttctc caacacaccc aaacactaag cccatggttt

12961 tttatatttg taacatttta ttgtgtttta tttgtttcgt aaatctttgt aattttgttt

13021 tgtcttgttt tgtgtgtttt taataaattg aattgaatta aattgaatat ttccctctca

13081 ctgatgatta tttagcgttg tcttcgacgg ttgagtgtaa gagagggctg gagagagaga

13141 cgccctaact ccgcccttcc aggtgaaaaa agcagatatt ctactgtctt ctctctcctt

13201 ctctctcaaa cattttctcc ctttcactcc tttggtagag agttagtggg aaggatatat

13261 tgaatattct ttccgaagaa tggacattga tatgtccaaa cctccgcaaa tttatgtaca

13321 tgcattacaa tatgattatt atctatagtt attatattac aaattgcttt ttcatatcat

13381 atacagttca acaattattt tcttagtcta tattatgtaa attcatctat aattttgctg

13441 tattgtaagc tattgtatat aagtgtataa gacaatatat attgtgatct acattaataa

13501 agtactcaat caatcaatca attctctctc tttctcgcac gctctctctc ttcctctcta

13561 actttaaaat ttaaaatttt ccaatttaaa atttttagtt tagtcattag ttttgaagtg

13621 gaaattggac tttttgaagt gaaagtgaaa tcttaacctt attctttttt gaaacttcta

13681 tttgttaaag tttgggagaa gacagttttg ggctatgcct gttgtcttct ctcaatccta

13741 ttatatctat tataataagg atctgaaatt gccaatgaaa taaattaata aataaatgaa

13801 taaataatta aatttctctc tctctctgat gattatttag cgttgttttc gagggttgag

13861 tgtgagagag ggccagatac gccctaactc cgccctccca ggtgaaaaaa gcagctgttc

13921 tattttattc tctctctctc cttctctctc acactttttc tctccctctc tctcctttgg

13981 tagagagtta gtgggaaccc aacctcccac ataattgaac ataatcttct aggttattta

14041 gacaaatcag actaaacaat aaaaaaactt ggacaatatc ctgatattca gattacctca

14101 gatttgctag agctatcacc ttccacttct gctttcggaa gtgcttagta aacaactatt

14161 ctcatatata tattgtttat ttttgtgtgt ggcgaaaaat agcgttcgca ccacgggcaa

14221 aaatgttttt tcagctctcg atcttttcta gtgctcggcc tacggcctcg gacttgaaaa

14281 ccgatttcga gccggaaaaa tctcattctc tcctctaggt acgaaatata ctatatcgtt

14341 attctaattg cattcaatct ttattctcat tgatcaatta tttatacttt gtgaatttcg

14401 ttaaataact agcattatag tcatttaatg taaataagtg tataagccag taaatattgt

14461 aacatacaaa aataaagaaa tccaatctat tctaatctct ctctcttctc tcgaagcaca

14521 acacacacac atacacacac acacacacac acacacacac acacacacac acacactcga

14581 tctcactctt tttctgtcgt gtgttgtata ttgtcagtta ttattgattg aaaatataga

14641 aaaaaactgt cattcaatta gataaaccaa tttgattccg caagaaagta agtgagagaa

14701 tgaattaatt ctactctggg aaaatattat tggaatatcg ttgaatagtt atcaactgac

14761 ttcatttttc tccaaatctt ctatttcctt ccaactcaca tccttctcca cctccttttc

14821 ctctctattt tctccttctt cttttcctac tctactatga catcctcttc tacttctact

14881 tctcccactt ctccagaaac atttccaatt tcgttgactc tactctctct gaagaaatgc

14941 agtcatcatc tagagcttcc tttgcacagt ggcagtgtac tcaaagttca aactcgttat

15001 gctaagagag aagagagctt cctttcccta acaactttgt cttctgataa aagcatttcg

15061 gcaacctgag aatcacgata acactcctcc tcctcctcct catcctcctc cacctcatcg

15121 tcctcctcct cctctccatc ctcctcctac aacattattc atcctcctcc tcttcattct

15181 cttatttcta tctcattccc actacatccc cttccacaac aacaactgca cctcctcctt

15241 cttcttcttc tttctccttc ttctgtcttc ttcttcttct tcttcttctt cttcttcttc

15301 ttcttcttct tcttcttctt ctctttattc cacttctact acatctccct cctttatcac

15361 cgcctcctcc acctccccat cctcctccac ctcctccttc accacttccc acatcaatca

15421 tcctcctcct tctcgtcttc ctccttgtcc tactcctact tctcctttcc ctcctacttc

15481 ttctcttcga cttctgctcc tcccaaacta cctcaagtac ttcagaccct tcctcctcat

15541 catgcttctc ttctctattc tcctcattct tctccttcaa ttcttatcct ctcacttctc

15601 cattctctct ccctcctcat ccaccttctc cactcctcgc gctactcctt ctctttctcc

15661 tcctcatcat ctccatttgg tagacagtta gtggggagga tatttttaat attctttccg

15721 aagaatggac attgatatgt ccaaagcacc gccaatttat gtacatgcat aacaatatga

15781 ttattatcta tagttaatat attacaaatt actttttcat atcatataca gttcaataat

15841 tattttctca gtctatatta tgtgaattca tctataattt ttctgtattg taagctattg

15901 tatataagtg tataagccag tatatattgt aatctacata aataaagtac tcaatcaatc

15961 aatcaatcaa tcaatcaatc ttccttctac acaaactcca tcttctcttc caccttttaa

16021 caacttcgcc tcttcgtact tcttctcctc ctcctcctcc tcctcctcct cctcctcatc

16081 cttcttctcc tcatatgctt cctgaccttc acaagtacat cctcaccatt tttctatacc

16141 tcatctttct tctccatctt cttctttata gttttctttt tgtctttctc ttttatctag

16201 ttcttcaaca ctaattattg atctttttct cttttatcta cattttcttc ttcatctact

16261 tcttcttctt ctacttcttc ttcttctcct ccttcttctt cttcttcttc ttcttctcct

16321 tctccttctt cttcttcttc ttcttcttct tcttattctc ctcctccttt ttcttctcct

16381 cttctacttc ttcttcttct ccttcttctt ctccttcttc ttcttcttct tcttctactt

16441 cttcttcttc ttcttcttct tcttcttctt cttcttcttc ttcttcttct tcttcgtcta

16501 cttcttcttc tgcttcgtag atcatctagt tgataatcat tgatcgaagt ttgagcaggc

16561 agagagaagt cgatagcatc atctgccacc caacttacct tcaaaactta ttgaactttc

16621 aacaaagttg gttgactgac tagtaggact tcttcctctc ccacttcttc tcctcctcct

16681 tcacttcatc tccacgttcc tttcctcctc ttctaggtct ctctccacta ccaaacatcc

16741 attcattcaa aatagagaag aacaaatatt gtaatgctaa gagaagacaa aaaggagaca

16801 aaaagtatga aaagataaaa cagtaatgaa aaattgattg atcgattatt attatgcctt

16861 tacaagggtg cagttaggac tcatggtcct ctctttcaca caccactaag aatatgtaag

16921 acaataaagg aaagaagatc cttatgggac tttataacga atgtagaaaa tagtttcaaa

16981 ggaaactaaa atatttgaag agaattgaat cggttgagat tagttcaatt tcctatgtga

17041 tcagatagtc aagtccacgt tataatgtca gtgattagag ataggaaaac gttatagacg

17101 gtagctgatt gatgtaatat aatcagttta aaactatcaa tcatatttca ctagccgtca

17161 ggctcgcttc gcttgccgta tccgtctagc cagggggctt cgccccctgg atccctgact

17221 ggatcgtccg tgaataagat cagcaggctc gcttcgctcg cctgtatttt tcatgtgtag

17281 gacgatcata tgttaggact atccagtcgg gggtccagac taaacgtctg gctaaacgga

17341 gaaaaatggc gagcgaagcg agcctgatgg ctagtaatat aatattccca ggaatagctc

17401 tgattgaagt agcagtgccc aatcagttct tccgcgataa atgcatttca atcttcaact

17461 tgttgccaac ataacaaagt caactcaact taatgccaac ctgacaaaat tattaattta

17521 gttaccagtt aacaactgtt tcgaagaggt actctctcta gattatagtt ctatattaac

17581 atatggtatg aacatttgtc aatgataatt aagagaataa gaagaatata cttgctaaaa

17641 gacaaacttt aaactctcaa aaaccaccct tagagttaaa atattgtgaa aagatttctt

17701 agtgcgcctc taaagggcca actaaactta cctaccaaat ttgaacgttt ttggtccggt

17761 agatttttag ttctgcgagt gagtgagtaa gtcactcaat gtatatattt ttcaatgata

17821 taataaatgt tcataaccag gatggaatat actgttaatc gattattaat tctatattgt

17881 agaaagacaa tctggcaaca gagcaaagcg aggaagagat agcgctatcc gctttttcga

17941 atgaaagaca aggatgacaa taccgttgct aatcgaacac tgccattata acgtgcacct

18001 cactgcagtg aaagtcattc tgaaaattcg ttttttcaca ttttcaaaag ttgttaacag

18061 gaagagagat tgattgattg attgagtact ttatttatgt agattacaat atatactggc

18121 ttatacactt atatacaata gcttacaata cagcaacatt atagatgaat ttacataata

18181 tagattaaga gaataataat tgaactgtat atgatatgaa aaagcaattt agaataatta

18241 tagatgatat tgtaatgcat gtacataaat tggcggagct ttggacatat caatgtccat

18301 tcttcggaaa taatattaaa aatatcctcc ccactaactc tctaccaaaa cgttaatttc

18361 gttatttaaa ctaaggattt atttattaat ggaaatattg taaaagtttt aatcaatatg

18421 aatattgaag agaaaaaaaa a

**Amyelois transitella**

**GDGN01078241**

1 aaaaaaaaaa aaaaaaaaaa aacaccatgt ctgacaattc atcatcgcca tcccccgcgg

61 ctaagtgtcc cgcgacatct gaggacatcg gcgcctccag cagcgctacg gaagggacga

121 cgacggcccc aatttcgccg atcacccgcc agccgctaca cgagggtaag catgaattgg

181 aaacacagaa aacacacaca cacacagact caaacacaac accacctcac acgcaggcgg

241 cgcccgggac cagcaaagcg gccaaactaa taaacatgtt gaggaaggta ggcaaaagcc

301 cacctacagg ccccctcgaa gtgggacgca gctgggcctt ggcgcaatct gccaagaaga

361 agaccaaatc tggagggttg cgcagagccg tagtggttaa gggcgcgcaa gttgctgcca

421 ggagggagca ggtagacgat gctagacagc tgtcggatat ctcgctcgat gcctccctcc

481 tcgaaaccac ccaacccccc aacacccaaa aagaaacaaa tgtaccaccc agcgtgcaga

541 tgcagacgtc taagccagag gagtcaccct ccgcgcaggt tcccgagact cttgagtctc

601 cagacaccaa gggaacccca gaaccgctgg aaacaaccca agcccccccc cggaaagacc

661 attattccat agtgatctcc accacccccg taagggaaga tgaggagtca gaggaactga

721 ctcccgccac aaacagcgaa gagaagaagg agcagctagt ggcatgggag gaggagggac

781 cggaagccag ctggaactgg gacagcgagg agacagattt ctccaaaaga aggcagagca

841 tacttccgcc gataggacca cacacgggag tatgcaagga ccttgcgaca gaggcggccc

901 gggagtacta cctaaagggg aaagaagcca tcgaaagctc caagaccctg aaaagggaac

961 taagagcaac agctgtcgag tgtatgggca acctctacga gatcgtcctc tcactagccg

1021 aatctaggaa caggcaccgc ctgaacttgg agatggaacg gacgagagaa ctaaaaagat

1081 ctgtctgcgt agagagattg caccgcaaag cgctggaaga ccagcaggca aaattcgagg

1141 cgcgaattaa ggacatggcc gactccctca aagggactta tggatcagtc caaagaattc

1201 agagctggct cgatttcgag atggacggag tggtcaccaa atcggtgaag gccgcaaatc

1261 ttgaaatcag ccaccaaaca acagccgaac caccgaataa gccagccaac agcgctaaga

1321 tacacaagcg ccgccccttg aggaggaccc ggactgcacc tgcaagacca gcggggtcca

1381 aaaaagtcat ggaccaccta gaacgactca ccagtaccgt cggatctctg gtaacagaaa

1441 tccactacct taaaggcgac accatcaggg aaaaaggaga gactgctccc caatctcaat

1501 ctccagagca gcagcgacag gaagaaccct cctataaaga gcttaaggag gaagtcattg

1561 agctgcggat ggacatggag cggatgcttc aagaagtccg cagcctcaga cttgtctcga

1621 aggaggagat tggggaggag atccgcgaag taacagctcc cctgatgacc aaagccaata

1681 aaatactgga cggggtcgaa gaggtgaagg acgtaaccca ggctaactct gcacacggca

1741 cagacgcccg ccaaggtttg gggactgagc tggcactggc ggataccgcc gcacacctgg

1801 aagggattct aaacccgatc agggcggaag tgtccgagat cgccacaaac agtcgccgaa

1861 cgatggagtg gtacaactca accataaaaa atgtccccct tcctgccaaa aacccgcagc

1921 tgagcagaac ctatgctgcg gtagcgcgaa gcgtaccacc caagccgacc aaaaacccga

1981 accacacact gattgtctct agcgccgacc ccaacaacac gggcgagaag gttctagagg

2041 caatcacaaa aacactggac tttaaaaaca ccggtgtggt agtggatcga gtgaggaagg

2101 ctcgaaacag caagatcttg ctcagctgcg agaacaagga agacgtaaac cggctcaaac

2161 aacaaataaa gacaaactcg gcgttaaagg ttcaggaggc gaaaccgcag aacccgctcg

2221 taaaggtcaa caacgtgatg gcatacctca aggacgaaga gatggtcgag cacataaagg

2281 cacagaataa aaagttattt gaagacttgc ctggcgacca ccaacacatc cgtctgaggt

2341 accgaaagcg aacgaggaac ccgctacagt gccatgcagt actcgaggta gcacctctac

2401 tccacaagcg catgctagag gcgggggcag tccacatagc gatccaaagg agaaccgtgg

2461 aagaccagtc cccgctaata cagtgcgcca aatgccttgg atacggacat cccaaggcac

2521 tctgccggga gtcggcgcag tattgcaatt actgcggcgg cgcccactct tggcaagagt

2581 gcaaaacaag gctggaggaa ggaccaccaa ggtgcaagaa ctgcaaagac gccaaggcac

2641 agaacgtctt cccgcacatg gcattcagtg acgaatgccc ggagagagaa gtgtgggata

2701 gactagcgcg ttccaagata gcgtattgct aaaggcagcc acatgaatgg acctggcgct

2761 cccggagagg agacagggac gaagctggga attgtacaaa tcaatctcca gcgctccaag

2821 atagccacag ccgagctact caaagtagca gaggaaaagg gaatcgctat cgctttggtg

2881 caggaaccgt acaccggatc cagcggcaga gtgaagcagt accccggcac tagagtaatc

2941 cagtgctcct cgaacatcag cagccgaaac ccggtaaaag cagcgatata cgtattcggg

3001 gaccagttta ggatcacaca tgatccacag ctggtgtccg agacggagtc cgcagcggta

3061 ctggaatcag gcagcttcag gcttggagta gtctcagtat acttcgacgg gacagccgat

3121 ataggtcctt acattgcgcg aaccaaggca gtatgcgaat cgctcaacac gcctaacgta

3181 ttactggcgg gagatgtaaa cgcctggagt cactggtggg gcagcgaatc cgagaacgag

3241 agagggacgg aatactgcgc ttttatcaac gaaatggaat accacatcct caacaccgga

3301 aacactccga ccttcgaagt ctggcgaaga gacgtgctat gcaccagcat agtagacgtg

3361 acagcgtgca gcccttcact gctgggaaag atcaggaatt ggaaagtcga cagagcgctg

3421 atcacatcag accataacgc catcacgtgc acactggagc taggagtgcg gctgcaacat

3481 gcggcagccc ctactaccag agtatacaac accaaaaagg cgaactggtc ggcgttcgac

3541 gagacactcc tgtctttact agcggagagg aacatcacgc ccgccggagt agaagaggta

3601 gtgtgtggag aggaaatgga tatcctgaca gatgcctaca caacagcaat tcgggaagcg

3661 tgcgaaagct cgataccgaa gataggcaaa aagcgcagag gttccccctt cccttggtgg

3721 acggaagaga ttgaacagct caaaagggat ctcgttcgta agaagaagag gatcaagaac

3781 gctgctcccc accggaagga acacgtgctg caagagtaca atgaggccaa ggcgttatac

3841 gccaagcaat ctgccgaggc ccaaactcgg agttggaagg aattctgtac gaagcaggag

3901 aaagagagca tgtgggacgg aatctaccgt gtcctcagga aaacatcagg tagaaaagag

3961 gagctactgc ttagaggacc ggatggaaag actttggatc ccaagcagtc ggcagtgcta

4021 ctagcgaaca ctttctaccc tgacgacacg gtggaaaccg acaccccgca ccacactcaa

4081 ctccggaaaa ttgtacaaga aacacctcaa gagaggttgg gagaattgaa agaggatgac

4141 ccgccattca ccgaagccga actggaggcc gtgctgcaag agcagaaccc caagaaggca

4201 ccgggaccgg acggattcac ctcggacatc tgcgccagag cgatccgctg ctcacgggat

4261 gtgttcatgg cgatagccaa caagtgctta gccatctcct acttccccaa acagtggaaa

4321 atcgcacatg tagtcattct caacaaaccg ggcaaagaag attacaccag cccgaagtcc

4381 tatcgcccta taggcctgtt atcagtgcta gggaaaacgt tagagaagct ttttgtgaga

4441 agactacagt ggcacctact cccaaccctc aatccaaggc agtacggttt cttgccgcaa

4501 cgcggaacag aggacgctct ctatgacctg atcggacacg tcaatgcgga aaaggaagcg

4561 gggagatccg tgttgatcgt gtcactggac atagaggggg ccttcgacaa cgcgtggtgg

4621 ccggctctaa tgaaccagct gaggctcaga cgctgcccca aaaacttata cctgatggtg

4681 gactcatacc tcaaggacag aaagatagtc gtcaactatg cgggagaaac cagcgaaagg

4741 gataccacga aaggatgtgt tcaaggctcc atcgggggcc ccaccttctg gaacattatc

4801 ttggatcccc tcttgcatag gctatcgtca gaggaagttt actgccaggc gttcgcagac

4861 gatggagttc tggtgttctc cggtaagacc gtagagggaa tggaggatcg cgtcaacaga

4921 gtattggaga tgacggtaga gtgggggaag gaaaacaagc tgaacttcgc agcacacaag

4981 acgcaagtca tgttgctgac aaaaaaactg aagtacaccc ccccccatat caccatgtcc

5041 ggaacgactc tgactctggt cgacgagatt aaactcctag gactcaccat agacaggtgg

5101 ctcaacttca acactcacgt gcgaaacatc tgcacaaaag cggccagcat ctacaaacaa

5161 ctgacatgcg ccgcgagagt cacctgggga ctgaacacag aaatcatcag aaccctgtac

5221 gttgcagtga tagaacctat cgtaacgtat ggggcctgcg catgggcaaa agcgtctgag

5281 aacatcacaa acagaaaagc gctagacaca ctccagaggg gtttcgcgca aaggatctgc

5341 aaggcataca gaacaacgtc actgccggca gcgttaatcc tagcgggaat cctaccgctc

5401 gatctcagaa tccaggaagt tgcgtcccta tacgaggcga agaaagggct ctccgtcgat

5461 ttcctaccac cgcatcgtaa actcgaggag attgttaaag ccaaagattt accacacccg

5521 gcttctcaaa tcgaactaga gtacactcta ctcgaggata tgacagatgc cacccaaact

5581 gcccttcaga ttactggccc acaggtgtat accgacggga gtaaaatgga gggcaaggtg

5641 ggagcggcac taacgtggtg ggaaagtggg aaggagatac acagccaagt cttcagcctg

5701 gaccccacgt gtaccgtgtt ccaatctgaa ttgtatgcac tccacagggc ggtgtccaga

5761 gcgctggaga gcgaagagga agcgatcaac atccttagcg actcaagatc gtcgttggaa

5821 ttgttgtgca gtactcggct cacccatcct ctggcaaagg ctatcagaga gagcatagca

5881 gtaatacggt cgcgagggag aagggttcgc ctgttctggc tcagagccca cgtgggcacg

5941 gcaggaaacg aaagggcgga cgagcttgcc aaagagggag cctcgaaaga gaatctctct

6001 cccgattacg cagaagtccc gctgtcatac gtcagaagaa agatcaggga ggaatctatt

6061 gccagatggc aagacagatt cgatactgcc tcccaaggtg cggtaacgag aacgttcttc

6121 cccgatgtaa gtaaagctta cagcttcgtg cgaggaacga atctaaacca cctgcacgtt

6181 caaattctga cgggtcacgg cggcttcgga gaatacttgc accggttcaa gttgaaagac

6241 agtcccggat gcgagtgcga cccgaacgtc agcgaatcgg tatggcacat aatactcgac

6301 tgccccaggt ttcaaactgc ccgttacaac ctggagtgtg tctgcaacca aaagttggga

6361 agggaagtgc tgagcgatct gctccataac aaaagcacaa aggaccactt cctcaaatac

6421 atcgaggaag tcgcagcaat cgcaacaaag cgcaacaagg ccacggctgg cgaggcgcaa

6481 ccctcctcga acacacaaac acaaacacaa acacaaacca cacacacaca agggcagcag

6541 tgctcatttg gcagcgaaga cctattgcca aatctcttgc actcagccga ggccggcaag

6601 cccaggctca gggtccgagg agttgccatg ttcatgaaca acaactcgga acgagtggga

6661 gtggccttct gcaacgacag ggcgaagaca aacgtctaca tctcaccggg actcggtctc

6721 ctattgaacg gaagcacaag caaaactacc atgcgtcgta aaatctacga cgccttacca

6781 acggtatcag tggggggtca gcggtgtcgg atagtgcgga ggaagaacaa aaccatcgca

6841 ctgttcgcca ctgacgacaa ctccacagcc ttccagaagg tatgcagcgt gctgaaggac

6901 atagggaact ggagaacggg agatactcac actcctaaag tgctaagcgt ggacgtcatg

6961 gcagtgtctc accacacggg cgaaacccgg gaccacctgg gtgcccttac agcctcgcaa

7021 caccacgagg tagtggtata cgaaaacaga ggtcaagatc tcggttttct tttgagacgc

7081 ggacagggcg aaagcgaggc cgctgcgaat cgcgggggac ttccgagtca atctcgggac

7141 tccagcggat cggagaggct gcaacaagcg ctcgaggagg agagtcgcca gtcgaagcgg

7201 atagtcaccg accaggacga agataggaag aagtccacgg tcacctcgct cctaaaccga

7261 atggcccagg cgatcgcagg cccagtggga gagaaaatga gcaaactcag aagaacggta

7321 ctacaggaaa cggcagtggc aaaattcaca accccatcga cgtcccgcca agaggttacg

7381 cagaccaccc agcgaaccag agcggacatg ggacttgtga cccccccctg gctgaagccg

7441 gcgaacgatc agctggccca tatggagaac gctctacgag agttcatagc gataacggct

7501 gccacgcgca aagtcaatga agatatctgc agtgcaatat tgcggactta caagcgtggc

7561 aacataaaat cgctcgaagt gaagctagag gaagcggagg ctgcagttta cgacctcgac

7621 gcacaacgtg tgatacacgg gaaggcgtgg ggggagtaca tggcggcata ctgcgcaacc

7681 gagggcttca tagaactcga aaagagccag ccggaagagg agggccacat tcgggccata

7741 aatcctccaa aagacccgaa ggttgtggtc gccaagtgca ccagagtaat gctggaagac

7801 agaatcctcg aaatggctaa aaccatcttc ggggacctga cagaaggcaa caggctggaa

7861 tgcttggcgg taccaacatt cacatgggtg aacggagtgc ccggatgtgg aaaaaccaca

7921 tgggtagtcg ctaatgtcaa catagaaagc gacctcatcg tgaccgccac aagggaagca

7981 gtcaaagatc tcagggacaa actgacaccg aagatcgggg aagaacgagc taaacagcga

8041 gttcgaacca tggcttcact attagtgaac ggaatgaaga agggtgaaac gtgcaaccgc

8101 ctcgtggtag acgaagccct aatgaatcat ttcggatcta tagtaatggc catcaagatc

8161 gcaggagcca gcgagaccat gctgatcgga gaccaaaacc agcttcccta tatcgaccga

8221 cacaacctgt ttcttctgaa atactgcaga ccccacaaaa tcacgtctgt cacgaaagaa

8281 ttgttgtgta cgtacagaaa cccgcaagac gtggcatatg ccctaggaga aatctatagc

8341 ggcatctact cggccaaaaa tctcacgcgc tccctgaagc tgaaggagta tggaggggct

8401 gttatcccga agttgaatga tacattgtat cttacccaca cccaggctga gaaggaactc

8461 ctaattggcc agggatacgg aaccaactcg ggctcttgca cccttactat acatgaagca

8521 caaggtcgaa cgttcgagac agtggtgatt gtacgtacaa catcaaaaaa aatccaatct

8581 gcttatgagc gtcccgcacg cggtggttgc gatctccaga cacacgaaga cctgtgtgta

8641 ctacgtggat aacacaaaga acgacgcagt agctcgtttt atccaacgtg cagagagagc

8701 aaccgacgct tccatcaggg actacaacct caaaatggcg atctggaacg gtgacgacgt

8761 aacgagggac agtatactcg ctttcgaagc ggggctggag gaagcctaaa acatccccct

8821 caaaacaaaa cacggacagc agccaccggc ggggccggcg gcaatgatat gacaagacaa

8881 aaagaataaa atgacaaaaa aaaaaaaaaa aaaaaa

**Spodoptera frugiperda**

**GESP01134032**

1 gtagagatta atgttcaaac cttctccgtg cagagaacac gtagagtctt gaacaagtaa

61 catctagctc ggcataaaaa ttgccgtaaa tagtaaattt ttgagataat cgcgtgtgat

121 cggtagaaag tgaatgttaa atactcaacg catcagcgac cgtgagggca aatttggaac

181 tcaacaggat caggaataaa gagccagcct cctggtactc aacagtataa cccaaaaagg

241 tcagtgtaat cacctcattc cggcgaaact gaacattggt atagccgcat cacaacctgc

301 aggcactact aagggcaaag agactctctg tccctaaaca aacatcaaaa caaataatac

361 catatgtttc ggtaataata tcaacaatta ttacaaatcg ccgcatcacc aaacgcacga

421 acgtgacctt cattcaggca cgcgccgtca gcacgttcgt atatatcaac atattaatca

481 aaggcgataa caactgacta ctgcaaaact gctgacacat caaattccat tgcacacaag

541 aattgcggtg ctaagggcta aatatccgac ccattgacat agaccgtaaa aactggtcgt

601 caacaattgg gaaaggcaac atctgtttgg ccccgcgcga ggaggagcgg acttcacaat

661 cgacgaacgg aacatcagaa ccaatcacac cagtggttct acacacgtcg ggccgtgcgc

721 gctacatcac cacaccccga ccactgcagg acatcagcgg accagtcgga tactatcata

781 cttcatagat taacaatacc aacaataatc cctagaaaat aaactaacat aatctataaa

841 atactaacaa acaacattgt aacaaaacaa acattctgtt agactagact gcatacacat

901 ttccccctct tttttatatt ttacacaaag actaaaaatg tctaccacta gcagcacacg

961 cggtaagcgc tcagccgcca tcacaatcac atcaccatct cctgtaaaaa aaaaagggac

1021 gagcattccg cggtccgggt gtgcgtcact gacttcgcgt acaccaaacc atttgatggc

1081 catggcaggg acgtcatcca tgaacgacgg atctacgact cgctcacctt cctgccaagc

1141 tgcagaagca ataaaaaaag tagaaatctg ggaacaggca gacatccttt cagacgacga

1201 agcctgggaa atcggatctc aggatgcttc agacttgcaa ggaagaaaga gcatgctaac

1261 cacagacaga caacgctcgg gcattaccat ggacctggct aacaaatatg ccaacgaaca

1321 gctgcagcgg ggcaaaacag cgctggaatc tgccgggaac atgaagcgag aatttaaagt

1381 aacggcaata gagtgtcttc agttccttta cgaaacttgt ctcgctcttt cagattccag

1441 gtcacgacat atgctaaacc tggagaagga aaggtctcgt catgcacggg aaattattgc

1501 aatcgagagg gcccacaaca aaaaaattgc tgaggtaaca aaagaactga cctcagaaat

1561 agcattggcc cgaaacgatt taacagtgag cctcaaagaa ataaagagta tccgtgcttg

1621 gctgggctat gagacaatgg aaccgtacaa gaggatagaa gacatcgaaa gaacaacaaa

1681 agagattaat gcaactgtga cgaaactaaa cctaagtcac aaatccccac agaaatctca

1741 aacagacctc aaatctcttg aagaaaaaca ggctaagttg ctcacgacag tacaaacatt

1801 gtcacagcaa ctagacgaac tgcgacgatg gcaacacaag gccatagaaa ataccaccac

1861 cctccagata gtaagccaag agattaatga taagttaggt tctcgtgagc aaaatgttcc

1921 agcagaaata acaaaccaaa gccagcaaaa aattgaggac gacatagtcg aacttaaaaa

1981 aactatgaac atcatagcgg atcacatcag agacgcctcc acactatcgg cacaagctcc

2041 tcaattagaa cagaatcttc agcctatcac cgagcgcctg gatgcagtat cgtcagaact

2101 gcggacaatg aggcagctca aagagcgaac gcctccccca cctgcacaca gcgtgtgtac

2161 ggaaatggca ttggcagaca ttgtcaaaca aatccgtcaa cctacgtatg cgcaagtggc

2221 atccaaacca cctgtccaca aaccaaacca cacccttata atcagttcaa ccgaccccaa

2281 aaacacaggg gacaatgtaa ttgaaaaaat aagggttgct ctggactgta aaaaaaccgg

2341 agcaaaagtg gaaaaggtga gaaaggctaa gaaccaaaag atagttatca gctgtagcac

2401 caaagaagac atgaagttgg tccaaagcca ggtccaaaag aaggacgacc tcaaagtgga

2461 agttgccaaa gcaagtaacc cactgctcag aatagcggat gtgctgtcct accatactga

2521 cgcagagcta gtagaactca tacttgccca aaacaaacat cttctgggtg acgtcagtat

2581 gcaagacaac atcataaggg tgaagtacag aaaaaaggca cgaaacccac atgagtgtca

2641 cccagtattg gaattgtcac cgggtaccca caagcgattc ctggaggccg gtaaaatcta

2701 tgtgggattg cagaggagac cagtgttcga ccaatcccca ctggtacaat gcaacaaatg

2761 tctggaatac ggtcacacaa aagccgtatg tcaggctaaa gaacatgtct gtagccactg

2821 tggagaccca cacacttggg agaaatgccc aaataggctt tcgaacaaac cgccgacgtg

2881 caggaattgc ttgagggcgc agggagttgg gccggagaca acgcataatg ccttcagcga

2941 agtctgtcga gaacgtcaaa aatgggatgc tatagcacgc tcccgcatat catattgcta

3001 aagttgacca ggcgaatcag gacgcaaccc cctcaaaggt gaacaacggc agaatgcgta

3061 tcatccaagc aaacctgcat cgctccaggc tagccactat agaattgcta caagttgcgg

3121 agaaaaaagg tatatctgta gcactggttc aggaacctta cgtagggaac actggtgaaa

3181 tgcaagaata ccctggcaca agaataattc agtgtactat gaatcgacaa aagccagtaa

3241 aagcggccgt aatcgttttc tcagaccagc tgaggactat acatgatcct cagcttgtaa

3301 cagaaactga agtagcaata ctgttggtct caggtagcct gcggcttgga gtgatatcgg

3361 tttaccttga gggggaccaa gacatacatc taaatttaac ccgcttaaag gagtccatat

3421 ctaaactgac cacaaaaaat atcatcgtag ctggagacgt gaacgcctgg agcccgtggt

3481 ggggcagcgc ctcggagaac cagaggggag cagattataa ttcgttccta aacgaaatgg

3541 acctccacat tctcaacaca ggggatacac caactttcga agcactcaga ggggaccgac

3601 ttttcacaag catagtggac gttacagcct gcagtacatc tttgctagca aaagttcaga

3661 actggatggt agatagaggc cttacaacct ctgaccacaa tgccataacg tttgacttgc

3721 agctggaaaa ggcattggaa cccccaaagc ccataacaac gagagtctac aacaccaaaa

3781 acgcaaactg gacaaaattt agatcacatc tgcgagattc tctagagaag aaacaaataa

3841 ctccagaaag tatcatgagt gttcagtcac ctgaagagct ggaaaccgtg attacgacct

3901 acataaatgc tatcacggag gtatgcgatg ccaccctacc caaaaataag ccgaggaaag

3961 ggaaatcaaa acccccgtgg tggacgagct ctttagagga tctcaaaact gacgtgcttc

4021 gaagaaaaag gaggataagg aacgcagctc cctctcgcaa acagttcgtc attgaggaat

4081 acttgcgggc aaaagaaaca tacacgtcaa aagccgatga agaacaaacg aagagttgga

4141 aggagttctg ctcgactcaa aagagggaaa gcatgtggga cgggatatac agggtcatca

4201 ggaaaacaac aaaatgtaga gaaaacaccc tgcttagaaa ttctgagggt caaacactga

4261 acccggacaa gtcagcggaa ttgttggcta aaacttttta ccttgatgac actacctcca

4321 cggatgatcc cggccatgct gaccttcgtg cactaacgga tggaagatgt ctaggtggga

4381 tggaggagct gtcggaaagc gaccctcact ttacacaggc agaactggat ttgattctca

4441 acgagatcaa tcctaaaaaa gcccccggcg ccgacggtct gacagctgat atctgcgcag

4501 aagctattta ttgtcgacgg gaggtgtttc ttgccatagc gaactcatgc ttagagcggg

4561 catacttccc aaagcaatgg aaatctgcgc atgttataat tctccgaaaa ccaggcaaag

4621 atgattactc taatccaaag tcctacagac caattggact tctgtctgtg tttggaaaaa

4681 tagttgagaa actgatgata ggcagaatcc agtggcacat ccttccaact cttcacaaaa

4741 accagtatgg atttctgcct caacgcggaa cggaagacgc actgtatgat ctggtcaacc

4801 gcataaaaac tgagatggat gaaaaaaaga tagtcgttct ggtgtcacta gacatagagg

4861 gcgcctttga caacgcctgg tggccggccc tgaaacatca actgatagcc aggaagtgtc

4921 cgaaaaacct gtatgccatg gtaaactcct acctcagcga acggagagtc aaagtcaact

4981 tcgccggagc cagcagcgaa aagggaacaa ataagggctg tgtacaaggg tccgtaggtg

5041 gacccacttt ttggaaccta atcctcgatt cgttgctaca cagactaaca ggtgaaggag

5101 tttattgcca ggcatttgcg gacgacgtcg ttctcttatt ctcgaaccat agggtgagcg

5161 cactggagca atcggtaaat agtgccttgg aaatagttgc agcatgggga gtcagcaaca

5221 agcttaggtt cggagcaagc aaaaccaacg ctatgctgct gacaaaaagg cttgtttttg

5281 aacccccaga actgttcatg acaggcacca gaataaacct tgtagatgag gtaaagctct

5341 tgggcctcac catagacaga aaactcacct tcaggtcaca catatccgca acgtgtaaaa

5401 aagctgcgga tatttacaaa cagctggcac gcgcagcaaa agtgacatgg ggtcttaatg

5461 gcgagataac gagaacaatc tacgtagcgg tcattgagcc gattgtactt tacgccgcaa

5521 acgtgtgggc cccagtgacg gagctacagt tgattaaaaa acaattaaat gctcttcaaa

5581 ggggatttgc acaaaaaatc tgcagagcgt atcgtacagt gtcgctcaca tcagcaacag

5641 tcttgtcggg tactctccca ctcgacttga gagtccagga gtgtgccagt ttgtatctaa

5701 ctaaaagagg attatctcta gactatttac cacctcataa ggagctggaa aggcaggtta

5761 actacctaga tcagccacat cccgcaaaat taatatctat caactatgag cttctggaga

5821 gcgatgactc tggaacacct ctcgtccgag gaattaccgg ccctcagata tacacagatg

5881 gaagcagaat tgagggaggc gtcggttctg ccttgacctg gtgggaggag ggtcgtgagt

5941 cggtgttctc cacttttagc ctggacccta cctgcacggt tttccaatct gaactatacg

6001 ctttgaacag agctgccaaa atggttcttg acagcagagc aactagagta aacatcatga

6061 gtgactctag gtcatcactc gacatcttaa agaaccccaa agttacccat cgtttggcca

6121 aagaaataaa gcagtgtgtg gagaccgtgg aggaacaggg aagagaaatc cggttctact

6181 ggctgagggc ccatgtcgga acggccggga atgagagggc agacgaatta gctaaaattg

6241 ctgccaaaaa gaaaaataac accacggcag actactgtat ggtacccatg tcctacgtca

6301 aaaagaaaat cagagacgaa accatccgaa agtggcagga caggtatacc acctccacta

6361 ctgggcaggt cactaagacg tttctcccgg atgtcaatga ggcatatcgt ttagtgagat

6421 cgacaaagtt gacacctgcc caagtacaag cgttgactgg acatggaggg atcgctgagt

6481 acctgcatcg attcaaatta aaagaaaatg caggttgcga atgcgactta accatcagtg

6541 agtcagtatg gcacattatt ctcgactgtc cccgttttct ggccgccaga tccagcttag

6601 aggctcaaat agatacaaat ctggatacct ccaatctgag tgccattcta gctgataaag

6661 tcaaaagacc gcacttcctg agctacatag acactatctt tagcagggca gctgaaagaa

6721 acagcacatt aaataataga caccaaatag ccaccttgga gaatcaatta acaactgcca

6781 cggcagtacc cgagacccct cgtactaatg agccgctcat aatcaatgag aagcaaatac

6841 tcctacatgg agaacgcggc accaaaggaa tacggttgcg aggtgtggcc ctattcatga

6901 acaccagcag cgaaagacta gggatagcct tctgcgaaaa aaggaatagc aaatggctca

6961 ctatttcacc gggcctagcc tccctgatca atggcagcac atccagaaca agcatgcgac

7021 gcaaagtcta tgacgcacta ccagacatga aactgttggg tcgaagctgt cgattgttgc

7081 gaacacgcaa taaaacaatc gcaatgtttt ccagcgtcga ctctataact tgttttgcac

7141 aagcatgtgg ggtgctgtca ggcgtgggtg agtggggaga cgtagaaggc atacattcca

7201 gaataattag cgtagacgct gcagtggtgg cttatgaaaa agggaaaacg gcagacctta

7261 tgggctgtat tagggcctca gaacatcatg aggtcatcgt gtacgaggac aggggggaag

7321 atttgagctt cctcaagagg tcagttcagc gccagctaag ggtggattct ccaagcggct

7381 cagagcgttt acagcaacaa tatttgcaac agagcgctct gcaacaacaa cagcgggcca

7441 ccgaaaggga aaataaaaaa ctacagtccc gaggcagaat gtgggcaata acaacatcca

7501 tatgctcagt aaccggactc attgcgtcac ctttcaggaa aaatgcatcc aagtccactc

7561 atagatcgga gagctgtccc gctcaaatac agtctgctca ttctaggtca tccccaacaa

7621 gagcagctat gggccacgtt tgtccaccga agcttcgaca agccaccact cccagagacc

7681 atctcataaa cgcttttctc gagtttgtcg cagtaataga agcaactaaa caggttaact

7741 tgcggaattg cgaagccatc ctgcagactt atctgcaggg taacgagctg atactggacg

7801 atagactcga agcagcagaa gcagtcatat acgacaataa tacatcgaga gtgcagagag

7861 gtacctctat aagtcgtagt atggcagctt acaacgccac tgcaggattt gtgtctctcg

7921 acgagaagga atcaaatcga tgcggagtag tgaagtttaa aactcccccc aacgattccc

7981 tagtggtggt tgccaagtgt accaggatta tgctagacga tgggatatta gaaatggcaa

8041 aatccatatc ggaatccgga ccggacggca taacctttga gagctggtgc actccgaggt

8101 ttcactggac gaacggggtc cctggatgcg gtaaaacgac ttggatagtg gatcaatttg

8161 atgcggacaa agatttaatt gtcaccacaa caactgaagc agcaaatgac ctcagaggca

8221 aactatcttg ccgatttggg ggactactag tcaaatctag ggttcgaaca atggcgtcca

8281 tcttagtaaa cggcttgaag gggcgagaga gctgcactcg actaatggta gacgaggctc

8341 ttatgaacca ctttggagcg gtggtgatgg ctgcccgaat aactttagcc aaggaggtcc

8401 tgctaattgg tgacattaat cagctcccgt tcattgatcg tgacaacctc ttccctctcc

8461 actacggccg ccctacacat atcacaccca taagtcagga gttgctgtgt acccacagga

8521 atccaatgga cgtggcctat gccctcaaca cagtttacag tggcatatac tcgtccaggt

8581 cgggcgtgag atccctggag acaaaaagat tctcgggagc gatgatccca aaatcactcc

8641 ccgacacctt atacctggta cacacccagg cagaaaagga atcccttaag aaccagggat

8701 acgggtcggg cgagggatca cgcattctca ctatacacga ggctcagggg ctttcgtccg

8761 cgaaggtggt agttgtgaac acagcaacaa agaggcagaa aattcatgac agcgtaccac

8821 atgcagtagt tgcaatatcg cgtcacacac atagttgcat ctactacacg gacaactgtg

8881 atgacgcgat aagcaaattg atcagaaggg ctgctgaaga gagcgaccat aggataatgg

8941 actacaacct gaaaatggcc atcaaacaca gagacgcggc agttctgggg gcgcttttgg

9001 gtgagaaatg ttccttggac taaaataaaa atttatatag tgggaggtaa tgtgtatgcg

9061 g
