# Supplementary material for: RNA helicase domains of viral origin in proteins of insect retrotransposons: possible source for evolutionary advantages
Source: PeerJ. 2017 Aug 16;5:e3673. doi: 10.7717/peerj.3673 (PMC5563155; doi:10.7717/peerj.3673)
Supplement: Supplemental Information 5 [file peerj-05-3673-s005.doc]

**Amino acid sequence alignment of the SF1H conserved domains encoded by insect LINE transposons.**

The alignment used to generate sequence logo (Fig. 5) is shown.

Andesiana lamellata NIRWVNGVPGCGKTTWVVKHF-DEEKDVVATTTTEAAKDLREKLAHRLGDR-----V-KTKVRTMASILVNGFKK---QEKCYRLTVDEALMNHFGTIVM

Tischeria quercitella SIYWINGVPGGGKTRWIITQF-KVGTDVIITSTTQSAADLKEKLSQRVGPN-----A-RSSVRTMASILVNGLRG---QGTCRRLIIDEALMNHFGSIVM

Eudarcia simulatricella RINWVNGVPGCGKTTWVIGHF-EAATDVVITTTTEAAKDLRERLALRLGAD-----A-TSKVRTMASMLVHGLRGR-DKDKCKRLIIDEALMNHFGAVVM

Caloptilia triadicae NFTWINGVPGCGKTTWVIDNI-DYKDDAVITTTLEAASDLTNRLAGRIGAV-----S-KSLVQTMASVLVNGLKA----PRRKRLFVDEALMNHFGAIVM

Plutella xylostella TIAWVNGVPGCGKTTWVVNNF-DVSKDTIITTTTEAAVDIRNRLAHRIGDM-----V-RTRVRTMASVLVNGFRE---HVGCQRLIIDEALMNHFGAIVI

Ostrinia nubilalis TITWVNGVPGCGKTTWVMSQI-DTSRDIIVTTTCEAAKDLREKLEPKIGAR-----A-KKRVRTMASLLVNGMSE---GETCTRIMVDEALMNHFGSIVM

Polyommatus icarus NYSWVNGVPGCGKTTWIINNF-NEETDVIITTTIEAAEDLKQRLSLRTGNK-----V-KDKVRTMASLLVNGTK-----GAYKRLIVDEALMNHFGSIVM

Lyssa zampa KFTWINGVPGCGKTTWLVSRF-DANEDVVVTTTTEAAKDLLEKLTRRIGKA-----A-KSKVRTLASVLVNGFREP-DKRRCNRLMVDEALMNHFGAIVM

Biston suppressaria KMQWVNGVPGCGKTTWVIRHF-EVDKDLIITTTTEAAKDLREKLSHRIGNL-----V-KTRVRTMASALVNGFGK----EGFSRLMVDEALMNHFGAIVM

Ceuthophilus sp TITLVQGVPGCGKTTNILSKA-TS-NDLILFATRESAEAFKIKYQEKHQAKNQ-KKINMEHIRTLHSFLINSHKL--QNHNYYRVIIDEALMAHCGELLL

Homalodisca vitripennis ELELVQGVLGCGKTTFIINKH-TK-GDLVLFPTREGATDFRNRLKQKDQTASY-NNI-KDSCRMIHSFIINSTNHIKSGGKYNRLIIDEALMLHTGEVLF

Nilaparvata lugens KIKWYDGVPGCGKSYFIVSHH-EPGKDLVLTQTRAGIKAIRETVIERYGRKHC-NRL-KLDYRTVGSYIINHNQN----KTYNRVFIDEALLMHAGYIGF

Homalodisca vitripennis IIKWIQGVPGCGKTTLLLNNY-NI-GDLILFPTRDTAIDFRQRFKRKHPDYAQ-SAC-NDTFRTVHSFLINSTQHLKRENTYKRLYIDEALMLHAGEILF

Gerris buenoi IIKWIDGVPGCGKTHYIVNEH-TPGQDLVLTQTRANLRDIKSSVEKNIKTKDN-RRI-DRDYRTVSSFIINGSDK-----DYQRVFIDEAVLMHAGYVGF

Planococcus citri YFKMVQGVPGCGKTTYILNNLHYLSLDLVLFPTREAAIDFRSRYCAQFGVPIDTAWL-KYRFRTIDSYLMGTD-----KNIYKRLFIDEASMIHFGQIVY

Andesiana lamellata AVKLSGASEIVLIGDVNQLPFLDRENLFKLRYTR-PNLVAGITQELHCTHRNPMDVAFALSEIYS--GIYSSKSSLSRVHSLKVKGYTGAQ---IPSTAQ

Tischeria quercitella IVRILQAEELLLIGDVNQLPFIDRDNLFKIKYHR-LPITPHKHQELHCTHRNPVDVAYALQNIYD--GIYSSST---TIKSLNITNFTGAN---ISRTQE

Eudarcia simulatricella AARLAGASEVTLIGDKNQLPYIDRENLFTMKYDR-PNLVAQITRELLCTHRNPMDVAYALSEVYN--GIYSTSP---KVKSLTLKTYKDAH---IPADSP

Caloptilia triadicae ASKLAKVEEVVLIGDVNQLPFIERENLFPVTYHR-PL-HFGIEKDLLCTHRNPMVVAFALRKIYG--GMYSPKR---QVRSLAKKGYRRST---VPEDLP

Plutella xylostella AARLSRASDIALIGDINQLPYIDRENLFELRYSR-PTLVANITQELLCSYRNPMDVAYALREVYS--GIYAATT---RIQSLQLKRFTDAA---IPKSQT

Ostrinia nubilalis AVQIAQASEALLIGDNNQLPYIDRNNLFPLLYNR-PNLITNVTKELLCTYRNPQDVAYALREIYS--GIYSAKT---LTRSLQLKGFTGAK---IPN-QE

Polyommatus icarus ANQLTGANDVILIGDINQLPFIERENLFKLNYTR-PNLVTGITQELSCTHRSPMDVAYALSMVYN--NIYSSKE---IVRSLKLTKYTGAR---IPKTDL

Lyssa zampa AARLAEAKEVLLIGDINQLPYIDRENLFPLFYYR-PTQLTTISQNLLCTHRNPMDVAYALREVYD--GIYSSVM---CVNSLKRGMYKGVN---IPKTLS

Biston suppressaria VSRLSGAGEIVLIGDVNQLPYIDRENLFEMRYHR-PNLVTKISQELLCTHRNPMDVAYALREIYS--GMYSSVC---RIKSLEQKRYKGAQ---IPNTLP

Ceuthophilus sp SAYTAGCKELELFGDQNQIRYINRTTHCTVRYADI-LQITDKQTCHNTSYRCTNSVAAILSSYYGNNGMKSIST---VKNEMEIRTYVSATQIQ---IPK

Homalodisca vitripennis ACALAGVKKALLVGDKQQIPFINRTTCNMIHY-DI-TKIAKTTTVLNFSYRCTNSVTTLLSPYYE-QGMATCNS---VENEVDSVYLDDLNKLH-LN-KS

Nilaparvata lugens IANLSKASEIIVVGDANQIPYIERSNYAT-RWHK-ISEFCEPFTKQTVTRRCPIDVCFVLSTVYE--NITTLNE---RATSILP-TYRNGE---YHLIQP

Homalodisca vitripennis AAVLSGADEVMLIGDCNQIPYINRTKNIEVKYHDI-TEIAKTTKTLNTTYRCIKSTTAIISKYCE-QGIKTTNK---IEKELELRQYSGLEGLNLIPEKN

Gerris buenoi IAELANAKEIILLGDANQIPYIERSALIS-EWSN-IARFCSPSKTLSVSKRCPMDVCFLLQGYYK--EILTVNT---KVHSIRP-PIADGS---FYQLKP

Planococcus citri AICKSSARIVIMIGDTHQIPYINRSPIPAIFHSSIPDALINETEYLSTSYRCTTTSSFLLCHLYK-HGMFTTST---VRREMKLHQYVSIE---QLPHIK

Andesiana lamellata NTLFLVHTQEEKASLISQGYG-SGEGSRTLTIHEAQGLTYDSVIIINTKSRRLQIHDSISHAVVAVSRHTVSCVYY

Tischeria quercitella NTLYLFHTQAEKESFKAEGYA-SGEGSLILTIHEAQGLSYKTVYIIKSLRKHTQIHNSVSHAVVAISRHTESCTYF

Eudarcia simulatricella NTLFLTHTQAEKELLKSEGFG-SGDKSRILTIHEAQGLTYESVVVIRVADRRTQLHDSVPHAVVAVSRHTLRCVYY

Caloptilia triadicae NTLYLVHTQAEKAALLSLGYE-KGEYSRLLTIHEAQGLTYEDVVILNTVEKKMRIHDSVSHAVVAISRHTRSCTYY

Plutella xylostella NTLFLTHTQEEKETLTSQGFG-EGTGSRVLTIHEAQGLTYESVIIIKTKD-KIKLHDSIPHAVVALSRHTSACTYY

Ostrinia nubilalis DTLYLVHTQAEKALLIGQGYG-TKTGSRTLTIHEAQGLTFREVVIVRTTSKKSHLLQSVPHAVVAISRHTDSCTYY

Polyommatus icarus NTLYLVHTQEEKAALTNTGYG-SGTDSRVLTIHEAQGLTSPSVIIIQTKSRKLAIHDSVPHAVVAISRHTNTCVYY

Lyssa zampa ETLYLVYTQDEKISLTNQGYG-SGEGSRLLTIHEAQGLTYKRVIIVNTMDKKLQLHDSVAHAVVAISRHTIECVYH

Biston suppressaria NTLFLVHTQEEKETLTNQGYG-SGTGSRILTIHEAQGLTYESVIVIKTKA-NMKLHESVPHAVVAISRHTGNFTYY

Ceuthophilus sp EAQVLTFTQSEKMELVHAGYK-------AMTIHEFQGKQAYTIVLIRTSNYNSEIYNSIPHCIVGISRHTKKFIYY

Homalodisca vitripennis LFKVLVFKQAEKRSLNSLGLN-------ASTIHEFQGKQASHVAVVRVNRTKDNIYDSIPHCPVAISRHTKVFRYY

Nilaparvata lugens DTLILTFTQEEKLMVGDT-MK-WREDVALHTIHEAQGLTHKNVILIRIKYKENEIYNSMPHAIVALSRHTETFRYL

Homalodisca vitripennis EYKYLVFKQSEKRELNKLGFK-------ASTIHEFQGRQAKNIAVVRTSARQEDIYESLHHCIVAVSRHTNSFLYI

Gerris buenoi DTLILTFTQNEKAMVTRCLENRCSEPLLVHTIHEAQGLTSKRVILIRINTSPLEIYNSIPHVIVALSRHTHSFRYL

Planococcus citri EAHYLTFTQQEKAFLAKHGFP-------TNTIHEFQGKQNGHIIVVRFIKDKAAIFESLPHIIVGLTRHREKLDYY
